# Synthesis and Anticancer Evaluation of Some Glycine Conjugated Hybrid Compounds Containing Coumarin, Thiophene and Quinazoline Moieties

**DOI:** 10.3390/ph18111627

**Published:** 2025-10-28

**Authors:** Nedime Çalışkan, Emre Menteşe, Fatih Yılmaz, Süleyman İlhan, Mustafa Emirik

**Affiliations:** 1 Department of Chemistry, Faculty of Art and Sciences, Recep Tayyip Erdogan University, 53100 Rize, Turkey; nedime_caliskan19@erdogan.edu.tr (N.Ç.);; 2 Department of Chemistry and Chemical Process Technology, Vocational School of Technical Sciences, Recep Tayyip Erdogan University, 53100 Rize, Turkey; 3 Department of Biology, Faculty of Engineering and Natural Sciences, Manisa Celal Bayar University, 45140 Manisa, Turkey

**Keywords:** quinazoline, coumarin, thiophene, glycine, prostate cancer (PC-3), breast cancer (MCF-7)

## Abstract

**Background/Objectives**: Cancer is one of the world’s leading causes of death. In 2022 alone, 9.74 million people died of cancer. It is estimated that this figure will rise to 10.4 million by 2025. Prostate and breast cancer are the most frequently diagnosed cancers in the world. **Methods**: Notably, compound **9f** displayed the highest activity against both prostate cancer (PC-3) and breast cancer (MCF-7) cell lines. It was seen that substitution on the coumarin ring had a positive effect on anticancer activity (except chlorine substitution at the 6th position of coumarin), while it had a negative effect on the selectivity index (the ratio of IC_50_ calculated for healthy and cancer cells). **Conclusions**: The findings are consistent with the results obtained in the Molecular Docking study. Molecular docking studies were performed to investigate the binding affinities of the synthesized compounds towards kinesin-associated motor protein EG5, Human Ribonucleotide Reductase and Human Topoisomerase II, confirming their potent in vitro cytotoxicity against cancer cell lines. In accordance with the findings of experimental studies, compound **9f** demonstrated the optimal docking binding scores.

## 1. Introduction

Cancer is one of the leading causes of death worldwide. Approximately 20 million new cases were detected and 9.74 million people died of cancer worldwide in 2022. According to the World Health Organization (WHO) and the International Agency for Research on Cancer (IARC), cancer cases in the world are expected to reach 35 million by 2050. This represents an increase of approximately 50% compared to 2022. At the same time, the number of people dying of cancer is expected to increase in a similar way. Also, cancer is the second most common cause of death worldwide. Therefore, scientists develop novel compounds which have anticancer activity, and significant numbers of them have been approved by the Food and Drug Administration (FDA) for treatment of cancer. However, drug resistance and undesirable side effects are the key factors limiting the effectiveness of current chemotherapy regimens. To overcome these challenges, there is a need for the development of new anticancer agents that can enhance potency and minimize the side effects [1,2,3].

Prostate cancer is one of the most diagnosed malignancies and the second largest cause of male mortality after lung cancer worldwide [4,5]. In 2022, there were 1.47 million new cases and 397 thousand deaths from prostate cancer. According to the International Agency for Research on Cancer (IARC), It is estimated that these numbers will increase to 1.57 million new cases and 417 thousand deaths, approximately [1]. After the development of cancer in the prostate, cancerous cells can spread to the lungs, pancreas, stomach and bones through angiogenesis. Angiogenesis is one of the reasons for the development of many diseases such as cancer [6]. It is an important mechanism that tumors use to grow and spread. Cancerous cells can promote angiogenesis to secure a blood supply that supports their growth and allows them to survive, especially as they increase in size [7,8,9].

Breast cancer is a type of cancer which starts in breast cells. If it is not controlled, the tumor cells can spread throughout the body and be fatal. There were 2.3 million new cases of breast cancer worldwide in 2022. It resulted in 670 thousand deaths among women. It can be seen in every woman at any age after puberty. In 2023, approximately one in eight women (approximately 13%) faced breast cancer in their lives [10]. The death rate of breast cancer was unchanged between 1930s and 1970s. However, survival rates increased in the 1990s owing to early detection and effective drug treatments [11].

The development of effective anticancer compounds aims to overcome some challenges associated with existing treatments, such as severe toxicity and drug resistance [12,13]. Molecular hybridization technique is a classical medicinal chemistry approach for obtaining new bioactive compounds through the combination of different bioactive fragments, creating a new hybrid entity endowed with enhanced affinity and potency compared to its parent compounds. Also, previous studies have shown that molecular hybridization could be a promising drug design strategy for cancer therapy [14].

**Figure 1:**
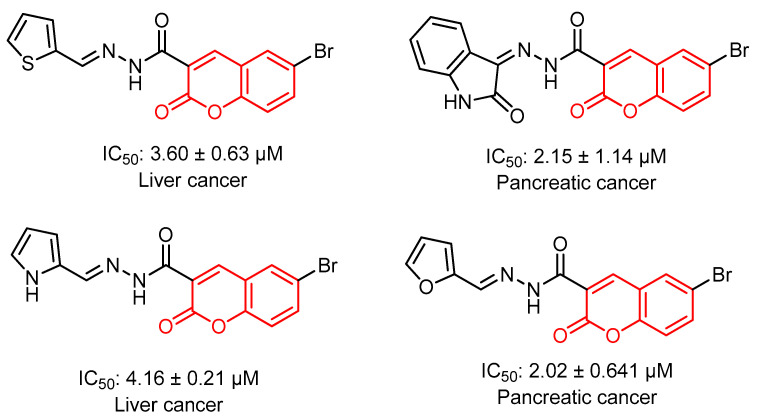
Some active anticancer coumarin derivatives.

Coumarin (2*H*-1-benzopyran-2-one) was first isolated from the Tonka bean (Dipteryx odorata) of the Fabaceae family in 1820 [15]. Coumarin derivatives are found as secondary metabolites in many plant species, most commonly in the families of Rutaceae, Apiaceae, Fabaceae and Asteraceae [16]. They can be isolated as novobiocin and aflatoxin from some microbial sources. Approximately 80% of the anticancer drugs in use are natural compounds [17]. Many researchers have extensively investigated the various mechanisms of coumarin-based anticancer agents, including molecular hybridizations [18]. In the search for new anticancer drugs, natural products are always an important source. Many coumarin derivatives obtained from natural products show important anticancer activity [19,20,21]. Moreover, the concept of molecular hybridization can provide efficient results in treatment with a coumarin moiety, as it may lead to the production of new anticancer drugs with lower toxicity, improved specificity, and increased efficacy [22]. Figure 1 shows some coumarin derivatives which possess anticancer activity [23].

**Figure 2:**
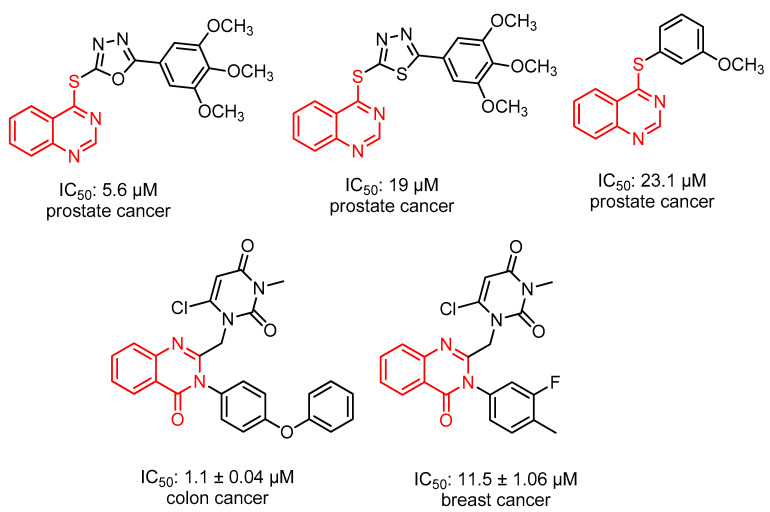
Some active anticancer quinazoline derivatives.

Quinazoline molecules have been identified as a preferred group of multi-acting therapeutic agents within the pharmaceutical and biological domains. The ease with which it can be prepared, in addition to its diverse pharmacological activity, has led to its significant importance among the various therapeutic agents available. The fundamental approach to the development of novel agents with anticancer activity involves the placement of different substituents at the 4, 6, and 7-positions of the quinazoline system [24,25]. According to the literature, many quinazoline derivatives have been found to have anticancer activity due to their high selectivity and low toxicity [26,27] (Figure 2).

**Figure 3:**
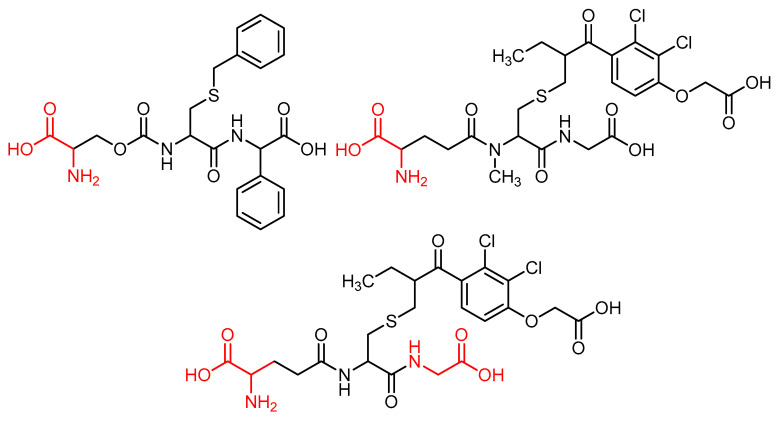
Some amino acid-conjugated compounds used against breast cancer.

Amino acids are among the most significant molecules in nature, given their role as the building blocks of proteins and as intermediates in metabolism [28]. Amino acid molecules bind together to form proteins. The configuration of these proteins is determined by the types of amino acids that they contain, as well as the order in which these amino acids are arranged. The human body contains twenty amino acids, which combine in various ways to form more than 40,000 proteins [29]. Amino acids have been shown to possess special physiological functions, including biocompatibility and cell affinity. These functions render them the most fundamental substances of the biological system. Interaction and selectivity against tumor cells can be increased or decreased through amino acid modification [30]. Amino acids have become interesting molecules to be used in cancer therapy, with the aim of reducing the need for genotoxic agents [31]. As demonstrated in the literature, amino acid-conjugated compounds have the potential to act as chemotherapeutic agents. In addition, amino acid conjugation has been shown to increase antitumor activity in hybrid compounds [32,33]. Figure 3 shows some amino acid-conjugated anticancer compounds used against breast cancer.

Considering our experience in design and synthesis of novel hybrid molecules [34,35,36,37,38,39,40,41,42], we focused on the synthesis of novel bioactive molecules containing promising hybrids such as coumarin and quinazoline moieties, with the hope of revealing novel anticancer agents that are more active and have fewer side effects. The results of our literature research show that there are limited studies on the design, synthesis and anticancer evaluation of quinazoline- and coumarin-containing hybrids. The main aim of the present work is to synthesize novel hybrid compounds containing quinazoline, thiophene and coumarin cycles, which were found as bioactive compounds in our previous works [34,37,38,43,44], and determine their anticancer properties on prostate cancer (PC-3) and breast cancer (MCF-7) cell lines. Interestingly, the findings of the present study demonstrate that substitution on the coumarin ring enhances anticancer activity yet concomitantly diminished the selectivity index value. Also, the substitution at the 8th position of coumarin resulted in a greater increase in activity than the substitution at the 5th position. The findings presented here align with the results obtained from the molecular docking studies.

## 2. Results and Discussion

### 2.1. Chemistry

**Scheme 1:**
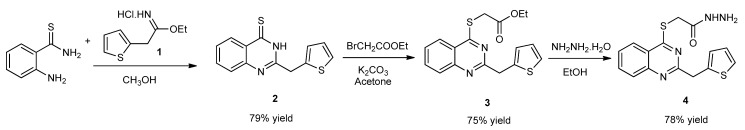
Synthetic route for compound **4**.

The synthetic routes for preparing target hybrid molecules were carried out in three steps. In the first step, firstly, ethyl 2-(thiophen-2-yl)acetamide hydrochloride (**1**) was prepared as described in the literature [43]. Secondly, 2-aminothiobenzamide was reacted with compound **1** to obtain 2-(thiophen-3-ylmethyl)quinazoline-4(3*H*)-thione (**2**). Then, compound **2** was treated with ethyl bromoacetate to obtain ethyl {[2-(thiophen-2-ylmethyl)quinazolin-4-yl]sulfanyl}acetate (**3**). Lastly, compound **3** was reacted with hydrazine monohydrate to obtain 2-{[2-(thiophen-2-ylmethyl)quinazolin-4-yl]sulfanyl}acetohydrazide (**4**), which is the first intermediate for the target hybrid compounds (Scheme 1).

**Scheme 2:**
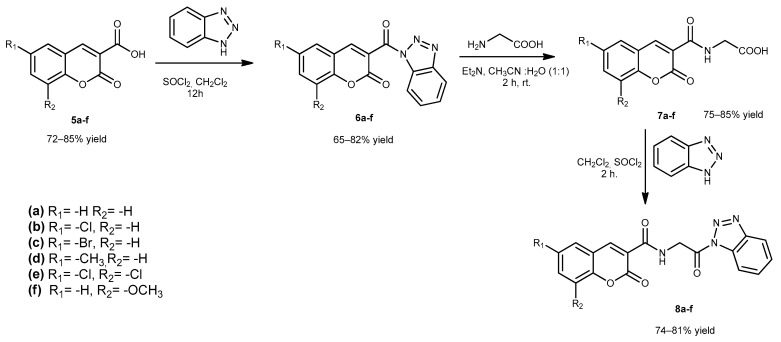
Synthetic route for compounds **8a**–**f**.

In the second step, firstly, compounds **5a**–**f** and **6a**–**f** were obtained according to our previous studies [34]. Then, compounds **6a**–**f** were reacted with glycine to obtain compounds **7a**–**f**. Then, compounds **7a**–**f** were reacted with 1*H*-benzotriazole in dichloromethane to obtain glycine-conjugated coumarin benzotriazole compounds **8a**–**f**, which are the second intermediate for the target compounds (Scheme 2).

**Scheme 3:**
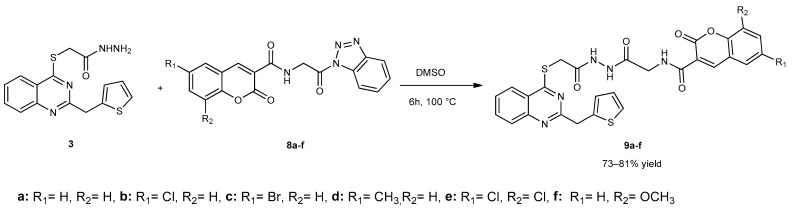
Synthetic route for compounds **9a**–**f**.

In the last step, compounds **7a**–**f** were reacted with compound **3** in dimethyl sulfoxide to obtain glycine-conjugated hybrid compounds containing quinazolinone, thiophene, and coumarin moieties (Scheme 3).

Spectral analyses of target compounds are suitable for the proposed structures. Infrared (IR) spectra of compounds **9a**–**f** gave NH signals between 3300 and 3100 cm^−1^, C=O signals between 1750 and 1610 cm^−1^, and C=N signals at about 1560 cm^−1^. The ^1^H NMR spectra of these compounds showed three NH signals between 11.80 and 9.20 ppm. Coumarin C_4_-H was shown at about 8.90 ppm in ^1^H NMR spectra of these compounds. Three CH_2_ signals were found at about 4.50, 4.10, and 4.00 ppm, which belong to SCH_2_, NCH_2_, and CH_2_ protons, respectively. The ^13^C NMR data of these compounds showed suitable signals with the proposed structures. The C=N signal belonging to the quinazolinone C_4_ carbon atom was shown at about 170 ppm. Three C=O signals were shown between 170.00 and 157.00 ppm. The other aromatic signals were shown in suitable range and number. When the mass spectra of these compounds were examined, the signals originating from the ^79^Br and ^81^Br isotopes found in compound **9b** and the ^35^Cl and ^37^Cl isotopes found in compounds **9c** and **9e** were observed in suitable proportions and in accordance with the [M + H]+, [M + Na]+ and [M + K]+ signals. In addition, all the compounds gave suitable elemental analysis results indicating the calculated ones.

### 2.2. Anticancer Activity Study

The IC_50_ values of the target compounds against prostate cancer (PC-3), breast cancer (MCF-7), and human embryonic kidney (HEK-293) cell lines are shown in Table 1. The IC_50_ values were determined in the range of 8.7 ± 2.4 to 197.6 ± 1.5 µg/L for the HEK-293 healthy human embryonic kidney cell line, 14.7 ± 1.4 to 85.5 ± 1.1 µg/L for prostate cancer (PC-3), and 16.5 ± 1.2 to 104.9 ± 0.9 µg/L for breast cancer (MCF-7). Some of the synthesized compounds were found to be more active than Cisplatin. Among the synthesized compounds, the compound that showed the best results in terms of toxic effects on healthy human embryonic kidney cell lines is **9f**; the anticancer activity ranking on human prostate cancer cells (PC-3) is **9f**, **9e**, **9d**, **9c**, **9a,** and **9b**; and the anticancer activity ranking on breast cancer cells (MCF-7) is **9f**, **9e**, **9c**, **9d**, **9a,** and **9b**. Compound **9f** showed the best activity against prostate cancer (PC-3) and breast cancer (MCF-7) cell lines.

Conventional chemotherapy drugs have been employed in the treatment of cancer for a considerable period; however, they have been associated with significant challenges and deleterious side effects on normal cells. While these drugs are effective in targeting rapidly dividing cancer cells, there is also an unfortunate consequence of destroying healthy cells. It is important to note that the non-specific targeting of traditional chemotherapy drugs can affect both cancer cells and normal cells, which may reduce the effectiveness of the treatment and increase the risk of side effects. Another challenge that has the potential to reduce the effectiveness of treatment is the development of drug resistance in cancer cells. The objective of drug development for cancer treatment is therefore to selectively target cancer cells while sparing healthy cells, thereby reducing the impact on normal tissues. In this study, HEK-293 cells were utilized to investigate the cytotoxic effects of the synthesized compounds on normal cells. In contrast to cisplatin, all compounds (except for **9f**) exhibited higher IC_50_ values in HEK-293 cells compared to cancer cells. This finding suggests that these products are less toxic to normal cells yet retain their efficacy against cancer cells. In order to assess the cancer cell-specific effects of the synthesized compounds, the selectivity indices (SI) of the compounds were calculated by dividing the IC_50_ values for normal cells by the IC_50_ values for cancer cells. Within the domain of anticancer activity, an elevated selectivity index signifies a heightened propensity of the compound in question to interact with cancer cells, in comparison to normal cells. This finding indicates that the compound is more effective in inhibiting or killing cancer cells while having less toxicity or minimal effect on normal cells. The SI values of compounds **9a**, **9c**, **9d**, **9e**, **9b,** and **9f** in PC-3 cells were calculated as 3.22, 2.51, 2.14, 1.98, 1.57, and 0.59, respectively, indicating high selectivity against prostate cancer cells, and the SI values of **9a**, **9c**, **9e**, **9b**, **9d,** and **9f** in MCF-7 cells were 2.45, 1.68, 1.49, 1.29, 1.24, and 0.53, respectively, indicating that compounds **9a** and **9c** are selective against breast cancer cells.

The SI values of compounds **9a**, **9c**, **9d**, **9e**, **9b,** and **9f** in PC-3 cells were calculated as 3.22, 2.51, 2.14, 1.98, 1.57, and 0.59, respectively, indicating high selectivity against prostate cancer cells, and the SI values of **9a**, **9c**, **9e**, **9b**, **9d,** and **9f** in MCF-7 cells were 2.45, 1.68, 1.49, 1.29, 1.24 and, 0.53, respectively, indicating that compounds **9a** and **9c** are selective against breast cancer cells. This finding indicates that compound **9f** is more effective in inhibiting or killing cancer cells while having less toxicity or minimal effect on normal cells. Although compound **9f** exhibited the strongest cytotoxic activity among the tested derivatives, its relatively low selectivity index (SI) indicates a limited therapeutic window, suggesting that it may also affect non-cancerous cells. This feature reduces its immediate clinical attractiveness compared with compounds showing both high potency and higher selectivity. Nevertheless, **9f** may serve as a promising lead scaffold that requires further structural optimization to enhance selectivity while maintaining its anticancer efficacy.

### 2.3. Structure-Activity Relationship (SAR) Study

**Figure 4:**
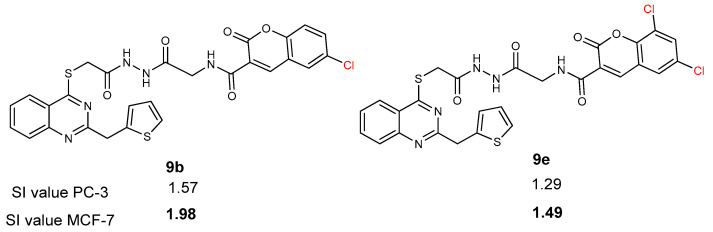
SI value of compounds **9b** and **9e**.

Considering the anticancer activity results, it was seen that most of the synthesized compounds have more selectivity index (SI) value than cisplatin. Only compound **9f** showed lower SI value than cisplatin. When the SI of compounds **9a**–**f** was investigated, the substitution of the coumarin ring negatively affected the SI of these compounds against both prostate (PC-3) and breast (MCF-7) cancer cell lines. Additionally, these substitutions resulted in increasing the interaction between synthesized compounds and healthy cells. This can be seen by looking at the IC_50_ values of compounds **9a**–**f** against Human Embryonic Kidney (HEK-293). The highest selectivity index against both prostate (PC-3) and breast (MCF-7) cancer cell lines was seen in compound **9a** with the value of 3.22 and 2.45, respectively. The second highest selectivity was seen in compound **9c** with the value of 2.51 and 1.68, respectively. The addition of chlorine group to coumarin ring in compound **9** resulted in the increase in selectivity index for both prostate (PC-3) and breast (MCF-7) cancer cell lines. It can be seen from compound **9b** (which has one Cl atom on coumarin ring) and **9e** (which has two Cl atoms on coumarin ring) (Figure 4). Also, the addition of methoxy group to the coumarin ring in compound **9** decreased the SI of both prostate (PC-3) and breast (MCF-7) cancer cell lines. It can be seen from the SI values of compound **9f** as 0.59 and 0.53, respectively.

**Figure 5:**
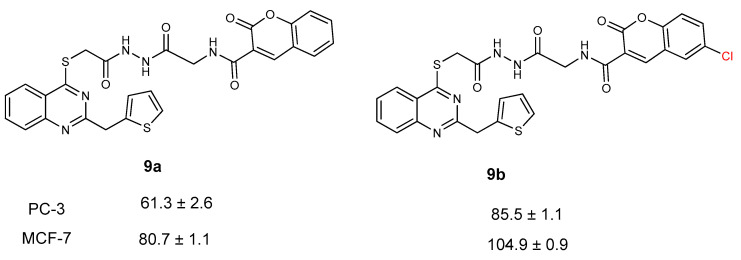
Anticancer activity of compounds **9a** and **9b** against PC-3 and MCF-7 cell lines.

According to the anticancer activity results, compound **9f** showed the highest activity against prostate and breast cancer cell lines, with an IC_50_ value of 14.7 ± 1.4 and 16.5 ± 1.2 µg/L. When the activity of compounds **9a**–**f** was investigated, the addition of chlorine at the 5th position of the coumarin ring decreased the anticancer activity against both prostate and breast cancer cell lines. It can be seen from the activity of compounds **9a** and **9b** (Figure 5).

**Figure 6:**
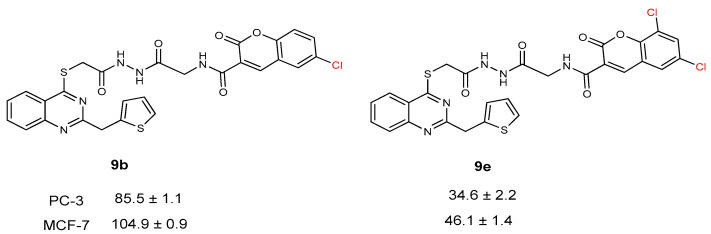
Anticancer activity of compounds **9b** and **9e** against PC-3 and MCF-7 cell lines.

When the activity of compounds **9b** and **9e** was compared, the addition of two chlorine atoms at the 5th and 7th position of the coumarin ring enhanced the anticancer activity against both prostate and breast cancer cell lines (Figure 6).

**Figure 7:**
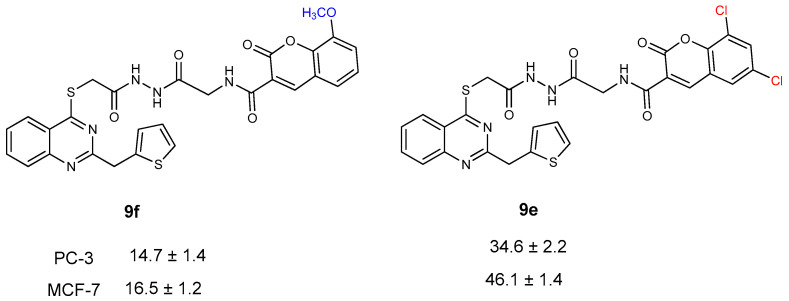
Anticancer activity of compounds **9f** and **9e** against PC-3 and MCF-7 cell lines.

When the activity of compounds **9a**–**f** was examined, it was observed that substitution at the 7th position of the coumarin ring positively affected the anticancer activity against both prostate and breast cancer cell lines. Because of this, compounds **9f** and **9e** seem to be the most effective against prostate and breast cancer cell lines (Figure 7).

**Figure 8:**
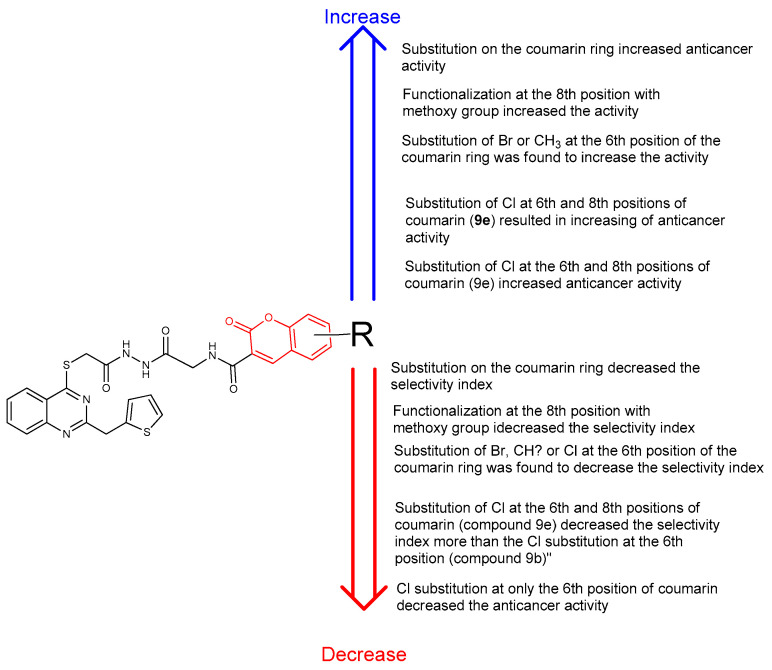
Summarized SAR of the synthesized compounds.

The summarized structure–activity relationship (SAR) study of the novel compounds **9a**–**f** is presented in Figure 8.

### 2.4. Molecular Docking Study

Molecular docking studies were utilized to understand the mechanism of anticancer activity of newly synthesized compounds, labeled **9a** through **9f**, against three distinct cancer-related protein targets. The results of the docking study were analyzed via binding affinity and interaction modes at the target protein binding sites. Our motivation in identifying target proteins was focused on elucidating the in vitro anticancer activity of the synthesized compounds. To this end, kinesin related motor protein EG5, Human Ribonucleotide Reductase (RNR), and Topoisomerase II (Topo II) were selected due to their critical role in the biological processes of cellular proliferation and DNA replication. EG5, a kinesin motor protein, is recognized for its essential role in mitotic spindle formation, a fundamental process in cell division [45]. Human Ribonucleotide Reductase is identified as a key enzyme in the synthesis pathway of deoxynucleotides, crucial building blocks for DNA [46]. Topoisomerase II is a well-known enzyme involved in managing DNA topology, facilitating processes like replication and transcription [47]. Due to their indispensable roles in the uncontrolled proliferation typical of tumor cells, these proteins have been widely investigated as therapeutic targets in the field of oncology. Inhibition of EG5 was found to induce mitotic arrest, while disruption of Human Ribonucleotide Reductase function was shown to impair DNA synthesis in rapidly dividing cells. Similarly, the inhibition of Topoisomerase II activity was understood to lead to DNA damage and subsequent cell death in cancer cells.

The results of the docking simulation are presented in Table 2. A comprehensive analysis of the generated scores revealed that compound **9f** consistently exhibited the most favorable predicted binding affinities across all the investigated protein targets. Specifically, for the EG5 target, a binding score of −11.881 was recorded for compound **9f**. Against Human Ribonucleotide Reductase, compound **9f** achieved a score of −9.088. Furthermore, for Topoisomerase II, compound **9f** demonstrated a notably strong predicted affinity, with a score of −12.694. In contrast to the vitro experiments, compound **9e** generally displayed the least favorable scores within the series, indicating a comparatively weaker predicted binding across the targets. Variations in the relative ranking of other compounds were observed, depending on the specific protein target, suggesting diverse binding preferences within the series.

Docking scores of the synthesized compounds showed a moderate positive correlation with the in vitro IC_50_ values on both PC-3 and MCF-7 cell lines (r = 0.57–0.63) (Table 3). However, the correlations did not reach statistical significance (*p* > 0.05), which can be attributed to the multifactorial nature of cellular activity, where permeability, metabolism, and off-target effects play crucial roles beyond binding affinity. Due to the nature of the docking study, a strict quantitative correlation between IC_50_ values and docking scores is not always achieved. This limitation arises from the inherent nature of molecular docking, which primarily estimates binding affinity and does not account for critical factors influencing cellular activity, such as membrane permeability, metabolic stability, or off-target effects. Nevertheless, in our study, the most active compound (**9f**) consistently exhibited relatively higher binding scores across different targets, supporting the qualitative consistency between the in silico and in vitro findings.

**Figure 9:**
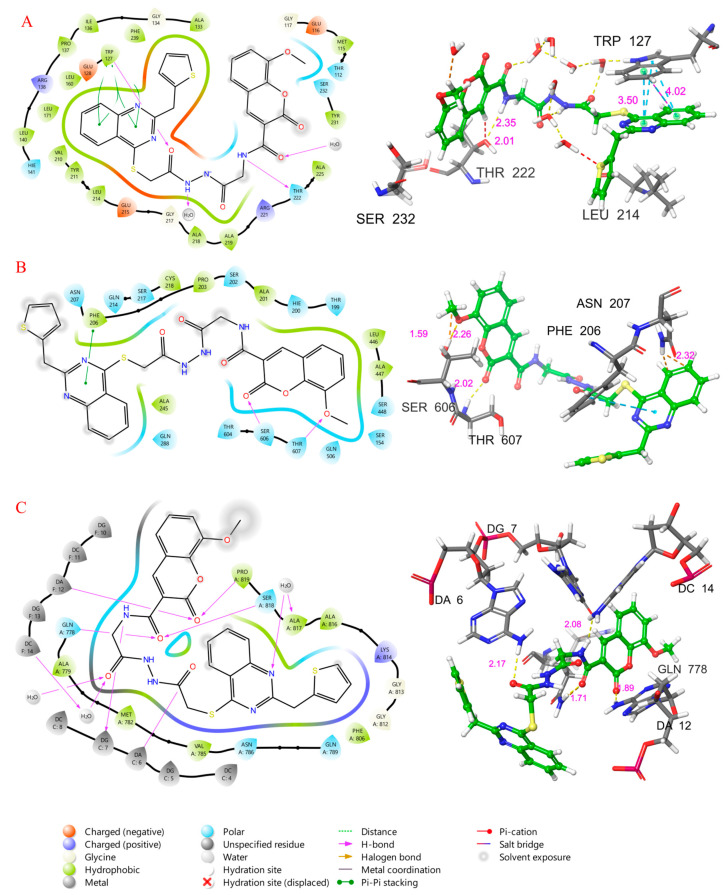
Two-dimensional and 3D ligand interaction diagram of compound **9f** at the binding site of EG5 (**A**), Human Ribonucleotide Reductase (**B**), and Topoisomerase II (**C**).

The interaction diagram showing the binding modes of compound **9f** at the active site of kinesin-associated motor protein EG5 is given in Figure 9A**,** and the main non-covalent interactions contributing to the stabilization of the ligand-protein complex were analyzed. Hydrogen bonds are formed between the NH group of the amide moiety of compound **9f** and Thr 222, as well as between the amide carbonyl and Trp 127. Water-bridged hydrogen bonding interactions were clearly observed in the binding pocket. It was observed that the Quinoxaline core of compound **9f** formed pi-pi stacking interactions with Trp 127. Hydrophobic interactions played an important role in the anchoring of compound **9f** to the binding site. The methoxy oxygen of the ligand is surrounded by the polar pocket, while the amid carbonyl, quinoxaline and thiophene rings are seen to be in the hydrophobic pocket. These interactions contribute to the stability of the ligand-protein complex and provide insights into the molecular basis of binding affinity.

A detailed two-dimensional interaction diagram depicting the binding pose (Figure 9B) of compound **9f** within the active site of Human Ribonucleotide Reductase was thoroughly analyzed to elucidate the critical non-covalent interactions that contributed to the stability of the ligand-protein complex. Hydrogen bonding interactions were observed as significant contributors to the binding stability. Specifically, the carbonyl oxygen atom from the coumarin ring system of the ligand was found to engage in a hydrogen bond with the backbone NH group of Ser 606. Another hydrogen bond was formed between the methoxy oxygen of the coumarin moiety and the side chain OH group of Thr 607. Pi-pi stacking interactions were also identified as playing a role in the ligand’s orientation within the active site. The quinoxaline ring of compound **9f** was observed to participate in a pi-pi stacking interaction with Phe 206. Hydrophobic interactions were extensively noted, indicating a substantial contribution to the overall binding. These interactions were represented by the surrounding green envelopes and involved numerous residues within the active site, including Cys 218, Pro 203, Ala 201, Leu 446, Ala 447, and Ala 245. Portions of the ligand, specifically around the quinolone and coumarin systems, were also seen to be involved in polar contacts, indicated by light blue lines. These diverse non-covalent interactions collectively contributed to the strong predicted binding affinity of compound **9f** to this vital enzyme, providing a molecular basis for its potential inhibitory activity.

The binding pose of compound **9f** within the active site of the Human Topoisomerase II enzyme, given in Figure 9C, was analyzed to elucidate the specific ligand-protein and ligand-DNA interactions contributing to the stability of the ternary complex. Hydrogen bonding interactions were observed to be pivotal in anchoring compound **9f** within the active site. The nitrogen atom of the quinoxaline ring in compound **9f** was shown to form water-bridged hydrogen bonds with the backbone carbonyls of Ala 817. Direct hydrogen bonds were identified; one carbonyl oxygen from the coumarin-like ring system of the ligand was seen to interact with Pro 819 and the DA F:12 DNA bases. Another carbonyl oxygen formed a hydrogen bond with Gln 778 and Ser 818. The amid carbonyl of the ligand also formed a direct hydrogen bonding interaction with the DA C:6 DNA bases. The NH group of compound **9f** also formed hydrogen bonding interactions with the DNA bases DG C:7. Hydrophobic interactions were extensively distributed throughout the binding pocket, contributing substantially to the overall complex stability. These multi-faceted interactions underscored the predicted stability of compound **9f** within the Topoisomerase II-DNA complex, providing a molecular foundation for its potent inhibitory activity against this vital anticancer target.

#### Comparative Binding Affinity Analysis

To assess the biological relevance of the docking results, we included a comparative analysis using clinically approved or clinically investigated anticancer agents as reference ligands for each protein target. For this purpose, ispinesib (evaluated in clinical trials for prostate cancer), gemcitabine (FDA-approved for breast cancer), and etoposide (FDA-approved for various cancers) were selected as reference molecules for the 6G6Y, 5TUS, and 3QX3 structures, respectively [48,49,50].

All the compounds, including the references, were subjected to the same docking protocol to ensure consistency in scoring. As presented in Table 2, compound **9f** exhibited higher binding scores than both gemcitabine and etoposide, and a comparable score to ispinesib, across the respective targets. These results suggest that **9f** may form strong and specific interactions with the target binding sites, similar to or better than those of established anticancer agents.

The high docking affinity of **9f** is further supported by its potent in vitro cytotoxic activity against both PC-3 and MCF-7 cell lines. Together, these findings reinforce the potential of **9f** as a promising multi-target anticancer candidate.

## 3. Materials and Methods

### 3.1. Experimental Synthesis

#### Synthesis of Compounds **9a**–**f**

A mixture of compound 3 (3.30 g, 0.01 mol) and the corresponding compound **8a**–**f** in dimethyl sulfoxide (DMSO) (25 mL) was subjected to heating in an oil bath for a period of 6 h at 100 °C. Following the conclusion of the reaction (which was monitored by thin layer chromatography (TLC), ethyl acetate:hexane, 3:1), the mixture was poured into an ice-water mixture, yielding a yellow solid. The filtrate was subjected to a series of treatments, including filtration and washing with hot water and with hot ethanol. The objective of the process was to obtain pure **9a**–**f**.

**2-Oxo-*N*-(2-oxo-2-{2-[(quinazolin-4-ylsulfanyl)acetyl]hydrazinyl}ethyl)-2*H*-chromene-3-carboxamide** (**9a**). Yield 0.41 g, 73*%*; M.P. 255–256 °C. FT-IR (KBR) *ν_max_*, cm^–1^: 3273, 3228, 3138 (NH), 1716, 1665, 1645, 1611 (C=O), 1566 (C=N). ^1^H-NMR (400 MHz, DMSO-*d*_6_), δ, ppm: 11.74 (s, 1H, NH), 9.83 (s, 1H, NH), 9.25 (s, 1H, NH), 8.94 (s, 1H, H-4 coumarin), 8.02–7.84 (m, 2H, ArH), 7.75 (t, 2H, *J* = 8 Hz, ArH), 7.52–7.45 (m, 2H, ArH), 7.28–6.98 (m, 5H, ArH), 4.51 (d, 2H, *J* = 4 Hz, SCH_2_), 4.13 and 4.22 (s, 2H, NCH_2_), 4.07 (s, 2H, CH_2_). ^13^C-NMR (100 MHz, DMSO-*d*_6_), δ, ppm: 35.84 (CH_2_), 42.05 (NCH_2_), 51.06 (SCH_2_), 116.51, 116.60, 118.75, 118.93, 125.63, 126.12, 127.25, 127.30, 130.85, 131.80, 137.19, 137.65, 144.07, 148.38, 154.43 (Ar-C), 157.24 (C=O), 160.90, 161.33 (C=O coumarin), 167.62 (C=N). LC-MS, *m*/*z*: 560.0 [M + H]^+^, 582 [M + Na]^+^. Calcd for C_27_H_21_N_5_O_5_S_2_: elemental analysis: C, 57.95; H, 3.78; N, 12.51%; found C, 57.87; H, 3.71; N, 12.43.

**6-Bromo-2-oxo-*N*-(2-oxo-2-{2-[(quinazolin-4-ylsulfanyl)acetyl]hydrazinyl}ethyl)-2*H*-chromene-3-carboxamide** (**9b**). Yield 0.47 g, 75*%*; M.P. 265–266 °C. FT-IR (KBr) *ν_max_*, cm^–1^: 3258, 3225, 3200 (NH), 1742, 1662, 1641, 1612 (C=O), 1561 (C=N). ^1^H-NMR (400 MHz, DMSO-*d*_6_), δ, ppm: 11.78 (s, 1H, NH), 9.83 (s, 1H, NH), 9.25 (s, 1H, NH), 8.92 (s, 1H, H-4 coumarin), 8.17 (d, 1H, *J* = 4 Hz, ArH), 7.90 (d, 1H, *J* = 8 Hz, ArH), 7.56 (d, 1H, *J* = 4 Hz, ArH), 7.44 (d, 1H, *J* = 8 Hz, ArH), 7.23 (t, 1H, *J* = 12 Hz, ArH), 7.09 (d, 1H, *J* = 8 Hz, ArH), 7.07 (s, 1H, ArH), 6.97 (t, 1H, *J* = 8 Hz, ArH), 6.87 (t, 1H, *J* = 8 Hz, ArH), 6.83 (d, 1H, *J* = 4 Hz, ArH), 4.50 (d, 2H, *J* = 4 Hz, SCH_2_), 4.18 (s, 2H, NCH_2_), 4.08 (s, 2H, CH_2_). ^13^C-NMR (100 MHz, DMSO-*d*_6_), δ, ppm: 35.84 (CH_2_), 40.93 (NCH_2_), 56.46 (SCH_2_), 109.98, 115.79, 116.85, 117.12, 118.88, 119.84, 119.88, 120.74, 126.10, 127.24, 127.29, 132.68, 137.19, 137.66, 144.11, 145.17, 153.44 (Ar-C), 153.57 (C=N), 155.85, 157.24 (C=O), 161.03 (C=O coumarin), 167.54 (C=O). LC-MS*, m*/*z*: 639.3 [M(Br^79^) (Br^81^)+H]^+^, 641.3 [M(Br^81^)+H]^+^. Calcd for C_27_H_20_BrN_5_O_5_S_2_: elemental analysis: C, 50.79; H, 3.16; N, 10.97%; found C, 50.71; H, 3.10; N, 10.90.

**6-Chloro-2-oxo-*N*-(2-oxo-2-{2-[(quinazolin-4-ylsulfanyl)acetyl]hydrazinyl}ethyl)-2*H*-chromene-3-carboxamide** (**9c**). Yield 0.44 g (75*%*); M.P. 255–258 °C. FT-IR (KBr) *ν_max_*, cm^–1^: 3315, 3234, 3140 (NH), 1724, 1645, 1612 (C=O), 1568 (C=N). ^1^H-NMR (400 MHz, DMSO-*d*_6_), δ, ppm: 11.78 (s, 1H, NH), 9.86 (s, 1H, NH), 9.34 (d, 1H, *J* = 4 Hz, NH), 8.92 (s, 1H, H-4 coumarin), 8.16 (t, 1H, *J* = 8 Hz, ArH), 7.90 (d, 1H, *J* = 8 Hz, ArH), 7.79 (d, 1H, *J* = 4 Hz, ArH), 7.58 (d, 1H, *J* = 4 Hz, ArH), 7.46 (d, 1H, *J* = 4 Hz, ArH), 7.32 (d, 1H, *J* = 4 Hz, ArH), 7.24 (t, 1H, *J* = 4 Hz, ArH), 7.10 (s, 1H, ArH), 6.98 (d, 1H, *J* = 4 Hz, ArH), 6.87 (t, 1H, *J* = 8 Hz, ArH) 4.52 (d, 2H, *J* = 8 Hz, SCH_2_) 4.19 (s, 2H, NCH_2_), 4.09 (s, 2H, CH_2_). ^13^C-NMR (100 MHz, DMSO-*d*_6_), δ, ppm: 35.97 (CH_2_), 42.12 (CH_2_), 43.64 (CH_2_), 118.62, 119.77, 120.40, 127.40, 129.32, 129.70, 134.02, 134.27, 147.76, 153.11 (Ar-C), 160.36, 160.50 161.14 (C=O), 161.64 (C=O coumarin), 167.86 (C=N). LC-MS, *m*/*z*: 635 [M (Cl^37^)+K]^+^. Calcd for C_27_H_20_ClN_5_O_5_S_2_: elemental analysis: C, 54.59; H, 3.39; N, 11.79%; found C, 54.51; H, 3.32; N, 11.73.

**6-Methyl-2-oxo-*N*-(2-oxo-2-{2-[(quinazolin-4-ylsulfanyl)acetyl]hydrazinyl}ethyl)-2*H*-chromene-3-carboxamide** (**9d**). Yield 0.44 g (78*%*); M.P. 266–267 °C. FT-IR (KBr) *ν_max_*, cm^–1^: 3308, 3141 (NH), 1721, 1708, 1644, 1614(C=O), 1571 (C=N). ^1^H-NMR (400 MHz, DMSO-*d*_6_), δ, ppm: 11.73 (s, 1H, NH), 10.50 and 9.82 (s, 1H, NH), 9.25 (d, 1H, *J* = 4 Hz, NH), 8.85 (s, 1H, H-4 coumarin), 7.87 (d, 1H, *J* = 8 Hz, ArH), 7.76 (s, 1H, ArH), 7.57–7.38 (m, 4H, ArH), 7.18–6.93 (m, 4H, ArH), 4.50 (d, 2H, *J* = 4 Hz, SCH_2_), 4.22 and 4.12 (s, 2H, NCH_2_), 4.06 (s 2H, CH_2_), 2.37 (s, 3H, CH_3_). ^13^C-NMR (100 MHz, DMSO-*d*_6_), δ, ppm: 20.69 (CH_3_), 35.83 (CH_2_), 42.03 (NCH_2_), 43.46 (SCH_2_), 116.35, 116.57, 116.96, 118.64, 123.51, 126.22, 127.33, 130.36, 131.89, 134.83, 135.58, 137.19, 137.65, 144.04, 148.30, 152.62 (Ar-C), 157.21, 160.11, 161.07 (C=O), 161.51 (C=O coumarin), 167.77 (C=N). TOF-MS, *m*/*z*: 574 [M + H]^+^. Calcd for C_28_H_23_N_5_O_5_S_2_: elemental analysis: C, 58.63; H, 4.04; N, 12.21%; found C, 58.55; H, 3.96; N, 12.13.

**6,8-Dichloro-2-oxo-*N*-(2-oxo-2-{2-[(quinazolin-4-ylsulfanyl)acetyl]hydrazinyl}ethyl)-2*H*-chromene-3-carboxamide** (**9e**). Yield 0.51 g (81*%*); M.P. 280–282 °C. FT-IR (KBr) *ν_max_*, cm^–1^: 3256 (NH), 1724, 1666, 1648, 1612 (C=O), 1561 (C=N). ^1^H-NMR (400 MHz, DMSO-*d*_6_), δ, ppm: 11.74 (s, 1H, NH), 10.48 and 9.82 (s, 1H, NH), 9.14 (s, 1H, NH), 8.88 (s, 1H, H-4 coumarin), 8.13–8.06 (m, 2H, ArH), 7.86 (d, 1H, *J* = 8 Hz, ArH), 7.48–7.16 (m, 4H, ArH), 7.07 (s, 1H, ArH), 6.98–6.94 (m, 1H, ArH), 4.49 (d, 2H, *J* = 4 Hz, SCH_2_), 4.15 (s, 2H, NCH_2_), 4.06 (s, 2H, CH_2_). ^13^C-NMR (100 MHz, DMSO-*d*_6_), δ, ppm: 36.75 (CH_2_), 42.08 (NCH_2_), 42.28 (SCH_2_), 113.20, 117.16, 117.38, 118.76, 123.95, 123.98, 126.11, 131.31, 131.39, 136.85, 136.93, 142.20, 146.58, 150.44, 152.86 (Ar-C), 156.38, 161.67, 163.80 (C=O) 164.09 (C=O coumarin), 168.82 (C=N). LC-MS, *m/z*: 629 [M (Cl^35^)(Cl^37^)+H]^+^. Calcd for C_27_H_19_Cl_2_N_5_O_5_S_2_: elemental analysis: C, 51.60; H, 3.05; N, 11.14%; found C, 51.51; H, 2.98; N, 11.07.

**8-Methoxy-2-oxo-*N*-(2-oxo-2-{2-[(quinazolin-4-ylsulfanyl)acetyl]hydrazinyl}ethyl)-2*H*-chromene-3-carboxamide** (**9f**). Yield 0.45 g (76*%*); M.P. 284–285 °C. FT-IR (KBr) *ν_max_*, cm^–1^: 3291 (NH), 1717, 1706, 1666, 1609 (C=O), 1524 (C=N).^1^H-NMR (400 MHz, DMSO-*d*_6_), δ, ppm: 11.73 (s, 1H, NH), 10.48 and 9.82 (s, 1H, NH), 9.25 (s, 1H, NH), 8.88 (s, 1H, H-4 coumarin), 7.87–7.86 (m, 1H, ArH), 7.51–7.25 (m, 5H, ArH), 7.18–7.07 (m, 2H, ArH), 6.93–6.80 (m, 2H, ArH), 4.50 (s, 2H, SCH_2_), 4.22 and 4.21 (s, 2H, NCH_2_), 4.06 (s, 2H, CH_2_), 3.91 (s, 3H, OCH_3_). ^13^C-NMR (100 MHz, DMSO-*d*_6_), δ, ppm: 35.83 (CH_2_), 42.05 (NCH_2_), 43.49 (NCH_2_), 56.63 (OCH_3_), 116.48, 116.83, 118.88, 119.40, 121.69, 125.50, 125.58, 126.09, 127.24, 131.76, 137.14, 137.64, 143.72, 144.06, 146.68, 148.55, 157.20 (Ar-C), 160,59, 161.28 (C=O), 167.61 (C=N). Calcd for C_28_H_23_N_5_O_6_S_2_: elemental analysis: C, 57.03; H, 3.93; N, 11.88%; found C, 56.93; H, 3.85; N, 11.82.

### 3.2. Anticancer Activity

The synthesized compounds, HEK-293 human embryonic kidney cell line, and cancer cell lines (breast, MCF-7 and prostate, PC-3) were obtained from the American Type Cell Culture Collection (ATCC, USA). The cancer and healthy cell lines were grown in a 5% CO_2_ incubator, using Roswell Park Memorial Institute (RPMI) 1640 medium containing 10% fetal bovine serum and 1% penicillin. The activity of the synthesized compounds against the HEK-293 human healthy embryonic kidney cell line and cancer cell lines (MCF-7 breast, PC-3 prostate) are given in Table 1. Cells were seeded in 96-well plates with 104 cells per well. The synthesized compounds were applied to the cells at increasing concentrations for 34, 48 and 72 h. At the end of the incubation periods, 20 μL of MTT solution was added to each well and the plates were kept in a 37 °C incubator for another 4 h. After the MTT solution was withdrawn from the wells, 200 μL of dimethyl sulfoxide was added to each well and the optical densities in the wells were measured in a spectrophotometer (Tecan Infinite 200 PRO, Switzerland) at a wavelength of 570 nm. Drug doses that killed 50% of the cells (IC_50_) were calculated using the CalcuSyn program. Graphpad prism 5.0, a commercial statistical program, was used for the statistical analysis of the data obtained from the experimental and control groups. All experiments were performed in triplicate, and data are expressed as mean ± SD. Statistical significance was evaluated using Student’s t-test, comparing each compound with cisplatin. Exact *p*-values and 95% confidence intervals (95% CI) of IC_50_ values were calculated, and the results are provided in Supplementary Table S1.

### 3.3. Molecular Docking Studies

The binding affinities of compounds **9a**–**f** against three key cancer-related protein targets were evaluated using the Schrödinger’s Suite v. 2018-1 software package [49,51]. The selected targets, namely topoisomerase II (PDB ID: 3QX3), human ribonucleotide reductase (PDB ID: 5TUS), and kinesin-associated motor protein EG5 (PDB ID: 6G6Y), were downloaded from the Protein Data Bank (www.rcsb.org, 15 May 2025) [52,53,54,55]. Prior to docking, the protein structures underwent a thorough preparation process using the Protein Preparation Wizard module in Schrödinger’s Maestro [56]. This involved the removal of all water molecules located beyond a 5 Å radius from the binding site, followed by the addition of missing hydrogen atoms and side chains. The protonation states of the proteins were optimized for physiological pH (pH 7.4) using the PROPKA algorithm to ensure a more accurate representation of the physiological environment. The synthesized ligands were constructed in the Maestro Build Panel and prepared for docking using the LigPrep module with default parameters to generate all relevant protonation and tautomeric states [57]. The binding sites for each protein were precisely defined by centering the grid box around the co-crystallized ligands present in the downloaded structures. A crucial aspect of this study was the use of the Induced Fit Docking (IFD) protocol, which accounts for the conformational changes in both the ligand and the receptor upon binding and was used as the docking method. This protocol was executed in the extra precision (XP) mode to enhance the accuracy of the docking pose predictions and scoring. The final docking scores and detailed interaction profiles, including hydrogen bonds, hydrophobic contacts, and π-stacking interactions, were meticulously analyzed to identify compounds with the highest predicted activity, providing a robust foundation for explaining experimental data. The binding affinities were comparatively evaluated by utilizing reference drugs for each target: etoposide for topoisomerase II (PDB ID: 3QX3), gemcitabine for human ribonucleotide reductase (PDB ID: 5TUS), and ispinesib for the kinesin-associated motor protein EG5 (PDB ID: 6G6Y).

To verify the reliability of the computational methodology, a rigorous re-docking procedure was performed for each protein target. This involved docking the co-crystallized ligand back into the relevant binding pocket. The results demonstrated a high degree of accuracy, with Root Mean Square Deviation (RMSD) values of 1.96 Å for 6G6Y, 1.68 Å for 3QX3, and 1.83 Å for 5TUS. The fact that all calculated RMSD values are below the widely accepted threshold of 2.0 Å provides evidence that the docking protocol used and the defined binding site are reliable and can accurately reproduce known binding positions.

## 4. Conclusions

In conclusion, we designed and synthesized six novel glycine-conjugated hybrid compounds containing coumarin, thiophene and quinazoline moieties, as potential prostate and breast cancer agents. Our findings demonstrated that compounds which have substitutions on the coumarin ring have more activity against both prostate and breast cancer cell lines than the compound with no substitution (except for the chlorine substitution at the 6th position of the coumarin ring). Among the synthesized compounds, **9f** (which has a methoxy group on 8th position of the coumarin ring) has the highest activity against both prostate and breast cancer cell lines. The activity results of the compounds showed that substitution at the 8th position on the coumarin ring enhanced the anticancer activity, as seen in compound **9f** and **9e**. When the selectivity index values of the synthesized compounds against cancer cell lines were compared, substitution on the coumarin ring resulted in decreasing of the selectivity index value. More substitution at the 8th position on the coumarin ring decreased the selectivity index value, as can be seen from the selectivity index value of **9e** and **9f**.

A direct correlation between the in silico docking results and the in vitro cytotoxicity was observed, particularly for compound **9f**. This compound consistently yielded the most favorable docking scores against EG5, Human Ribonucleotide Reductase, and Topoisomerase II, which aligned with its highest cytotoxic activity in the experimental assays. This suggested that the strong predicted binding affinities computationally determined for compound **9f** were translated into substantial cellular toxicity. The consistently strong predicted binding affinities of compound **9f** across EG5, Human Ribonucleotide Reductase, and Topoisomerase II strongly suggested its potential as a promising lead compound. These computational findings indicated that compound **9f** possessed the capacity of interacting favorably with multiple proteins critically involved in cancer cell growth and survival. Such multi-target activity was considered a valuable attribute in the development of novel anticancer agents. However, these predictions necessitate rigorous experimental validation through in vitro and in vivo studies to confirm the actual binding affinities, potency, and selectivity, thereby advancing the potential for compound **9f** as a therapeutic candidate in cancer treatment.

The findings obtained from this study suggest that glycine-conjugated quinazoline hybrids containing a coumarin ring could possess potent anticancer properties. Another important point to consider is that changing the substitution on the coumarin ring is one of the important factors affecting the anticancer activity. Consequently, these hybrid compounds could be considered further in the process of drug discovery and design, with the potential to develop new medications to treat cancer.

## Figures and Tables

**Table 1:** The anticancer activity and selectivity index (SI) value of synthesized compounds against prostate cancer (PC-3), breast cancer (MCF-7), and human embryonic kidney (HEK-293) cell lines. IC_50_ values represent the mean ± SD of three independent experiments. SI values were calculated relative to HEK-293 cells as the non-cancerous control line.

IC_50_ (Cytotoxicity 50: µg/L); SI (Selectivity Index)					
Comp. no.	Prostate Cancer (PC-3)	SI	Breast Cancer (MCF-7)	SI	Human Embryonic Kidney (HEK-293)
9a	61.3 ± 2.6	3.22	80.7 ± 1.1	2.45	197.6 ± 1.5
9b	85.5 ± 1.1	1.57	104.9 ± 0.9	1.29	134.6 ± 1.6
9c	47.7 ± 0.3	2.51	71.2 ± 0.2	1.68	119.9 ± 2.1
9d	43.0 ± 0.9	2.14	74.3 ± 1.4	1.24	91.9 ± 1.4
9e	34.6 ± 2.2	1.98	46.1 ± 1.4	1.49	68.8 ± 0.8
9f	14.7 ± 1.4	0.59	16.5 ± 1.2	0.53	8.7 ± 2.4
Cisplatin	24.1 ± 0.4	0.85	22.1 ± 1.0	0.93	20.6 ± 2.4

**Table 2:** Docking scores of newly synthesized compounds for selected targets.

	6G6Y	5TUS	3QX3
**9a**	−11.029	−7.99	−9.956
**9b**	−11.084	−7.133	−8.622
**9c**	−11.256	−6.627	−10.874
**9d**	−11.272	−8.368	−9.902
**9e**	−10.883	−7.603	−8.021
**9f**	−11.881	−9.088	−12.694
Ref. Drug *	−12.618	−8.524	−9.423

* Reference Drug: Ispinesib for 6G6Y, Gemcitabine for 5TUS, Etoposite for 3QX3. 6G6Y: Eg5-inhibitor complex; 5TUS: Potent competitive inhibition of human ribonucleotide reductase by a novel non-nucleoside small molecule; 3QX3: Human topoisomerase II beta in complex with DNA and etoposide.

**Table 3:** Correlation analysis between docking scores and IC_50_ values for synthesized compounds (**9a**–**f**).

Protein Target	Pearson r (PC-3)	*p*-Value	Pearson r (MCF-7)	*p*-Value
Eg5	0.60	0.20	0.63	0.18
RNR	0.62	0.19	0.63	0.18
Topo II	0.58	0.23	0.57	0.24

## Data Availability

Data is contained within the article and Supplementary Materials.

## Supplementary Materials

The following supporting information can be downloaded at https://www.mdpi.com/article/10.3390/ph18111627/s1, Figure S1: IR spectra of compound 2; Figure S2:1H NMR spectra of compound 2 (DMSO-d6); Figure S3:13C NMR (APT) spectra of compound 2 (DMSO-d6); Figure S4: IR spectra of compound 3; Figure S5: ^1^H NMR spectra of compound 3 (DMSO-d_6_); Figure S6: ^13^C NMR (APT) spectra of compound 3 (DMSO-d_6_); Figure S7: IR spectra of compound 4; Figure S8: ^1^H NMR spectra of compound 4 (DMSO-d_6_); Figure S9: ^13^C NMR (APT) spectra of compound 4 (DMSO-d_6_); Figure S10: IR spectra of compound 7b; Figure S11: ^1^H NMR spectra of compound 7b (DMSO-d_6_); Figure S12: ^13^C NMR (APT) spectra of compound 7b (DMSO-d_6_); Figure S13: IR spectra of compound 7c; Figure S14: ^1^H NMR spectra of compound 7c (DMSO-d_6_); Figure S15: ^13^C NMR (APT) spectra of compound 7c (DMSO-d_6_); Figure S16: IR spectra of compound 7d; Figure S17: ^1^H NMR spectra of compound 7d (DMSO-d_6_); Figure S18: ^13^C NMR (APT) spectra of compound 7d (DMSO-d_6_); Figure S19: IR spectra of compound 7e; Figure S20: ^1^H NMR spectra of compound 7e (DMSO-d_6_); Figure S21: ^13^C NMR (APT) spectra of compound 7e (DMSO-d_6_); Figure S22: IR spectra of compound 7f; Figure S23: ^1^H NMR spectra of compound 7f (DMSO-d_6_); Figure S24: ^13^C NMR (APT) spectra of compound 7f (DMSO-d_6_); Figure S25: IR spectra of compound 8b; Figure S26: ^1^H NMR spectra of compound 8b (DMSO-d_6_); Figure S27: ^13^C NMR (APT) spectra of compound 8b (DMSO-d_6_); Figure S28: IR spectra of compound 8c; Figure S29: ^1^H NMR spectra of compound 8c (DMSO-d_6_); Figure S30: ^13^C NMR (APT) spectra of compound 8c (DMSO-d_6_); Figure S31: IR spectra of compound 8d; Figure S32: ^1^H NMR spectra of compound 8d (DMSO-d_6_); Figure S33: ^13^C NMR spectra of compound 8d (DMSO-d_6_); Figure S34: IR spectra of compound 8e; Figure S35: ^1^H NMR spectra of compound 8e (DMSO-d_6_); Figure S36: ^13^C NMR (APT) spectra of compound 8e (DMSO-d_6_); Figure S37: IR spectra of compound 8f; Figure S38: ^1^H NMR spectra of compound 8f (DMSO-d_6_); Figure S39: ^13^C NMR (APT) spectra of compound 8f (DMSO-d_6_); Figure S40: IR spectra of compound 9a; Figure S41: ^1^H NMR spectra of compound 9a (DMSO-d_6_); Figure S42: ^13^C NMR (APT) spectra of compound 9a (DMSO-d_6_); Figure S43: LC MS spectra of compound 9a; Figure S44: IR spectra of compound 9b; Figure S45: ^1^H NMR spectra of compound 9b (DMSO-d_6_); Figure S46: ^13^C NMR (APT) spectra of compound 9b (DMSO-d_6_); Figure S47: LC MS spectra of compound 9b; Figure S48: IR spectra of compound 9c; Figure S49: ^1^H NMR spectra of compound 9c (DMSO-d_6_); Figure S50: ^13^C NMR (APT) spectra of compound 9c (DMSO-d_6_); Figure S51: LC MS spectra of compound 9c; Figure S52: IR spectra of compound 9d; Figure S53: ^1^H NMR spectra of compound 9d (DMSO-d_6_); Figure S54: ^13^C NMR (APT) spectra of compound 9d (DMSO-d_6_); Figure S55: LC MS spectra of compound 9d; Figure S56: IR spectra of compound 9e; Figure S57: ^1^H NMR spectra of compound 9e (DMSO-d_6_); Figure S58: ^13^C NMR (APT) spectra of compound 9e (DMSO-d_6_); Figure S59: LC MS spectra of compound 9e; Figure S60: IR spectra of compound 9f; Figure S61: ^1^H NMR spectra of compound 9f (DMSO-d_6_); Figure S62: ^13^C NMR (APT) spectra of compound 9f (DMSO-d_6_).
