# Supplementary material for: Synthesis and Anticancer Evaluation of Some Glycine Conjugated Hybrid Compounds Containing Coumarin, Thiophene and Quinazoline Moieties
Source: Pharmaceuticals (Basel). 2025 Oct 28;18(11):1627. doi: 10.3390/ph18111627 (PMC12654990; doi:10.3390/ph18111627)
Supplement: Supplementary file 1 [file pharmaceuticals-18-01627-s001.zip › suplemantary new.pdf]

Suplementary Material

Synthesis and Anticancer Evaluation of Some Glycine Conjugated Hybrid Compounds Containing Coumarin, Thiophene and Quinazoline Moieties

Nedime Çalışkan[1], Emre Menteşe[1], Fatih Yılmaz[2*], Süleyman İlhan[3], Mustafa Emirik[1]

[1]Department of Chemistry, Faculty of Art and Sciences, Recep Tayyip Erdogan University, 53100, Rize, Turkey

[2]Vocational School of Technical Sciences, Department of Chemistry and Chemical Process Technology, Recep Tayyip Erdogan University, 53100, Rize, Turkey

[3]Department of Biology, Faculty of Engineering and Natural Sciences, Manisa Celal Bayar University, 45140 Manisa, Türkiye

**fyilmaz@erdogan.edu.tr*

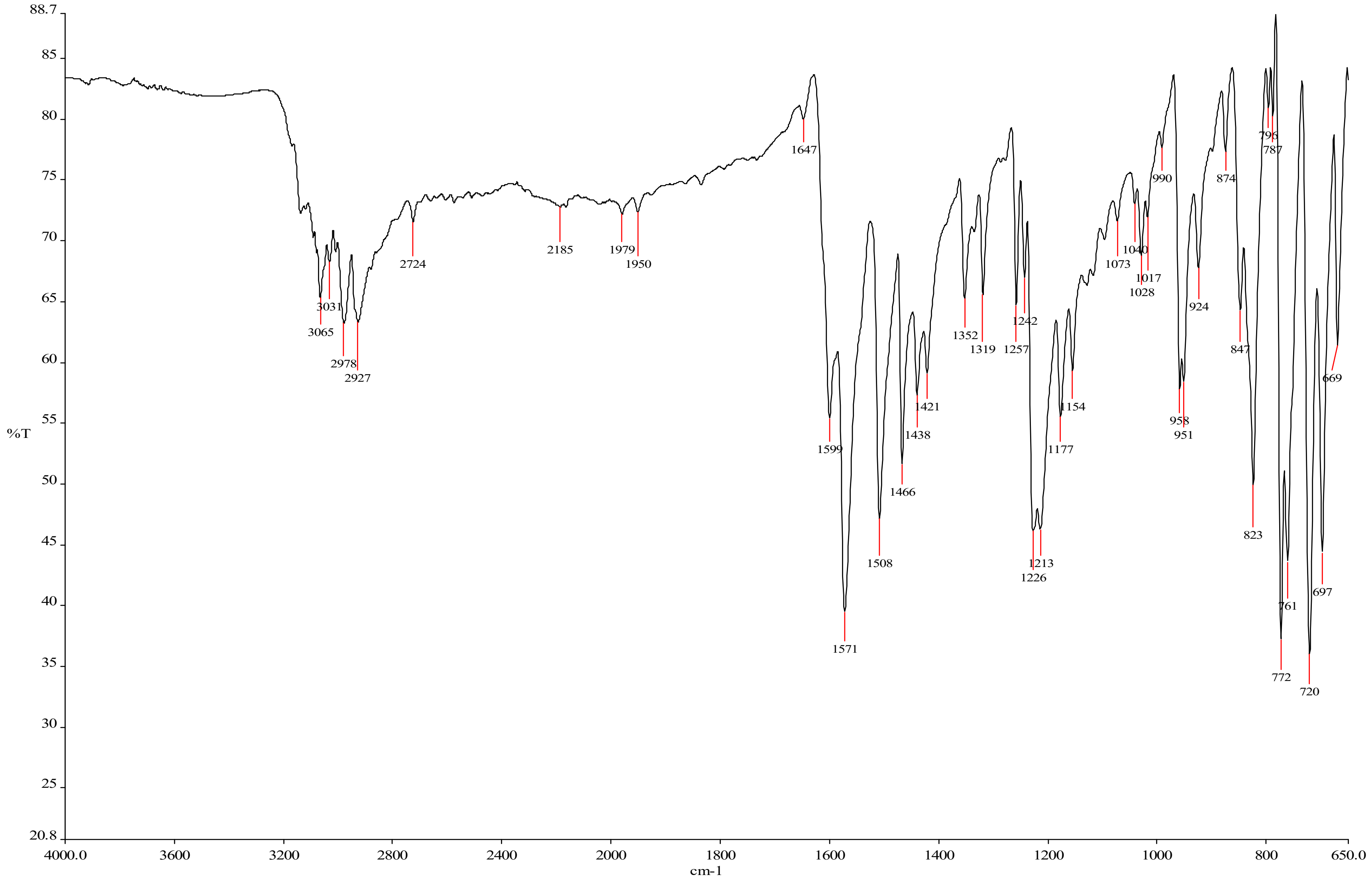

Figure S1: IR spectra of compound **2**

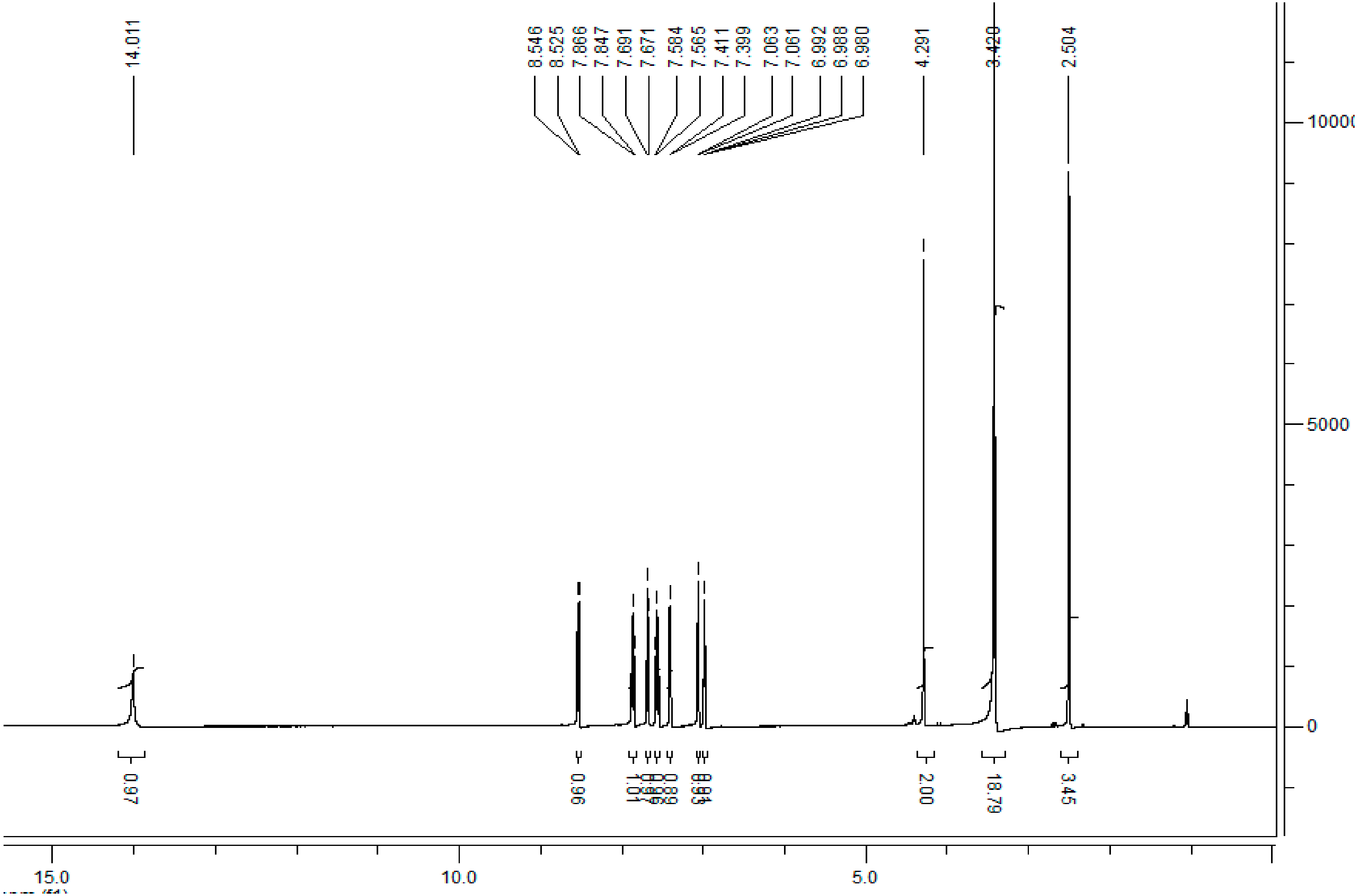

Figure S2:^{1}H NMR spectra of compound **2** (DMSO-d_6)

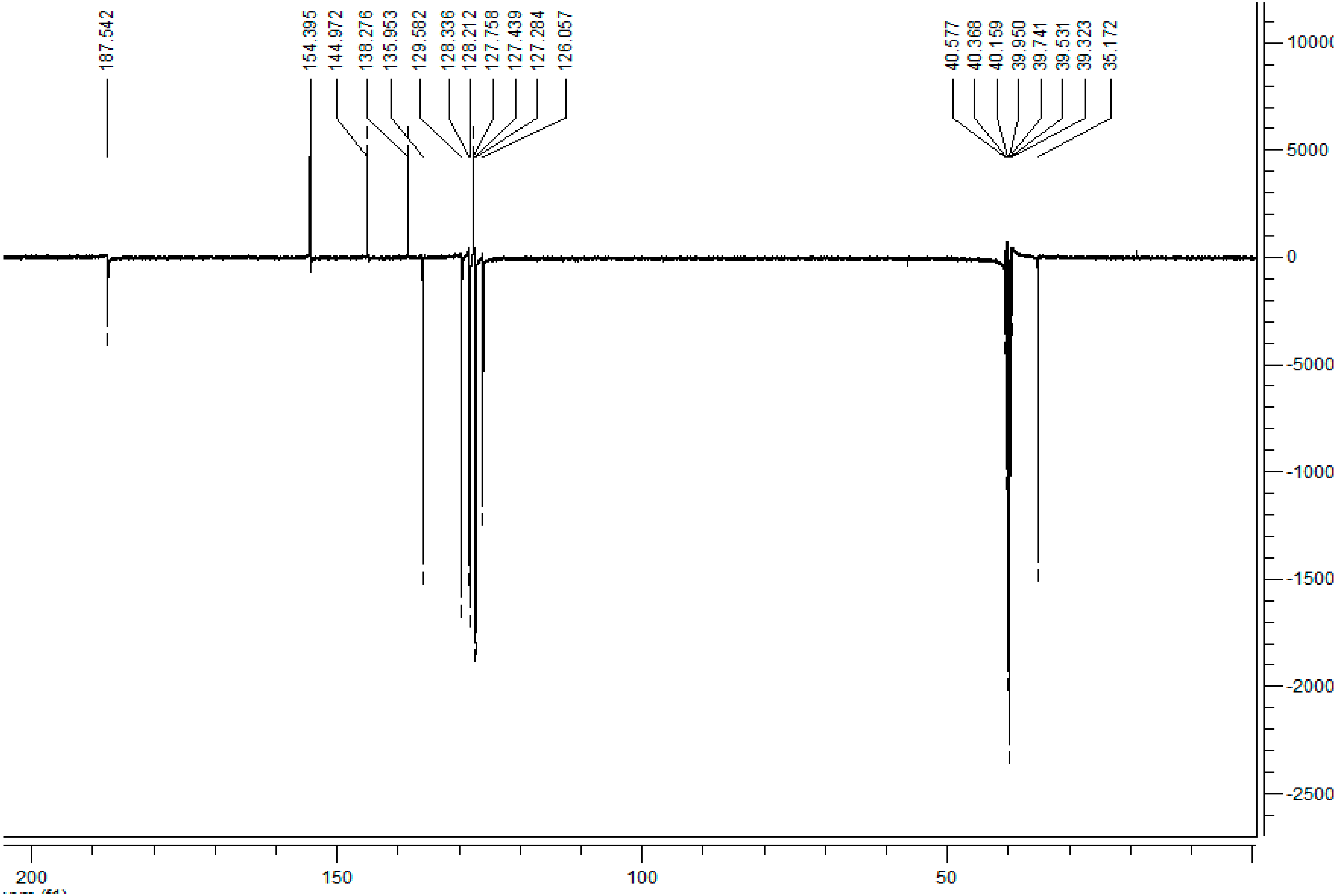

Figure S3:^{13}C NMR (APT) spectra of compound **2** (DMSO-d_6)

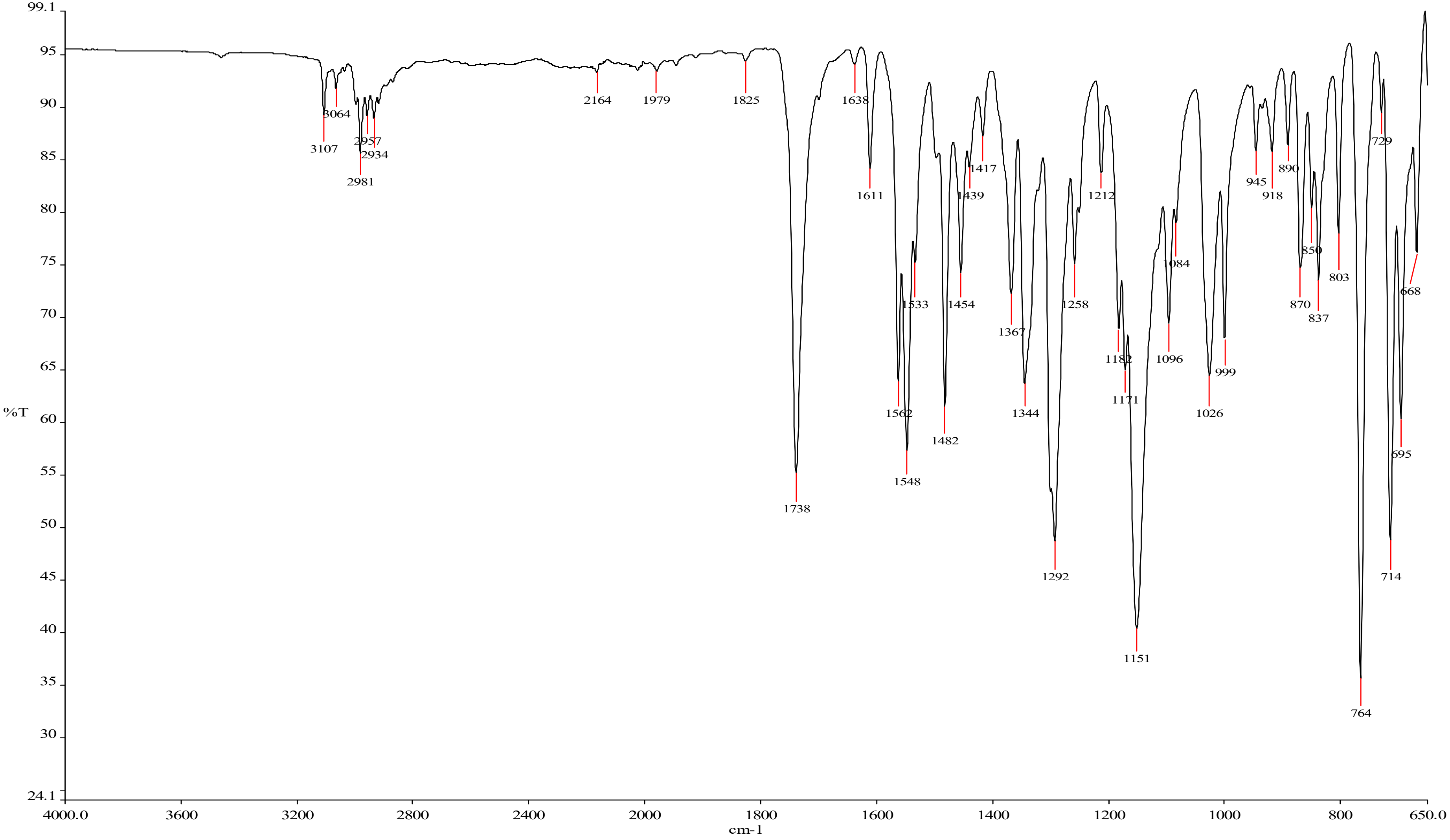

Figure S4: IR spectra of compound **3**

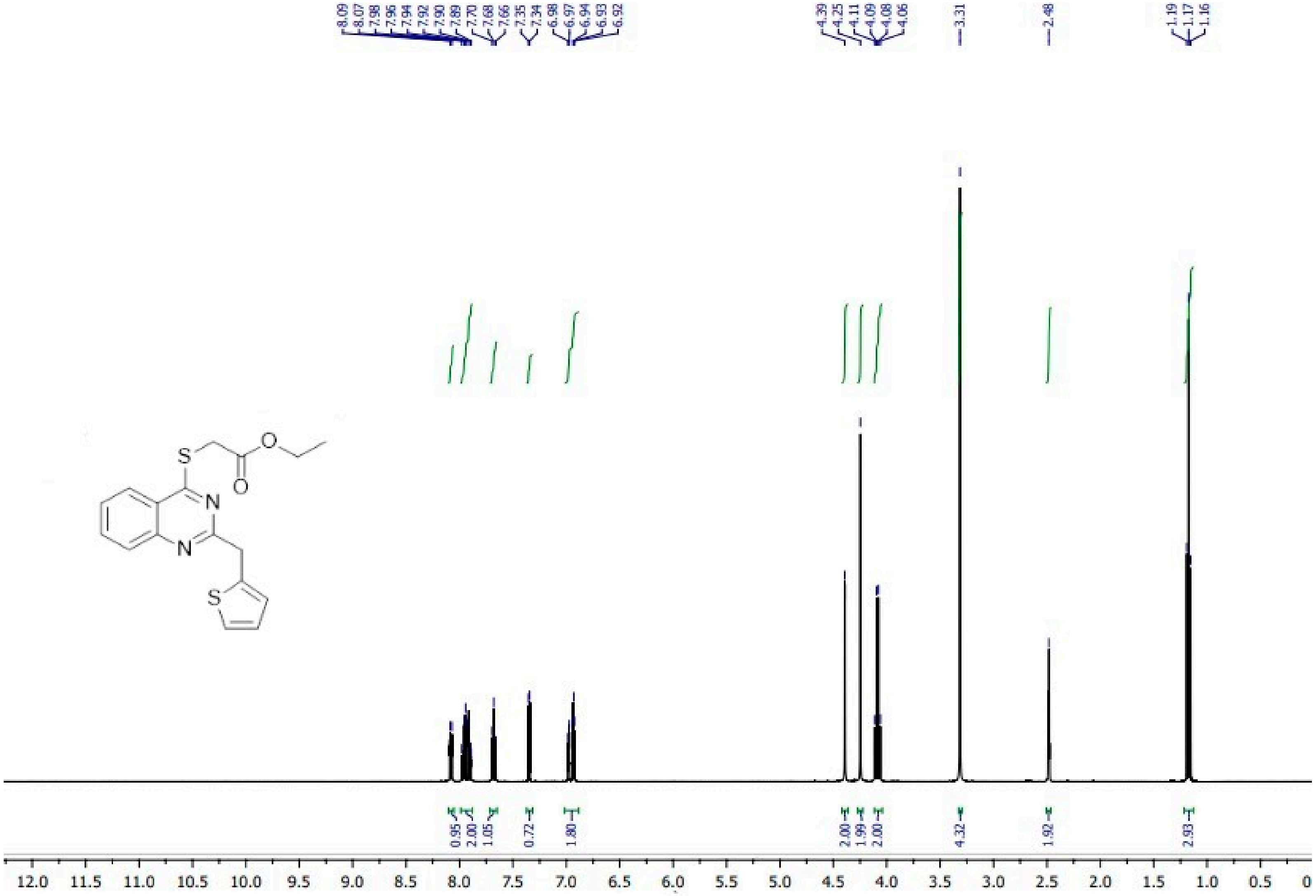

Figure S5: ^{1}H NMR spectra of compound **3** (DMSO-d_6)

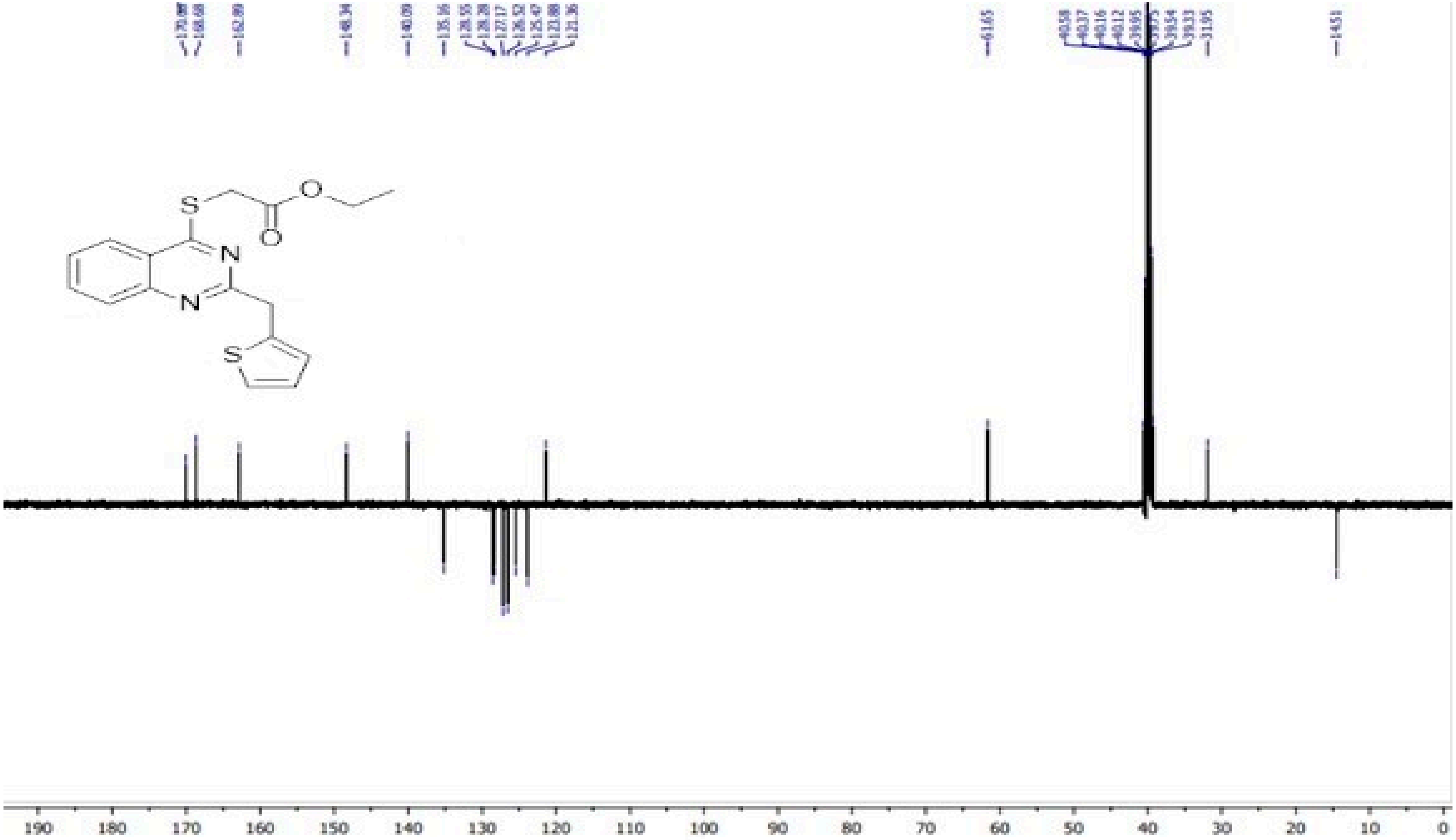

Figure S6: ^{13}C NMR (APT) spectra of compound **3** (DMSO-d_6)

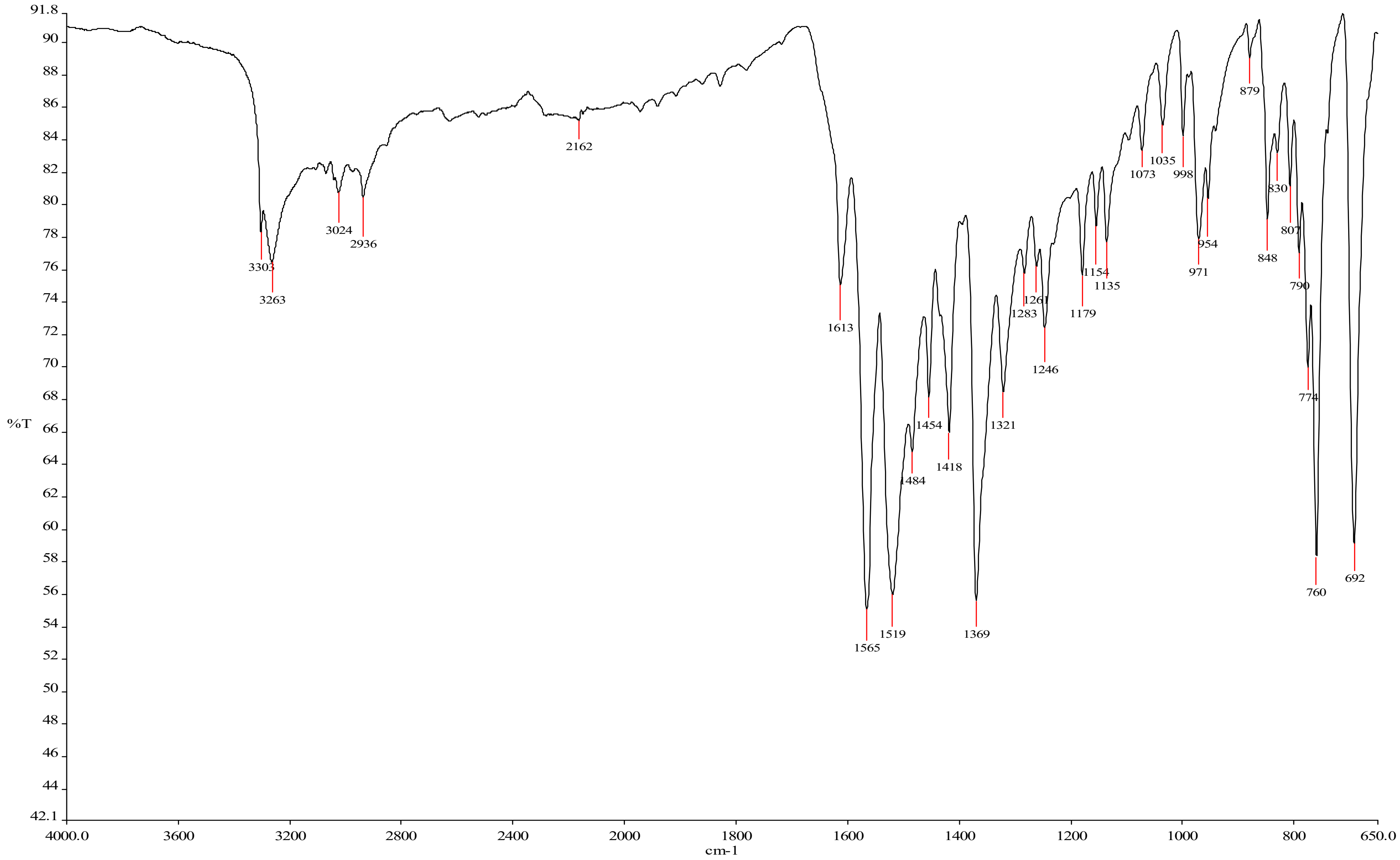

Figure S7: IR spectra of compound **4**

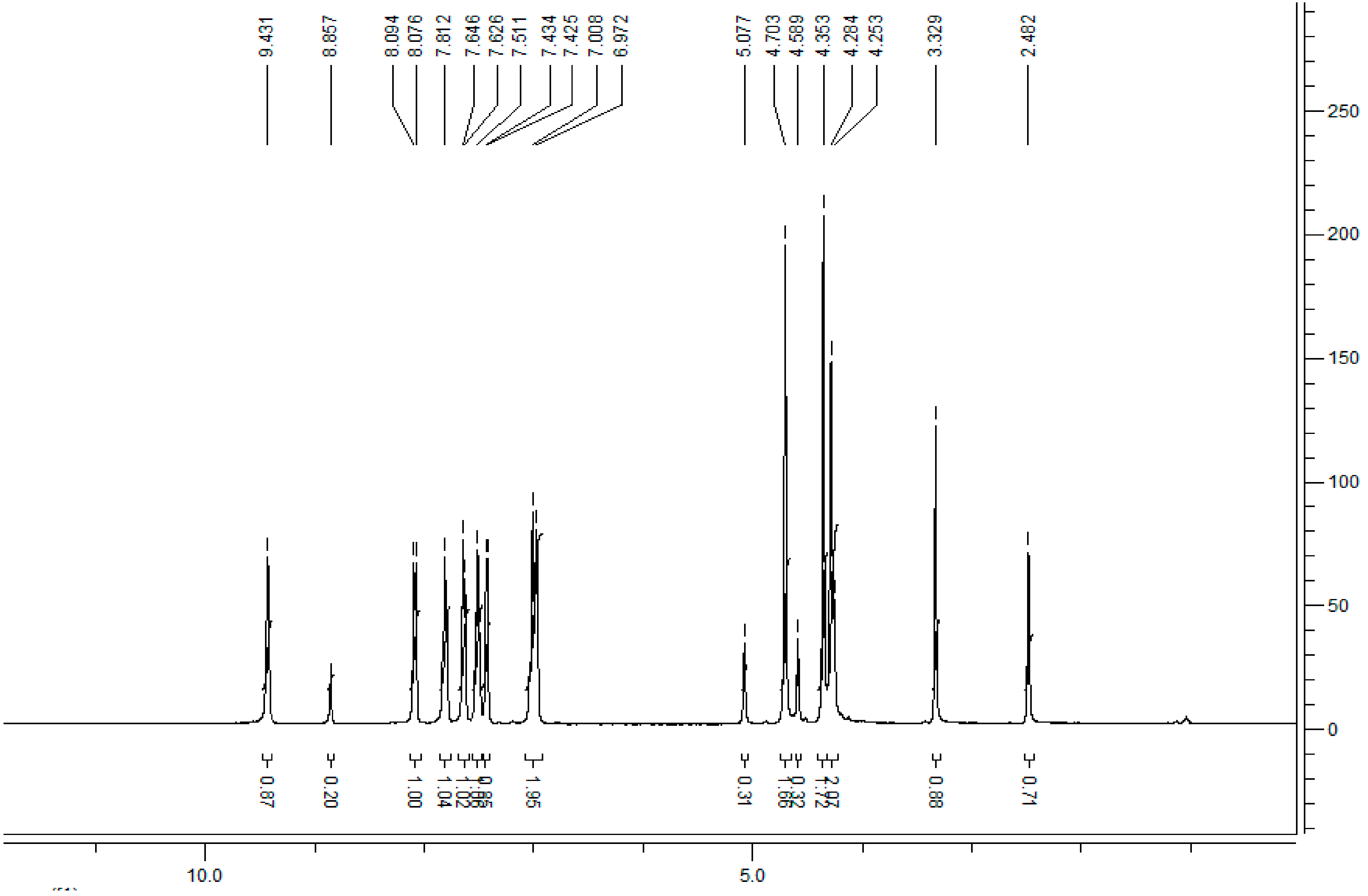

Figure S8: ^{1}H NMR spectra of compound **4** (DMSO-d_6)

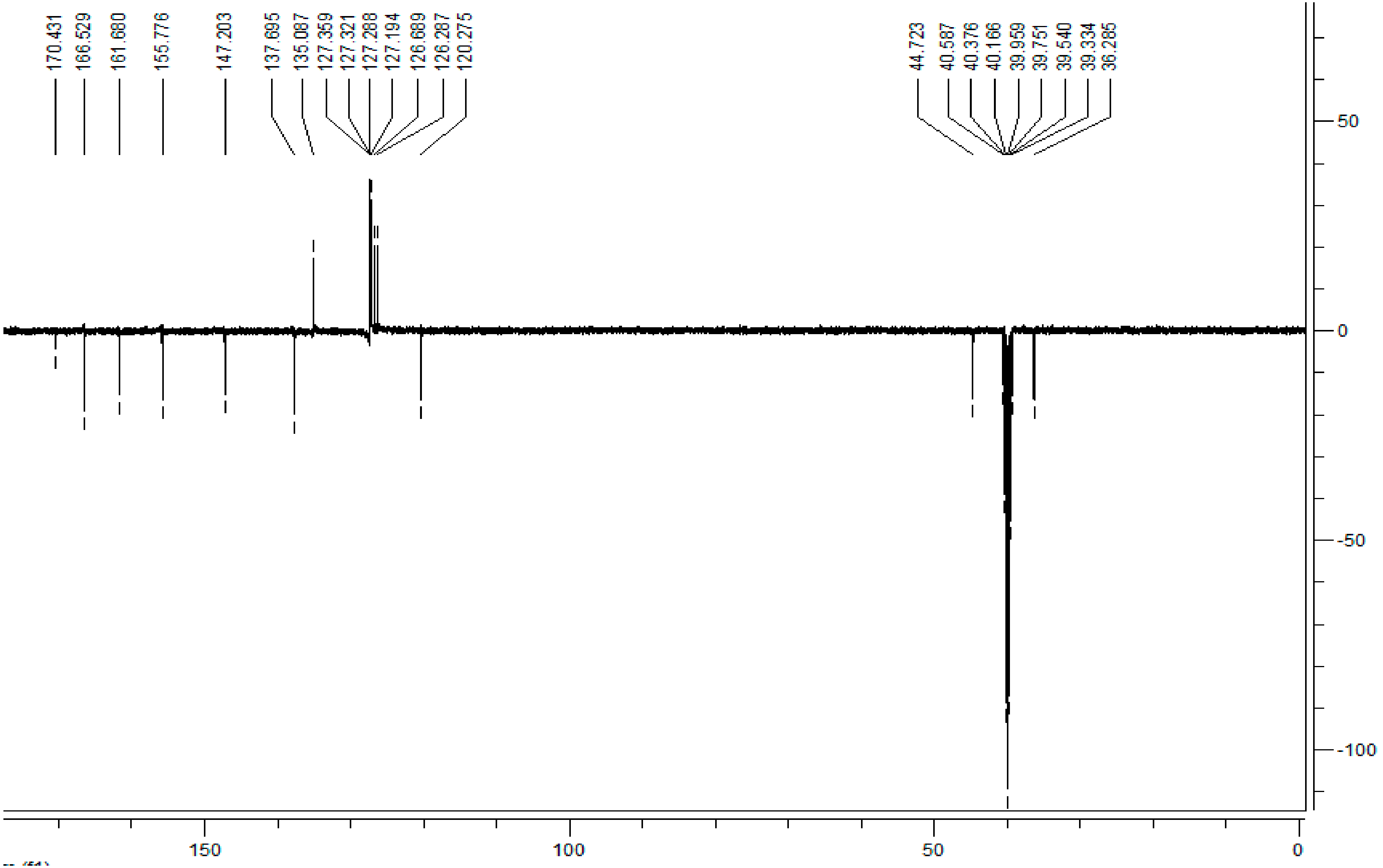

Figure S9: ^{13}C NMR (APT) spectra of compound **4** (DMSO-d_6)

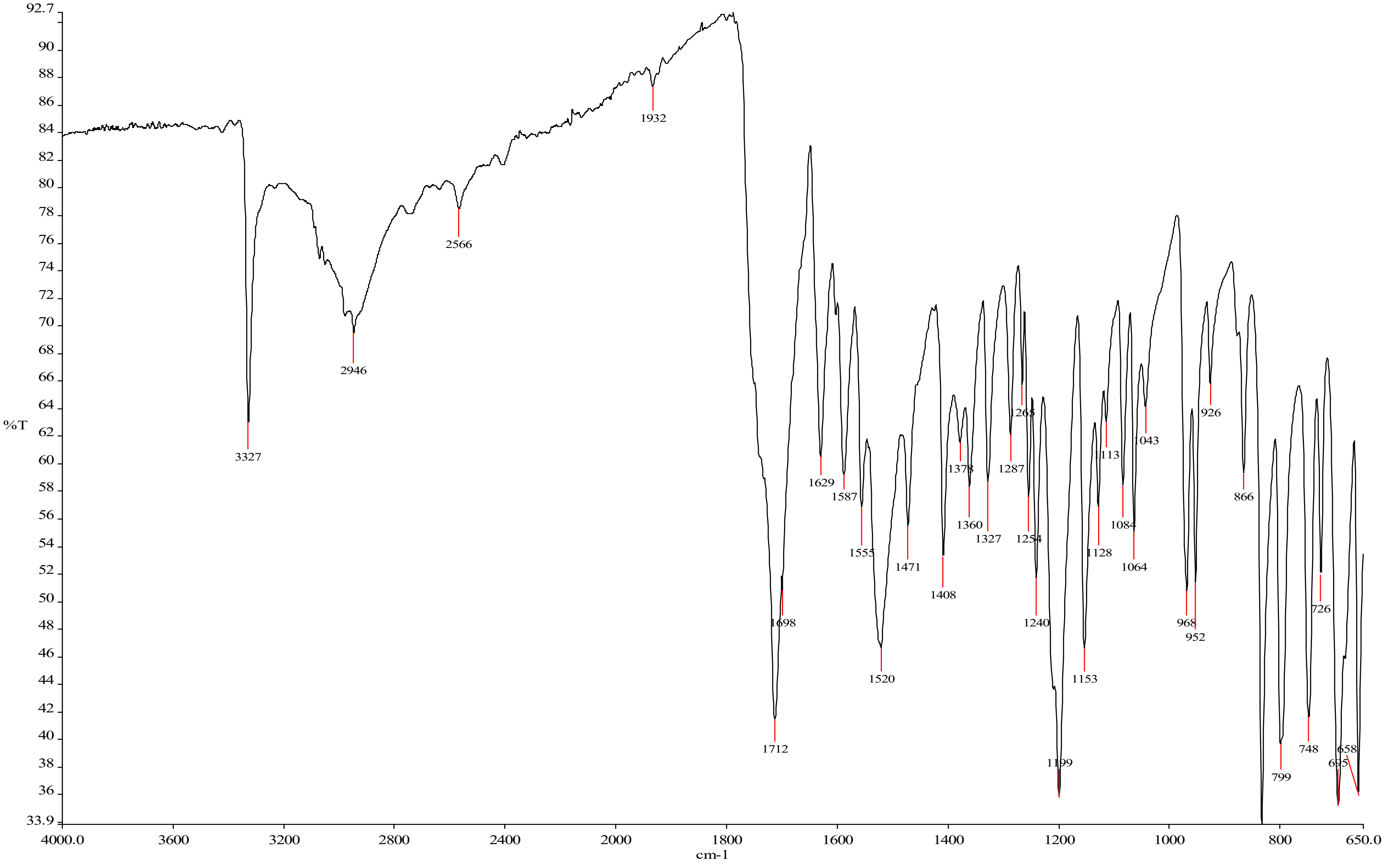

Figure S10: IR spectra of compound **7b**

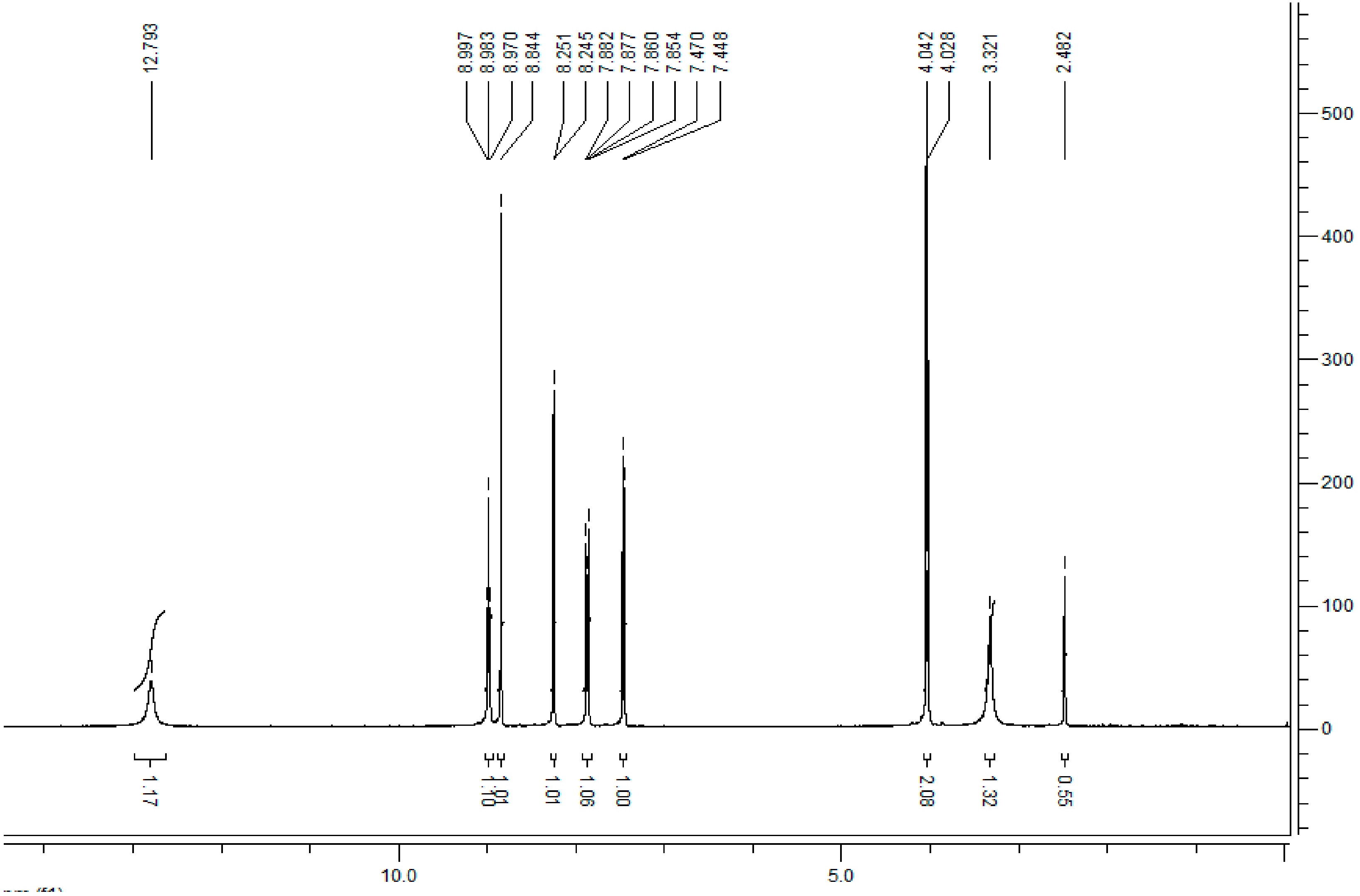

Figure S11: ^{1}H NMR spectra of compound **7b** (DMSO-d_6)

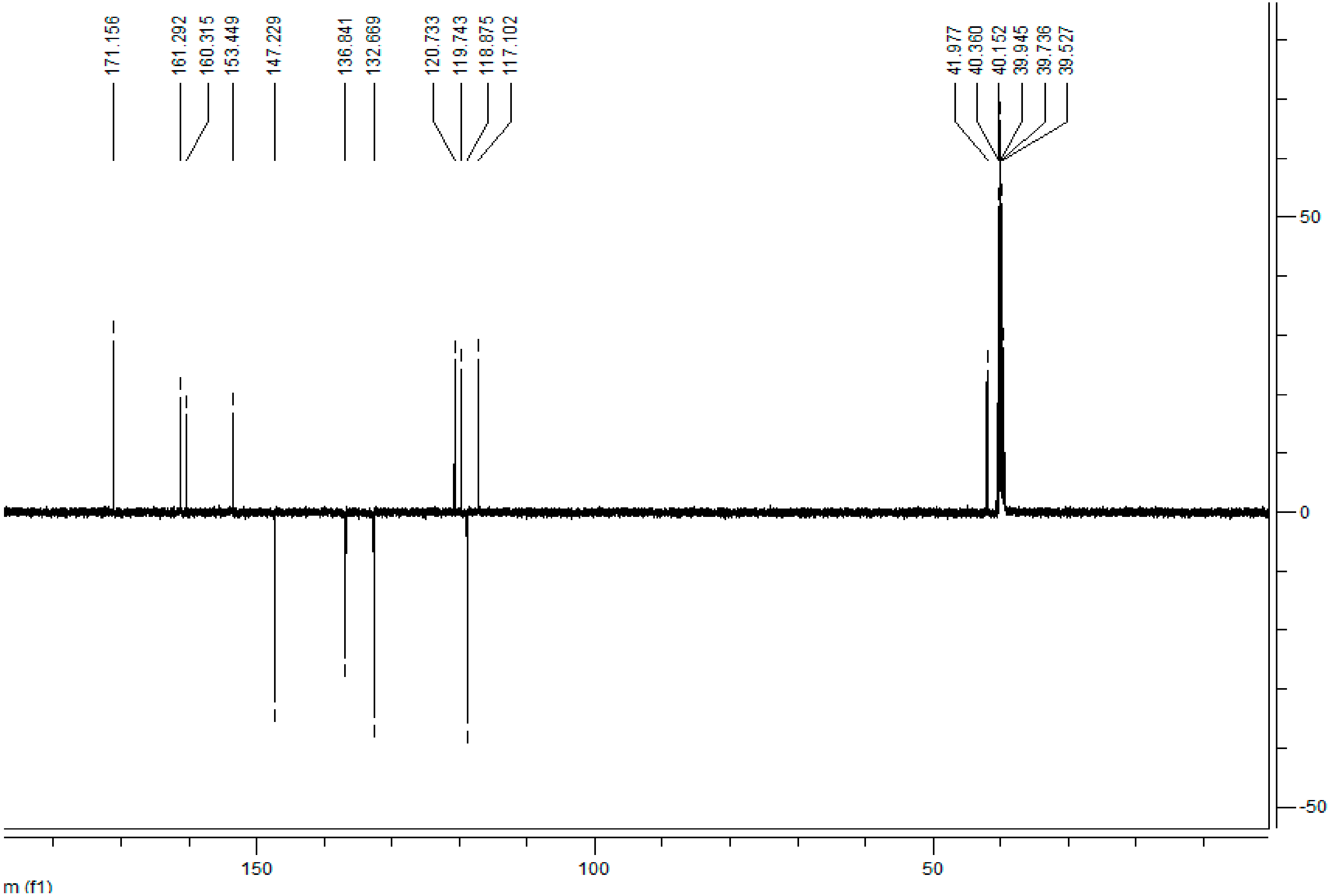

Figure S12: ^{13}C NMR (APT) spectra of compound **7b** (DMSO-d_6)

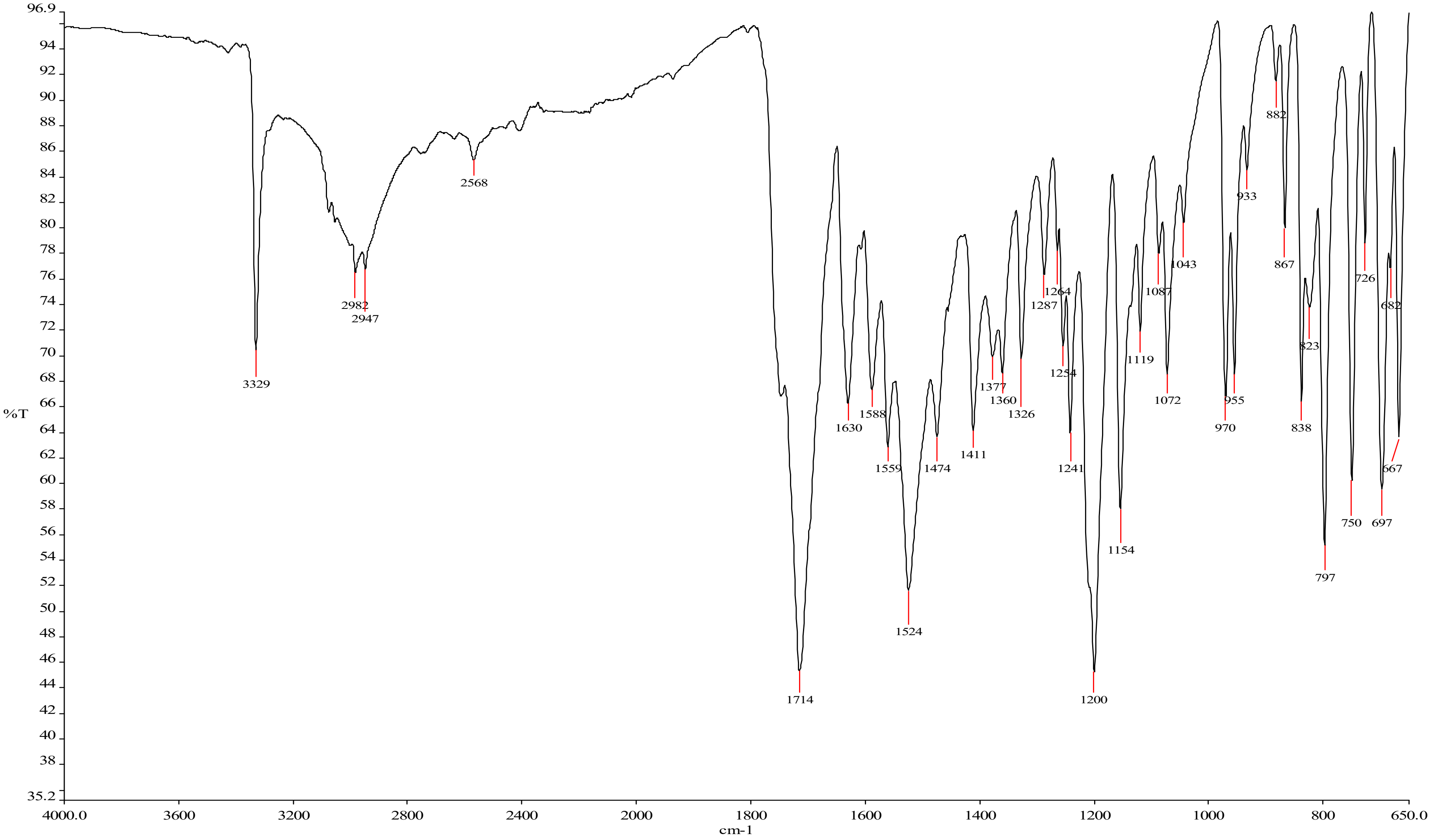

Figure S13: IR spectra of compound **7c**

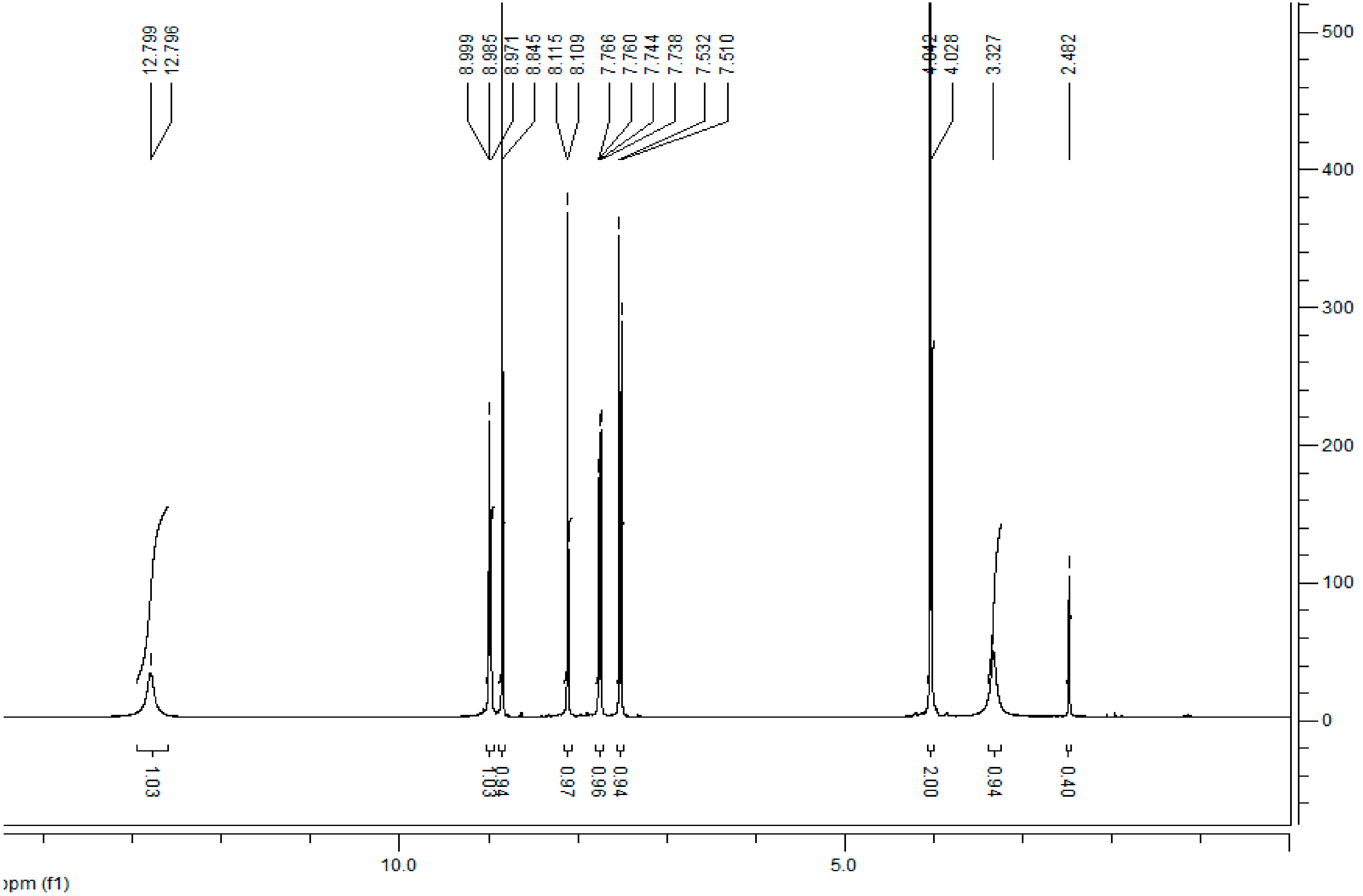

Figure S14: ^{1}H NMR spectra of compound **7c** (DMSO-d_6)

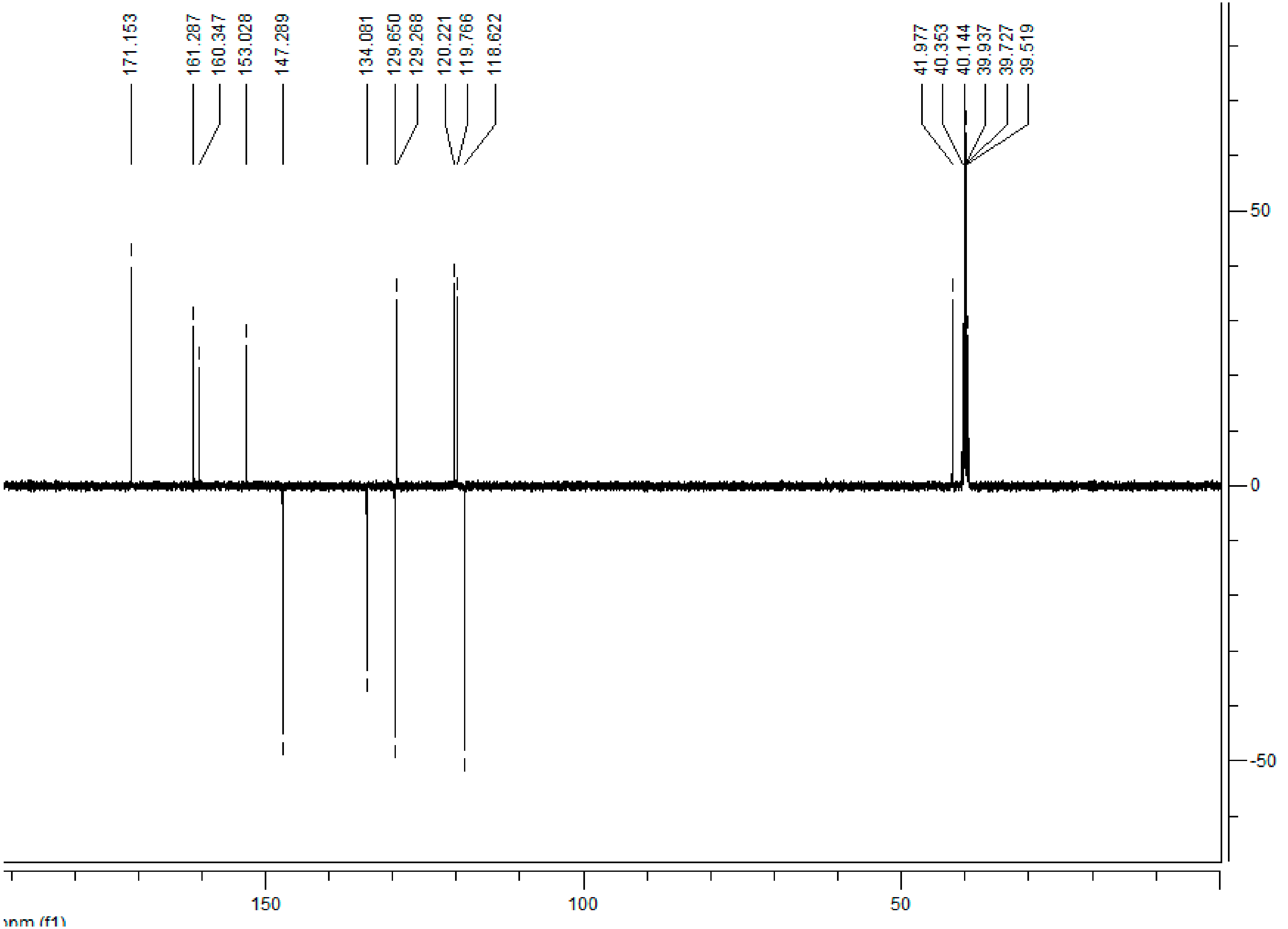

Figure S15: ^{13}C NMR (APT) spectra of compound **7c** (DMSO-d_6)

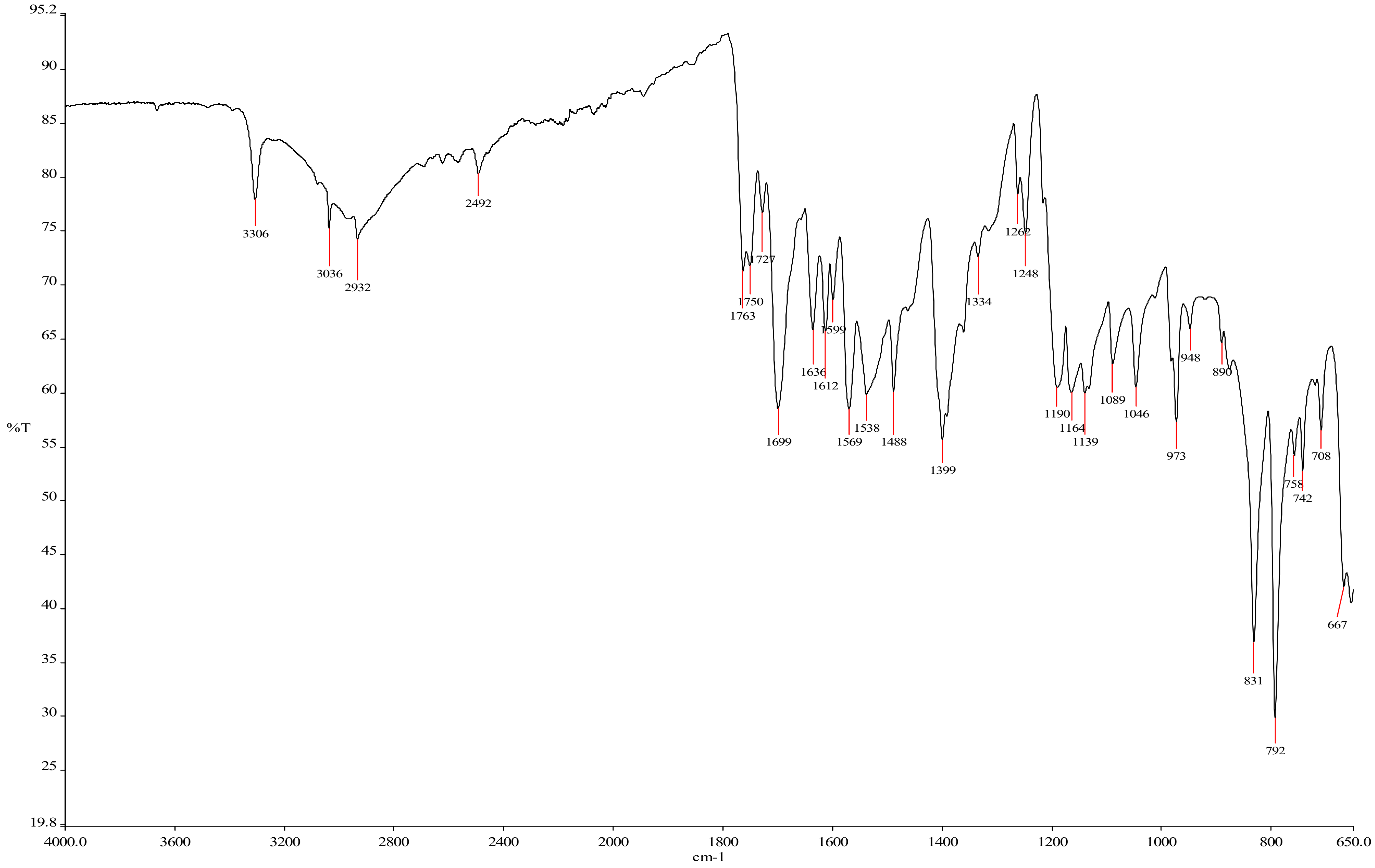

Figure S16: IR spectra of compound **7d**

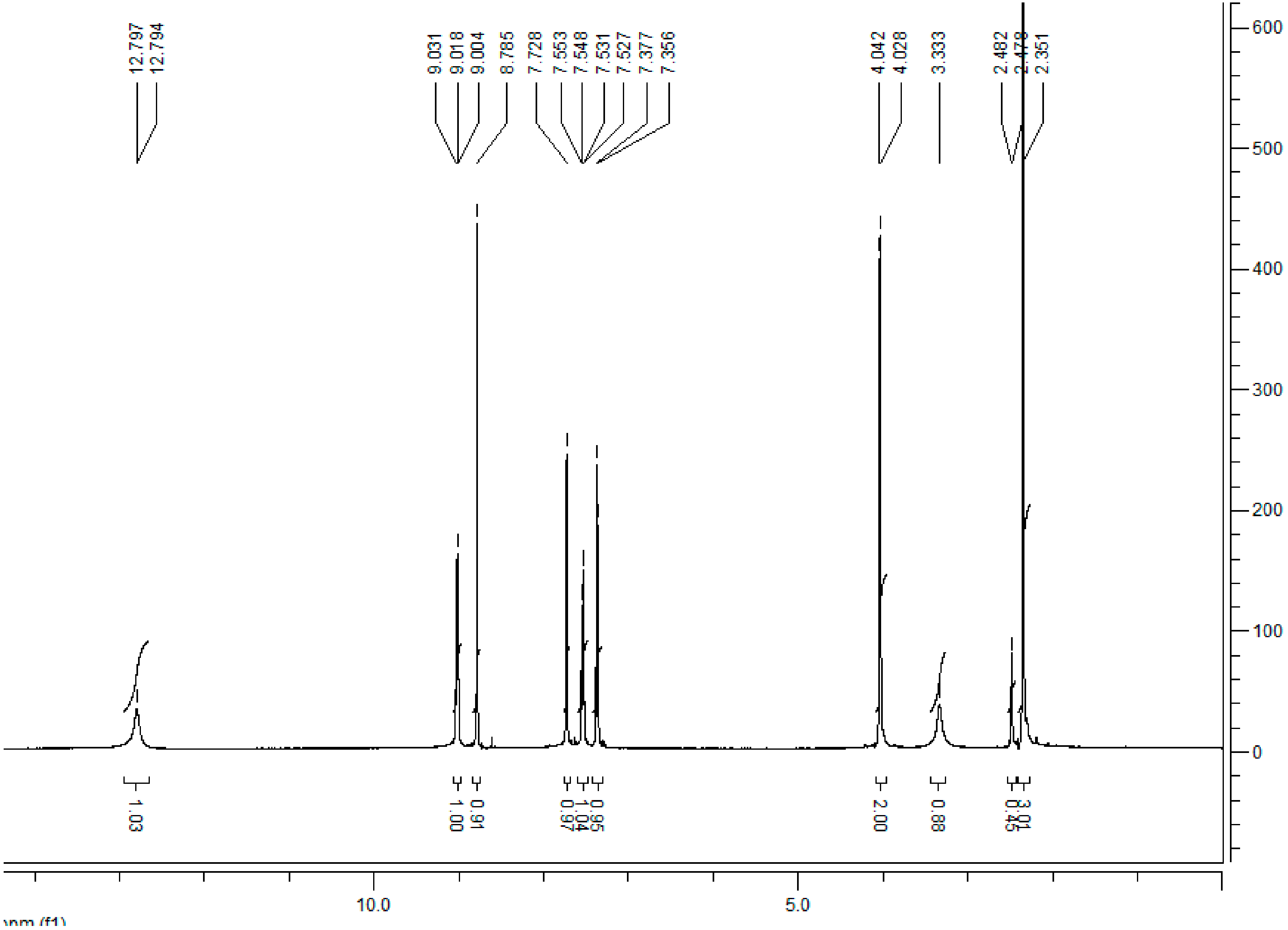

Figure S17: ^{1}H NMR spectra of compound **7d** (DMSO-d_6)

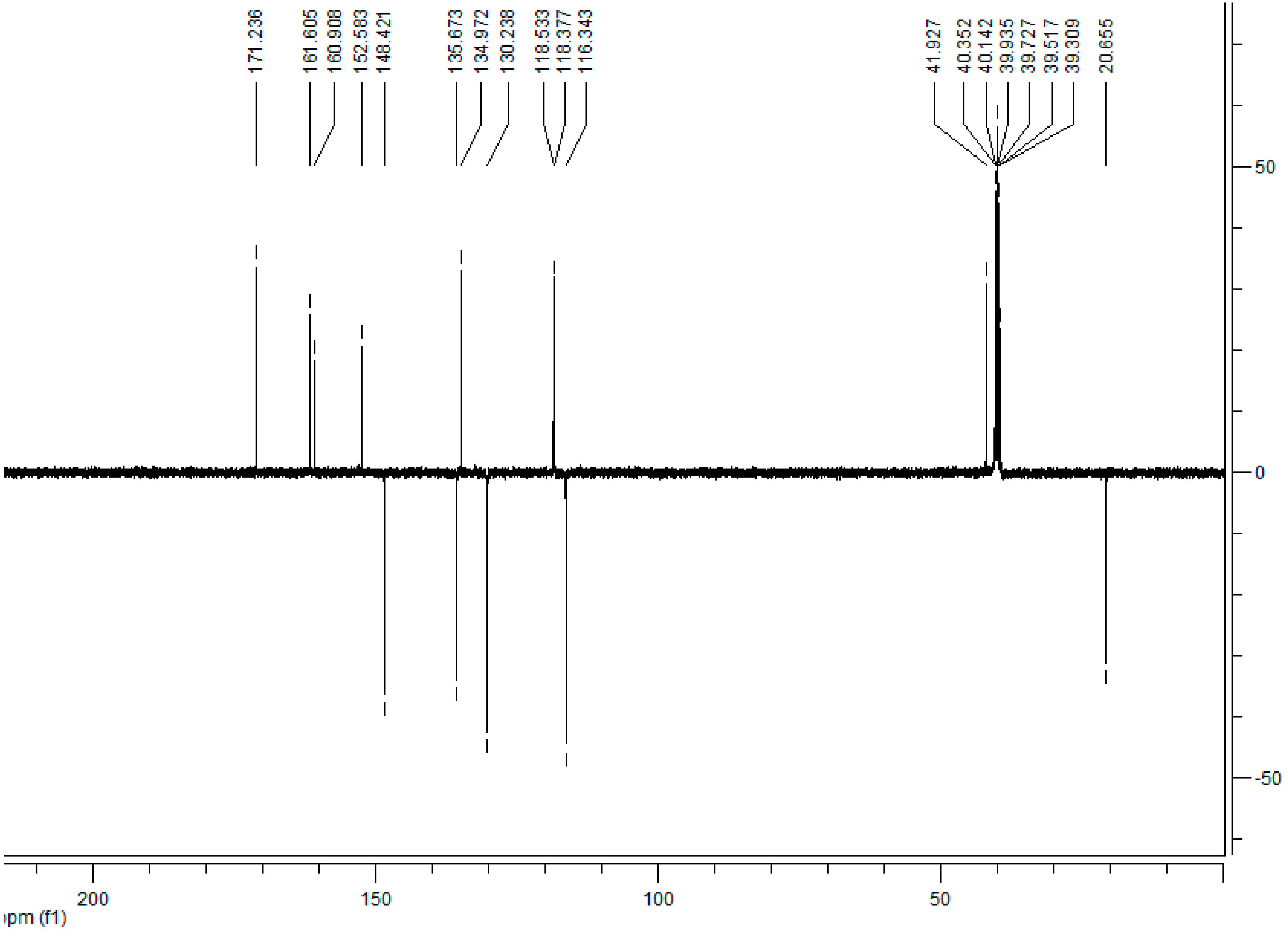

Figure S18: ^{13}C NMR (APT) spectra of compound **7d** (DMSO-d_6)

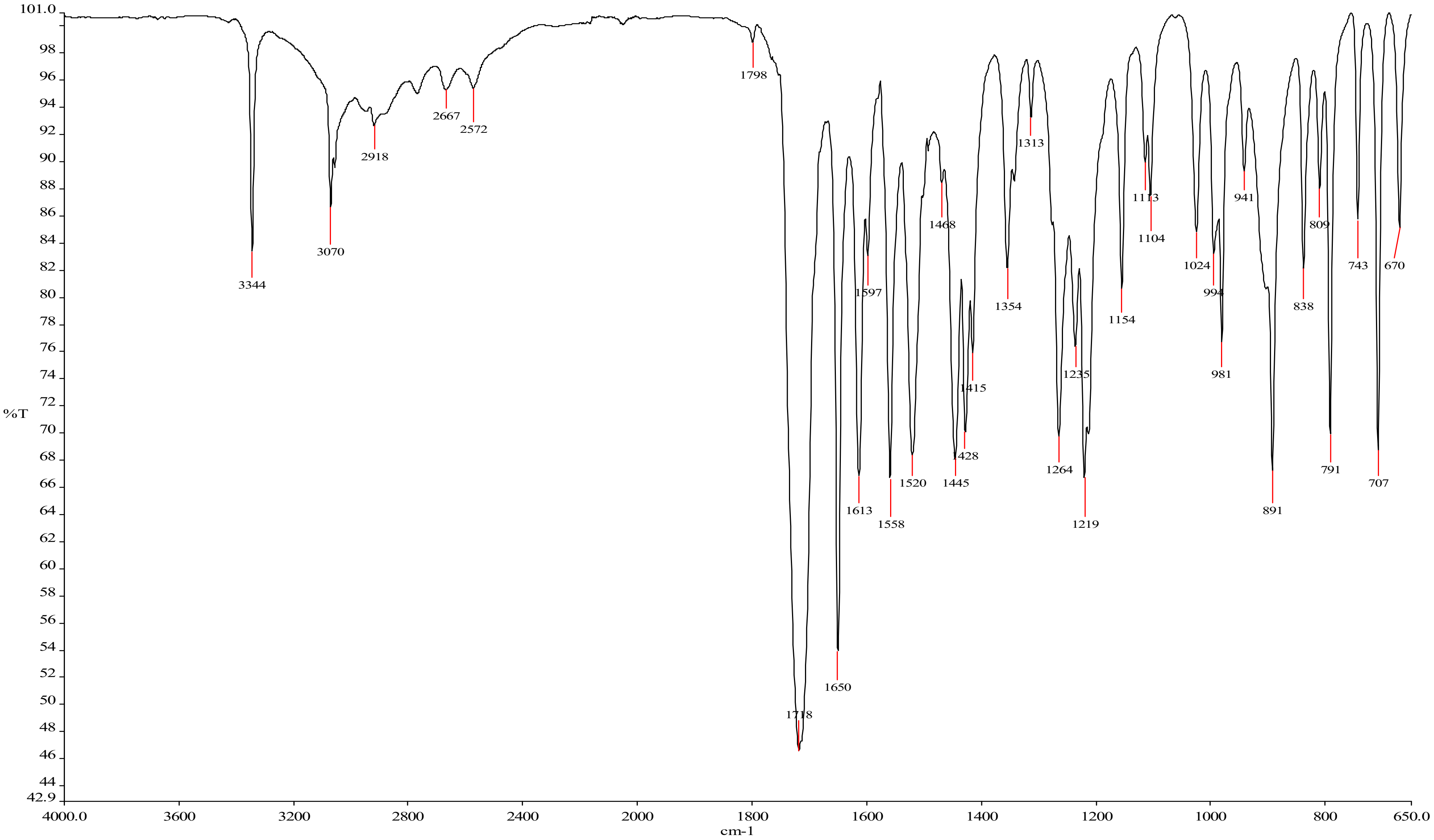

Figure S19: IR spectra of compound **7e**

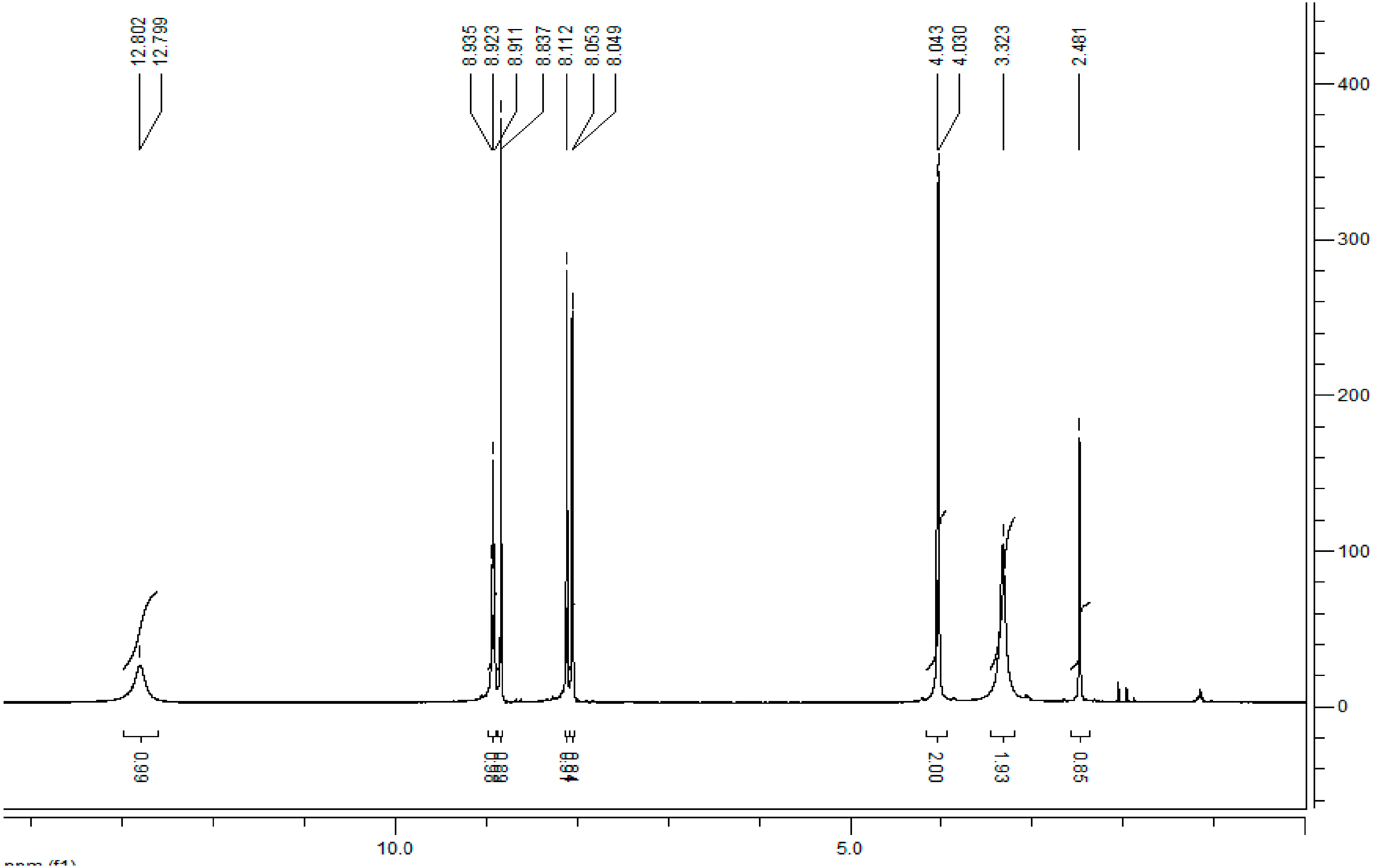

Figure S20: ^{1}H NMR spectra of compound **7e** (DMSO-d_6)

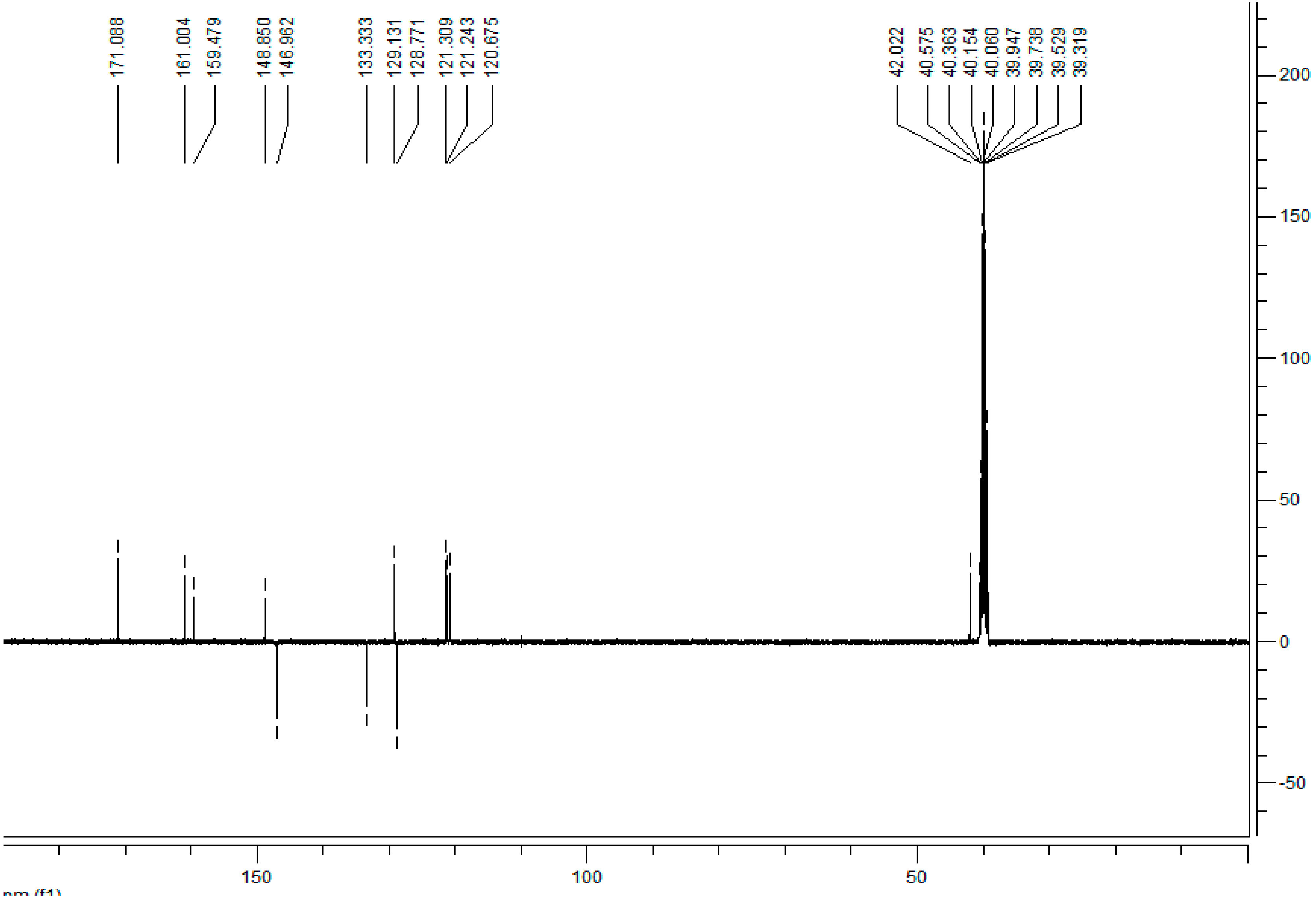

Figure S21: ^{13}C NMR (APT) spectra of compound **7e** (DMSO-d_6)

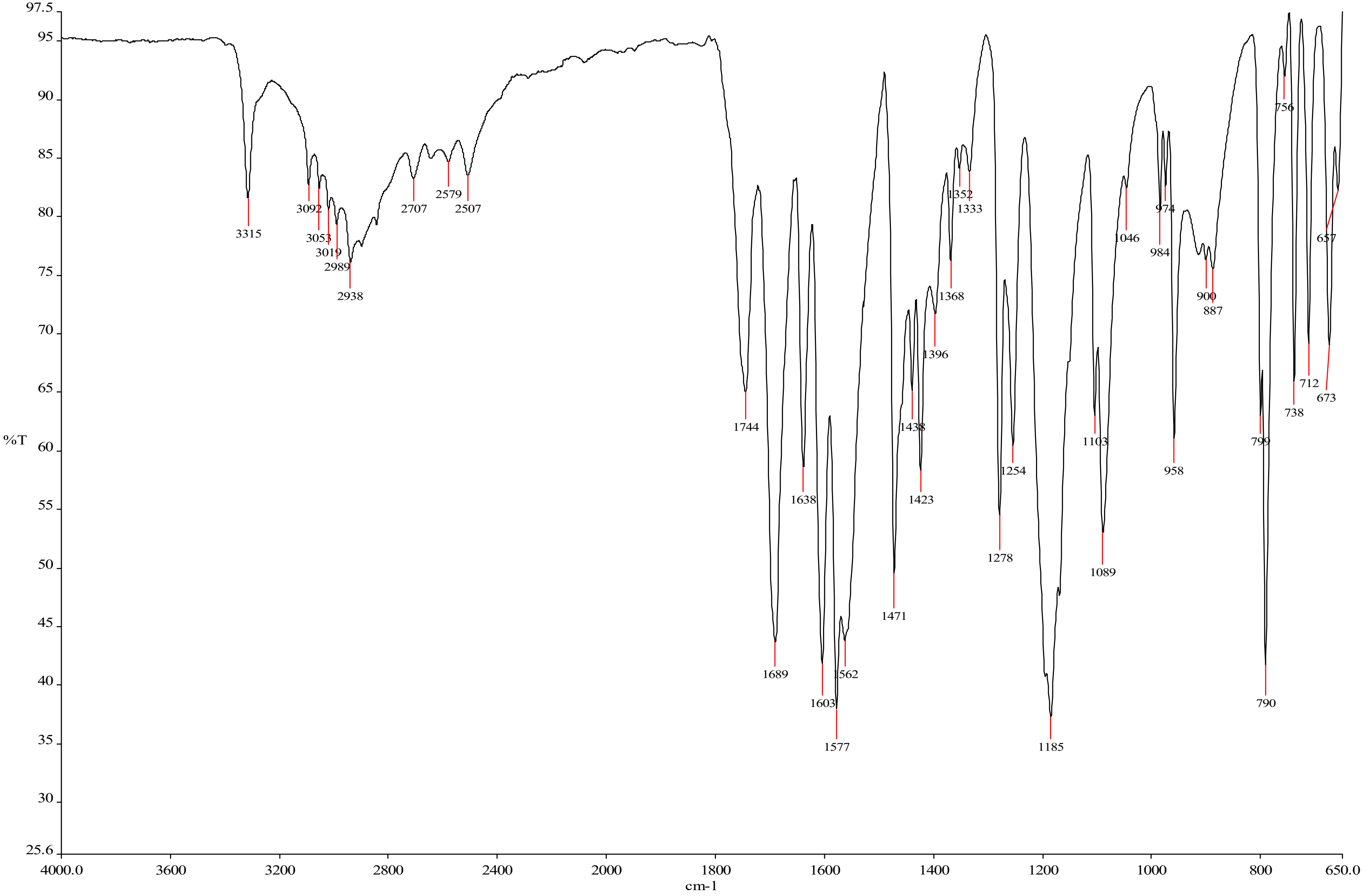

Figure S22: IR spectra of compound **7f**

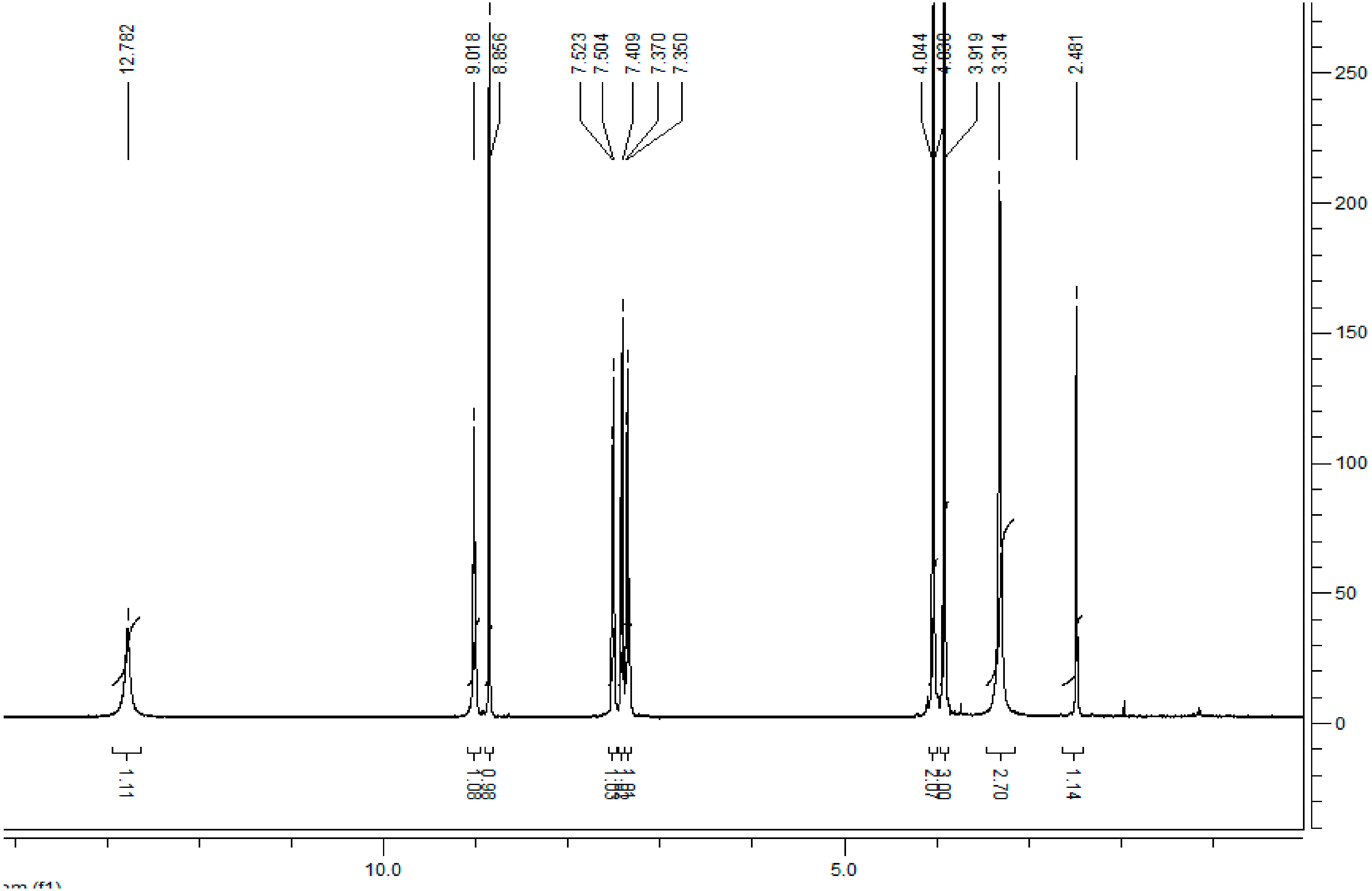

Figure S23: ^{1}H NMR spectra of compound **7f** (DMSO-d_6)

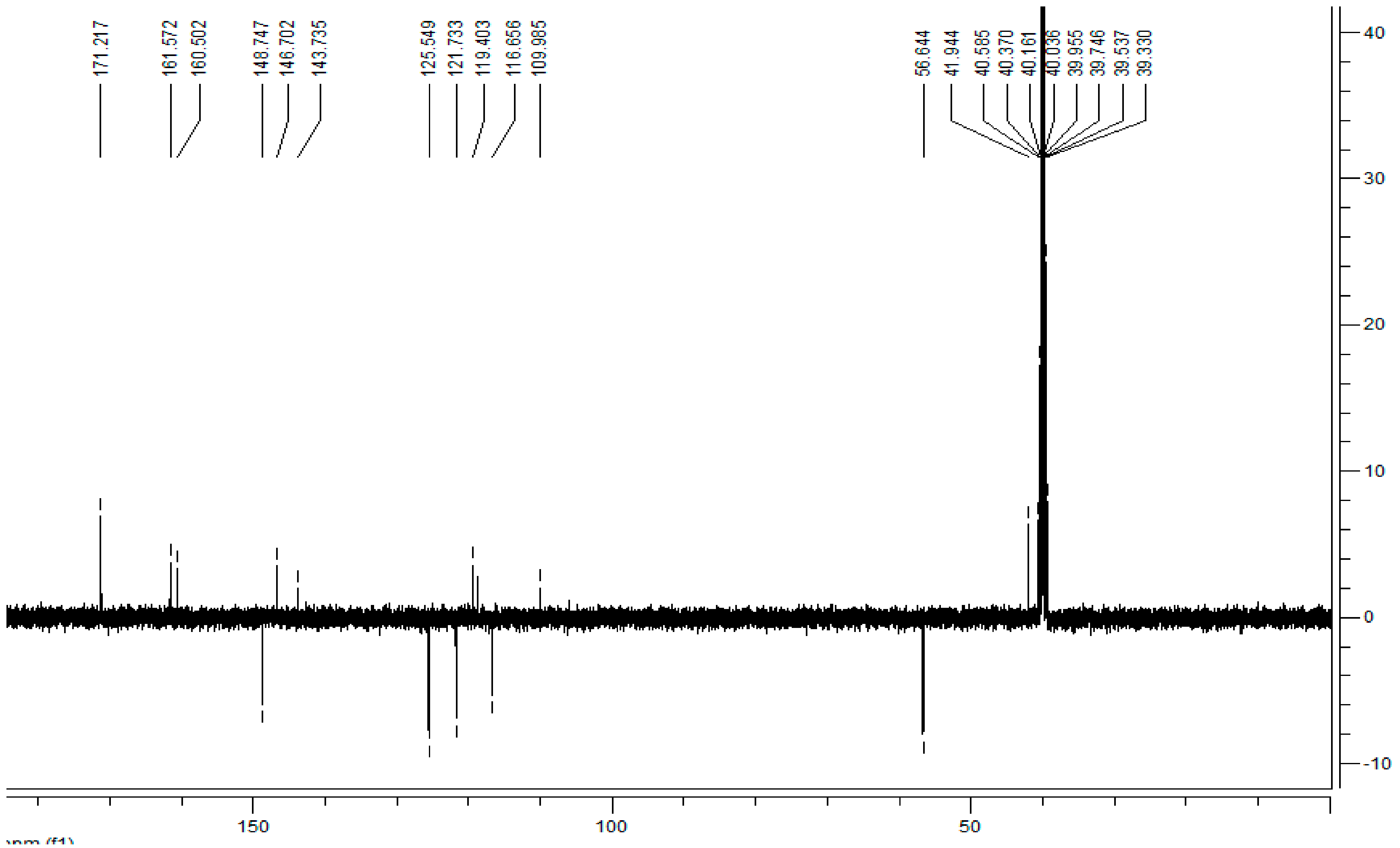

Figure S24: ^{13}C NMR (APT) spectra of compound **7f** (DMSO-d_6)

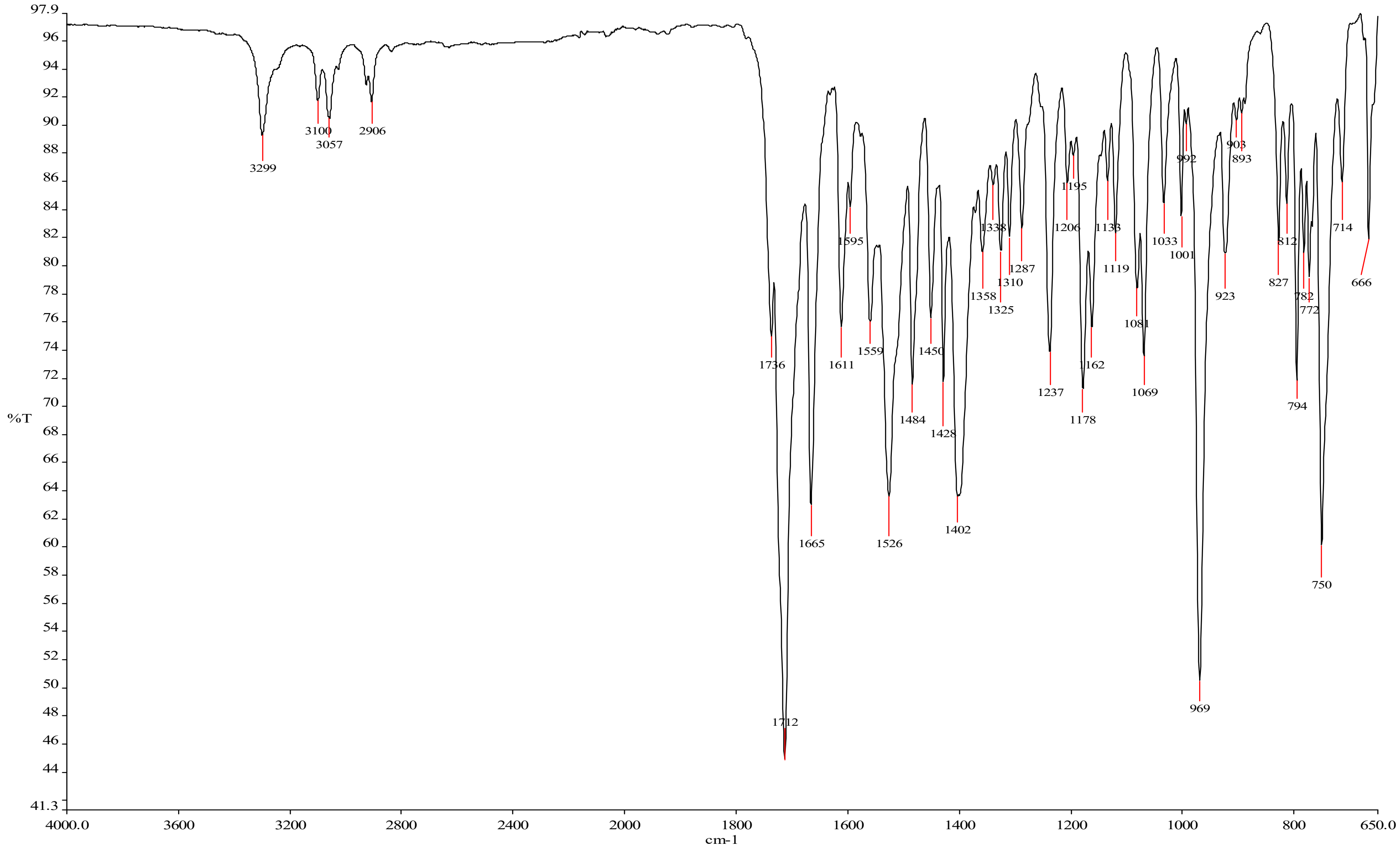

Figure S25: IR spectra of compound **8b**

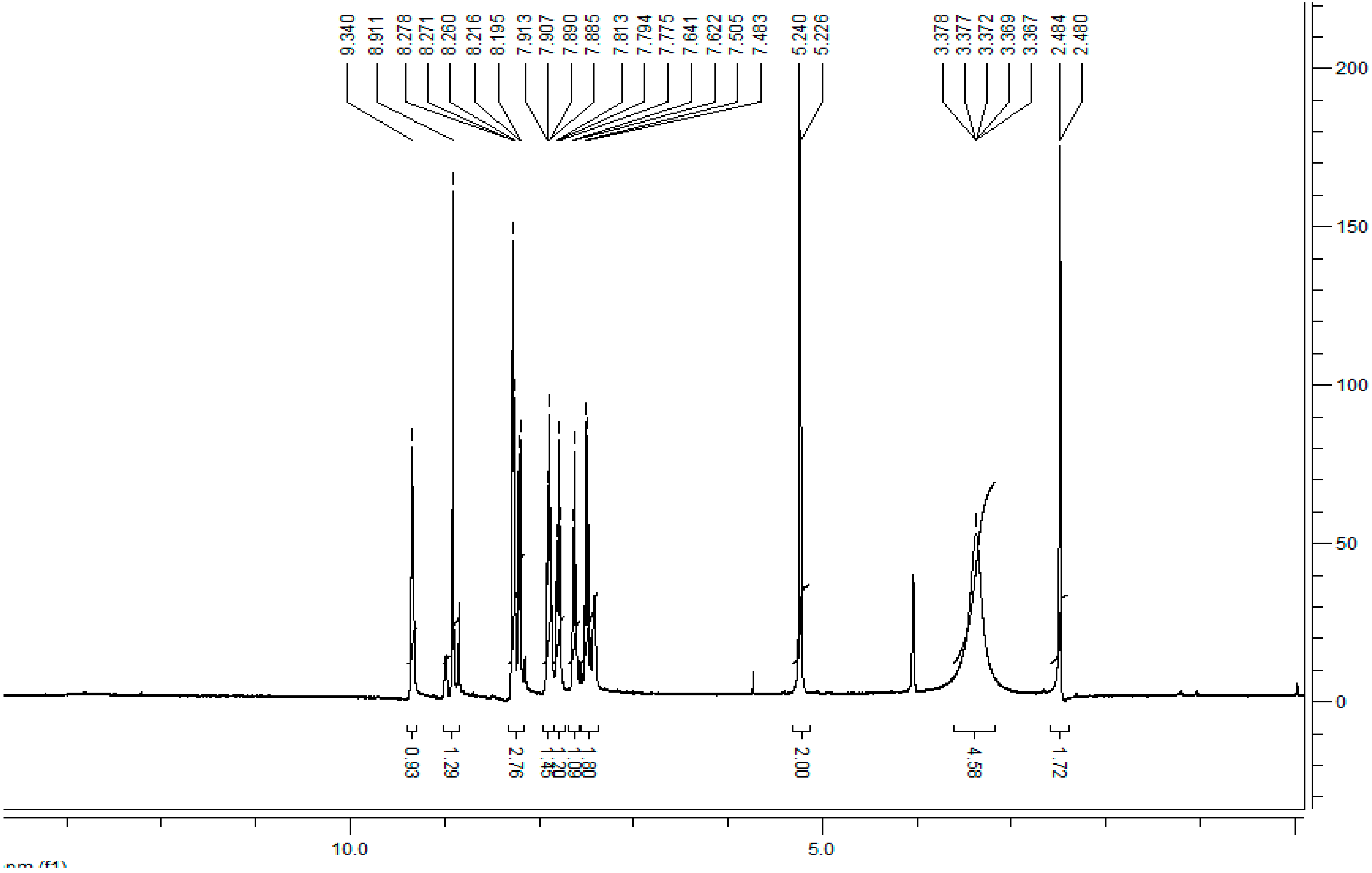

Figure S26: ^{1}H NMR spectra of compound **8b** (DMSO-d_6)

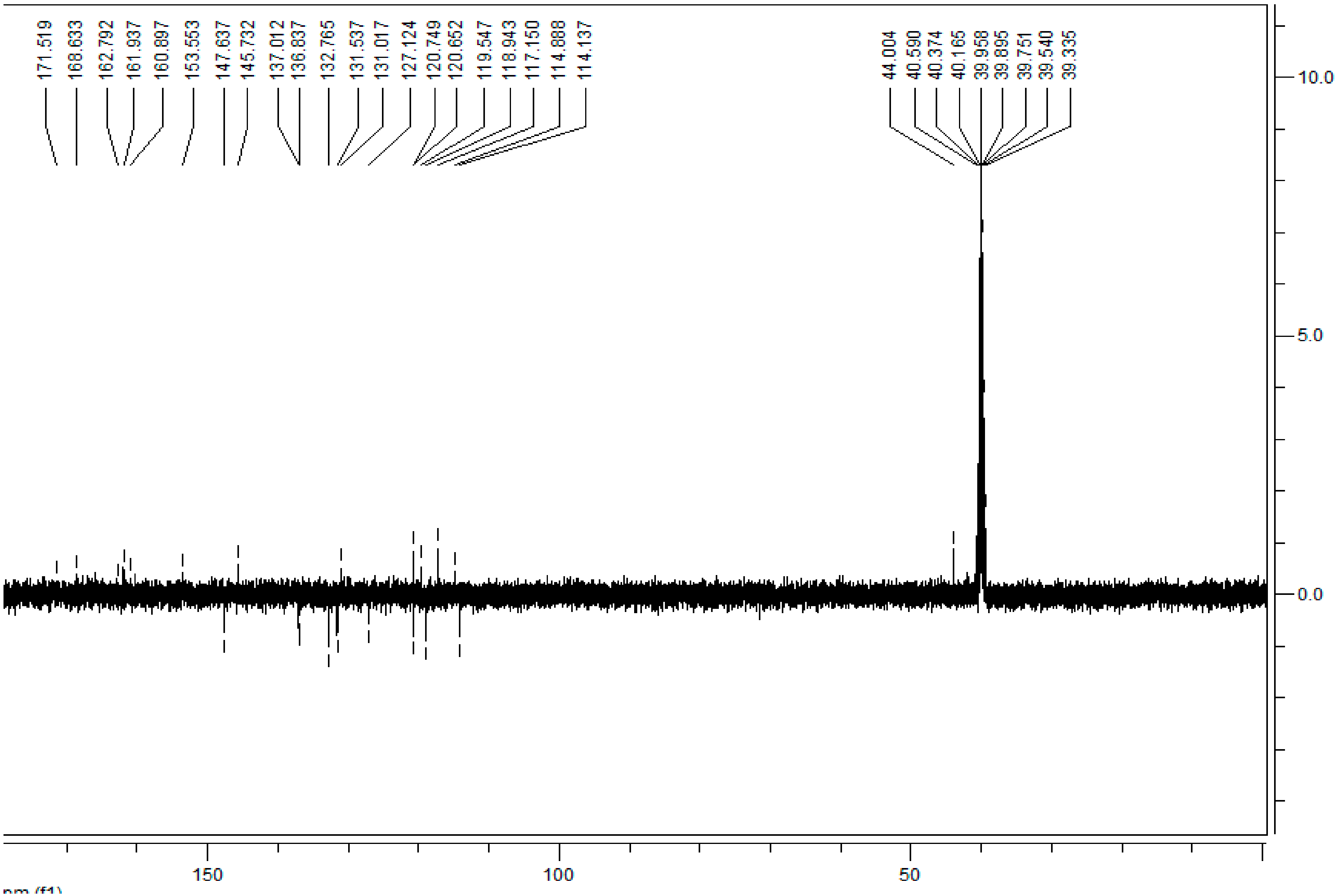

Figure S27: ^{13}C NMR (APT) spectra of compound **8b** (DMSO-d_6)

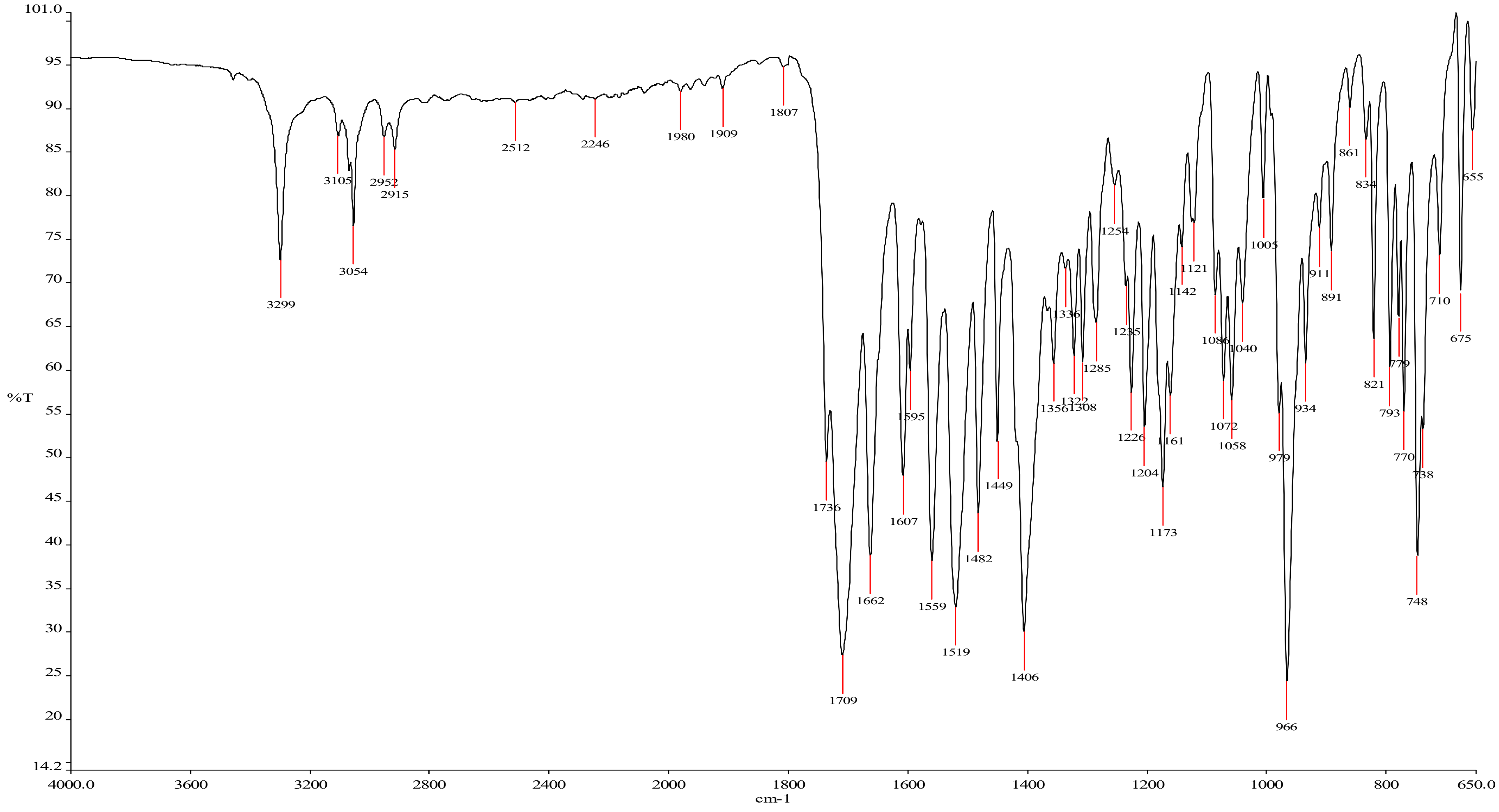

Figure S28: IR spectra of compound **8c**

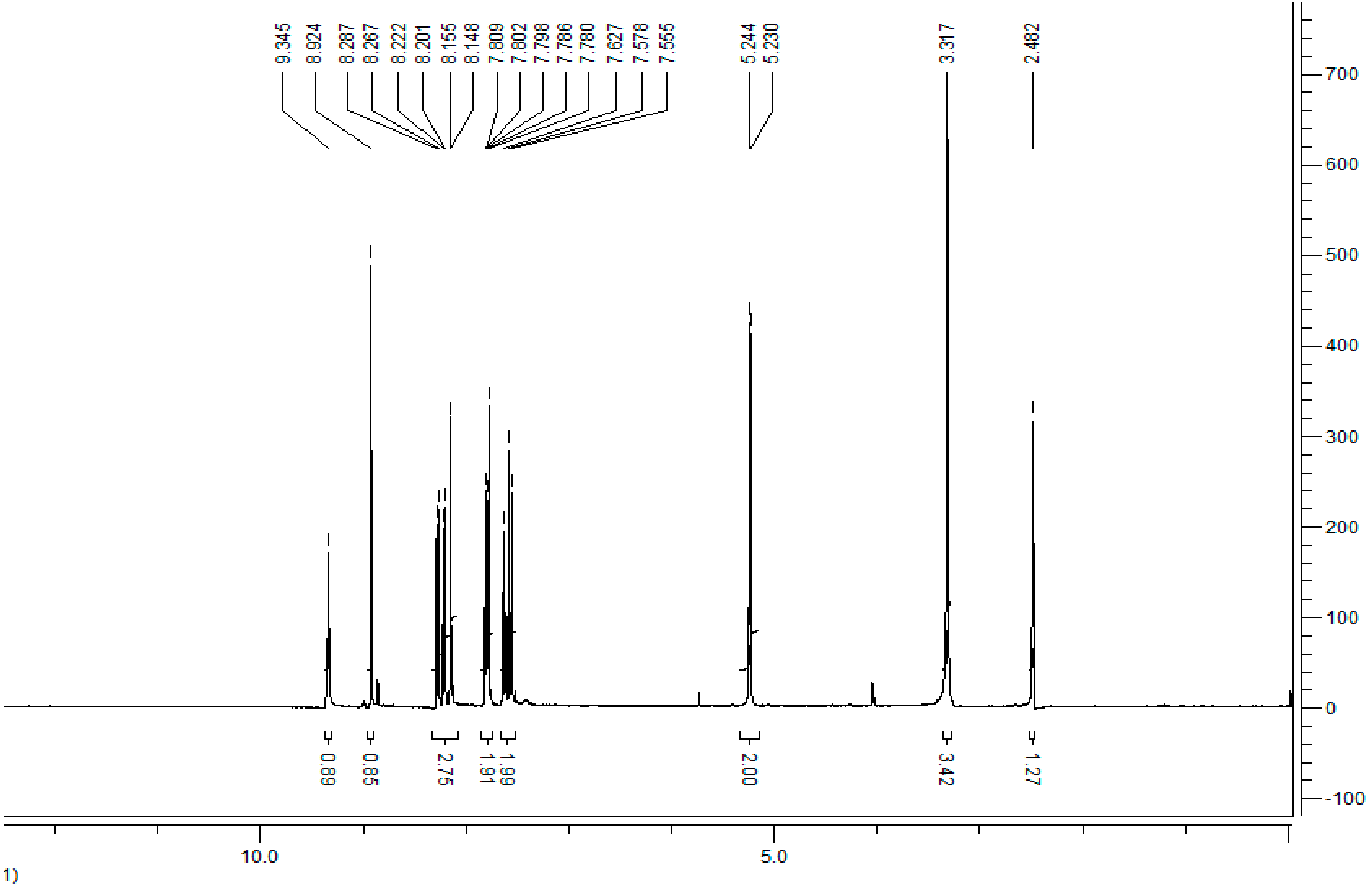

Figure S29: ^{1}H NMR spectra of compound **8c** (DMSO-d_6)

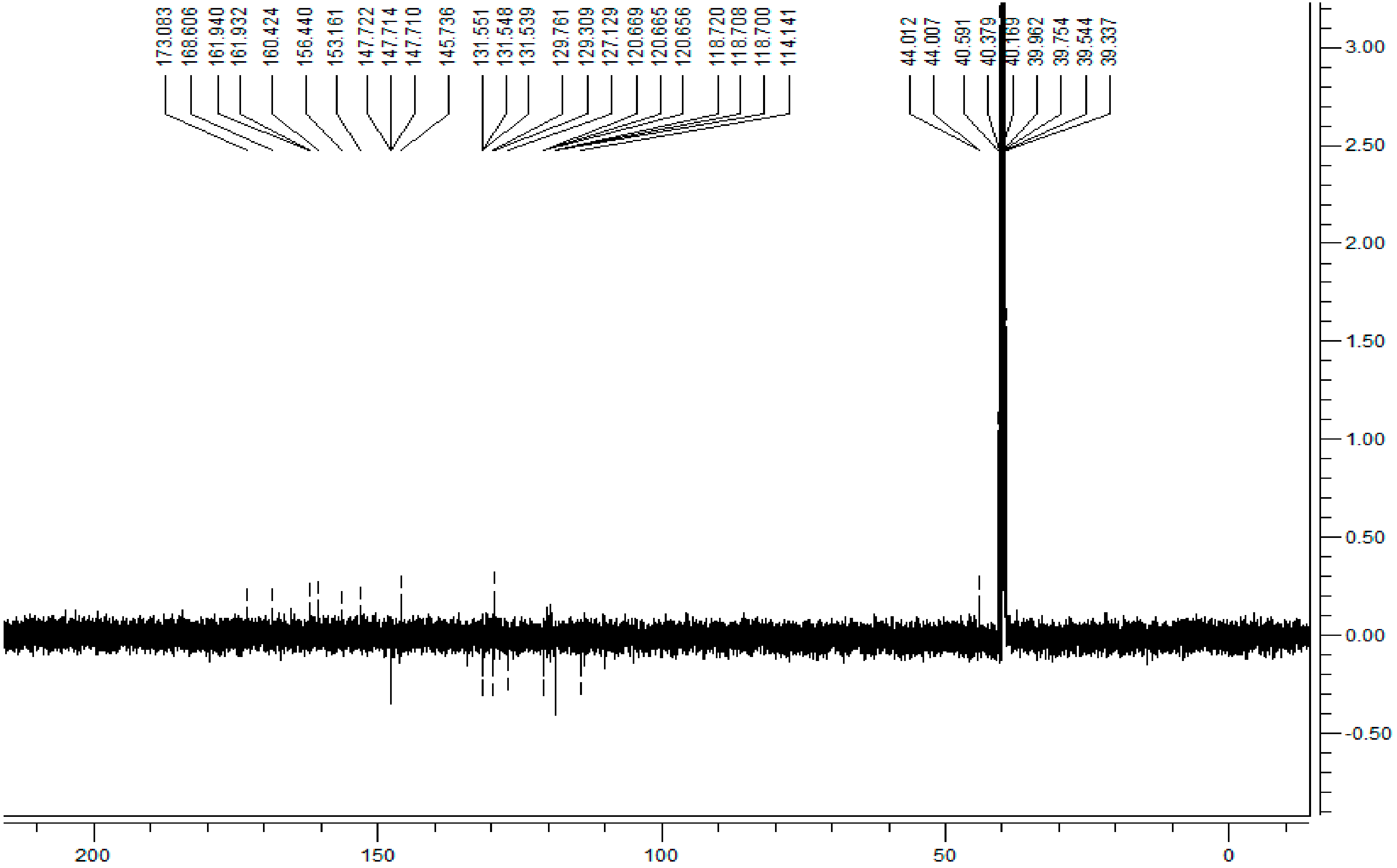

Figure S30: ^{13}C NMR (APT) spectra of compound **8c** (DMSO-d_6)

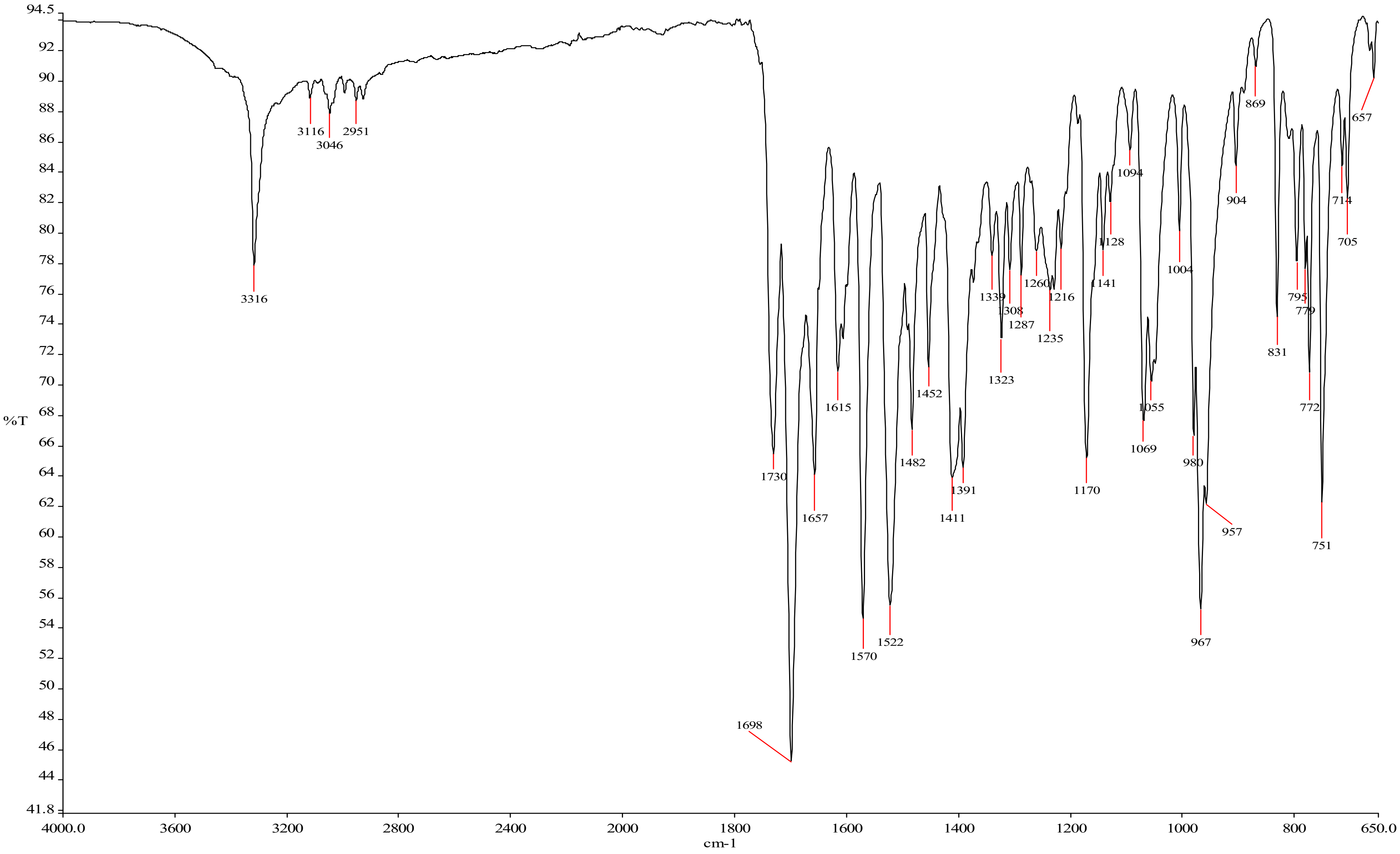

Figure S31: IR spectra of compound **8d**

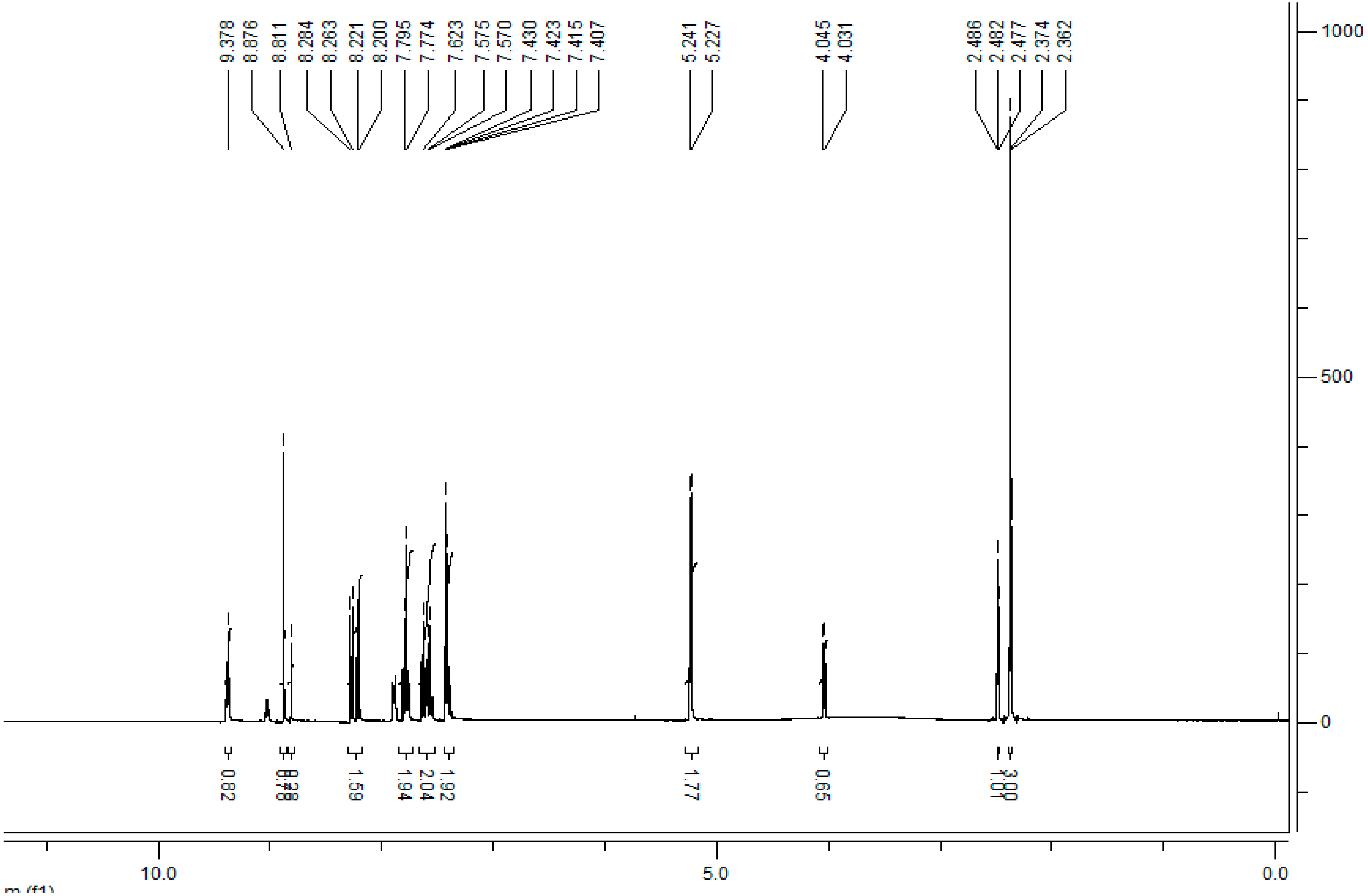

Figure S32: ^{1}H NMR spectra of compound **8d** (DMSO-d$_6$)

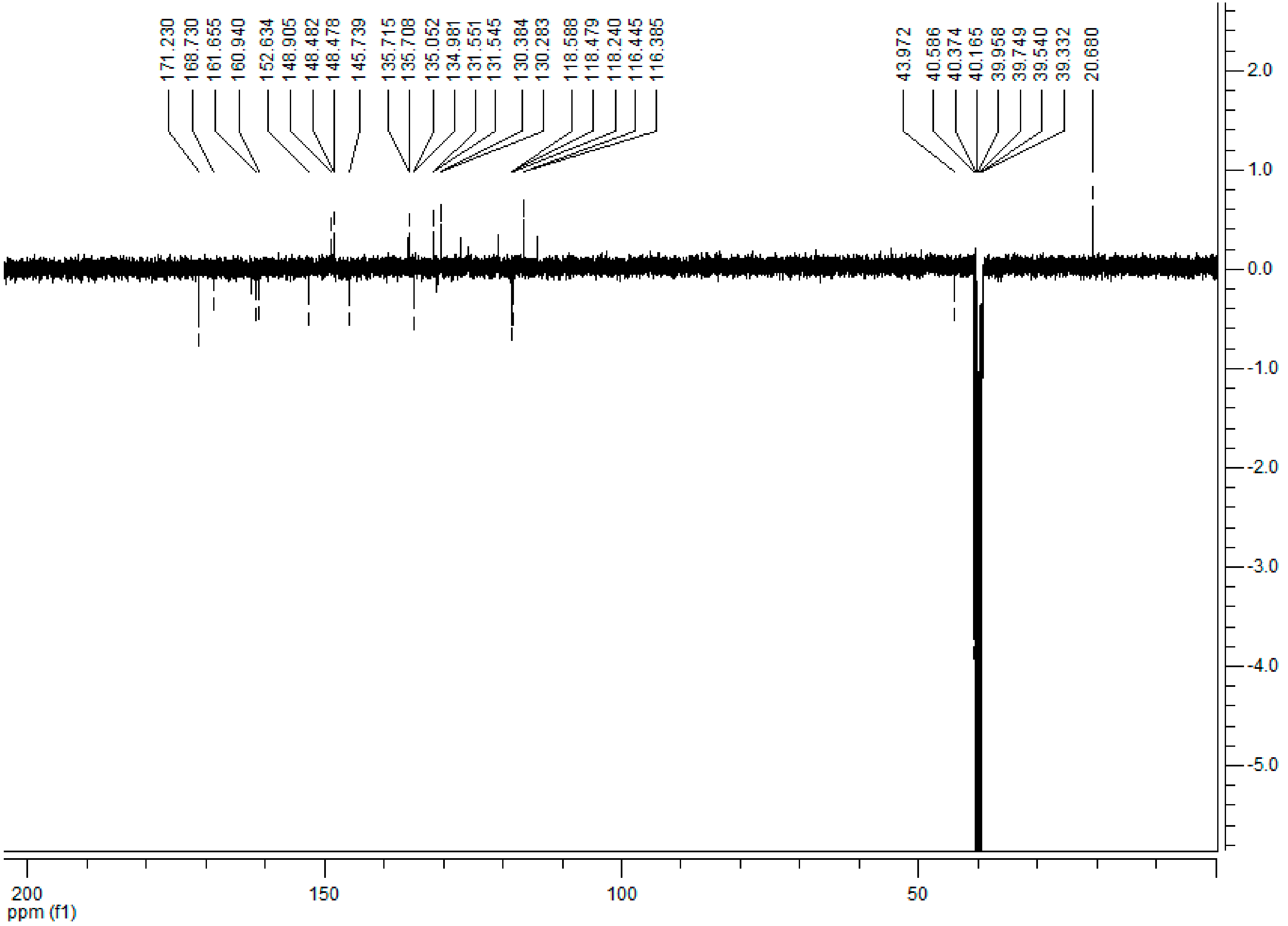

Figure S33: ^{13}C NMR spectra of compound **8d** (DMSO-d_6)

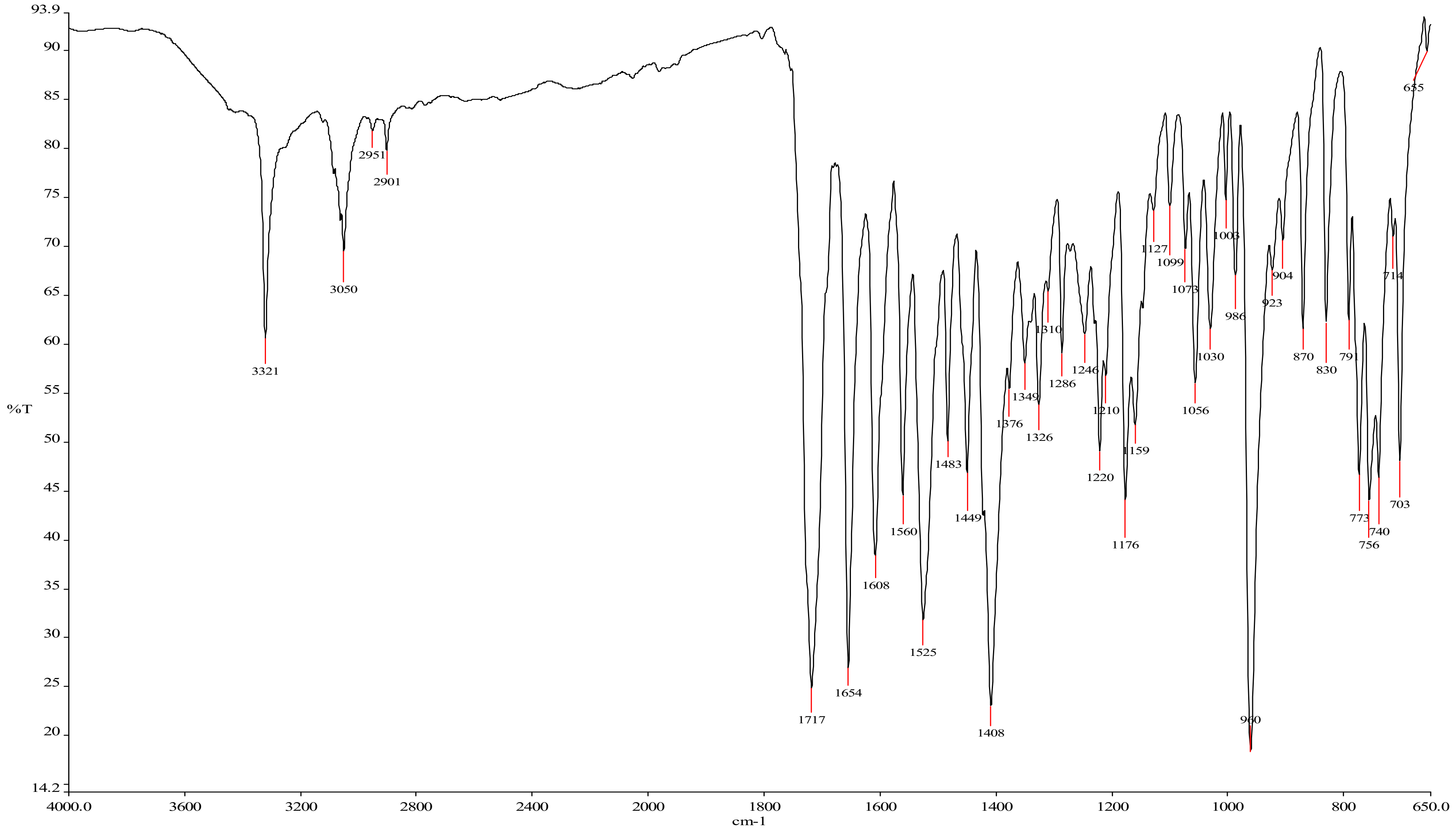

Figure S34: IR spectra of compound **8e**

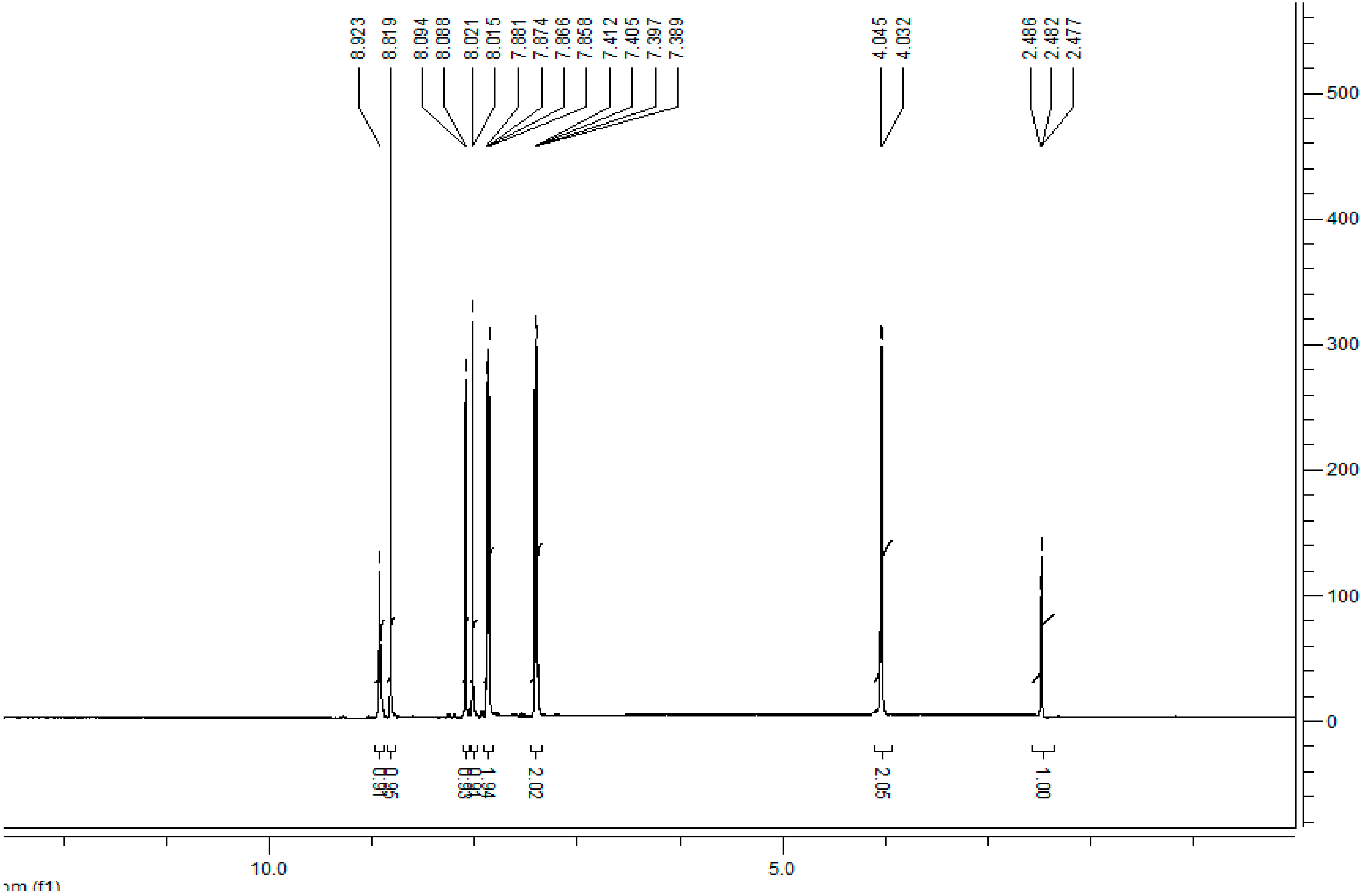

Figure S35: ^{1}H NMR spectra of compound **8e** (DMSO-d_6)

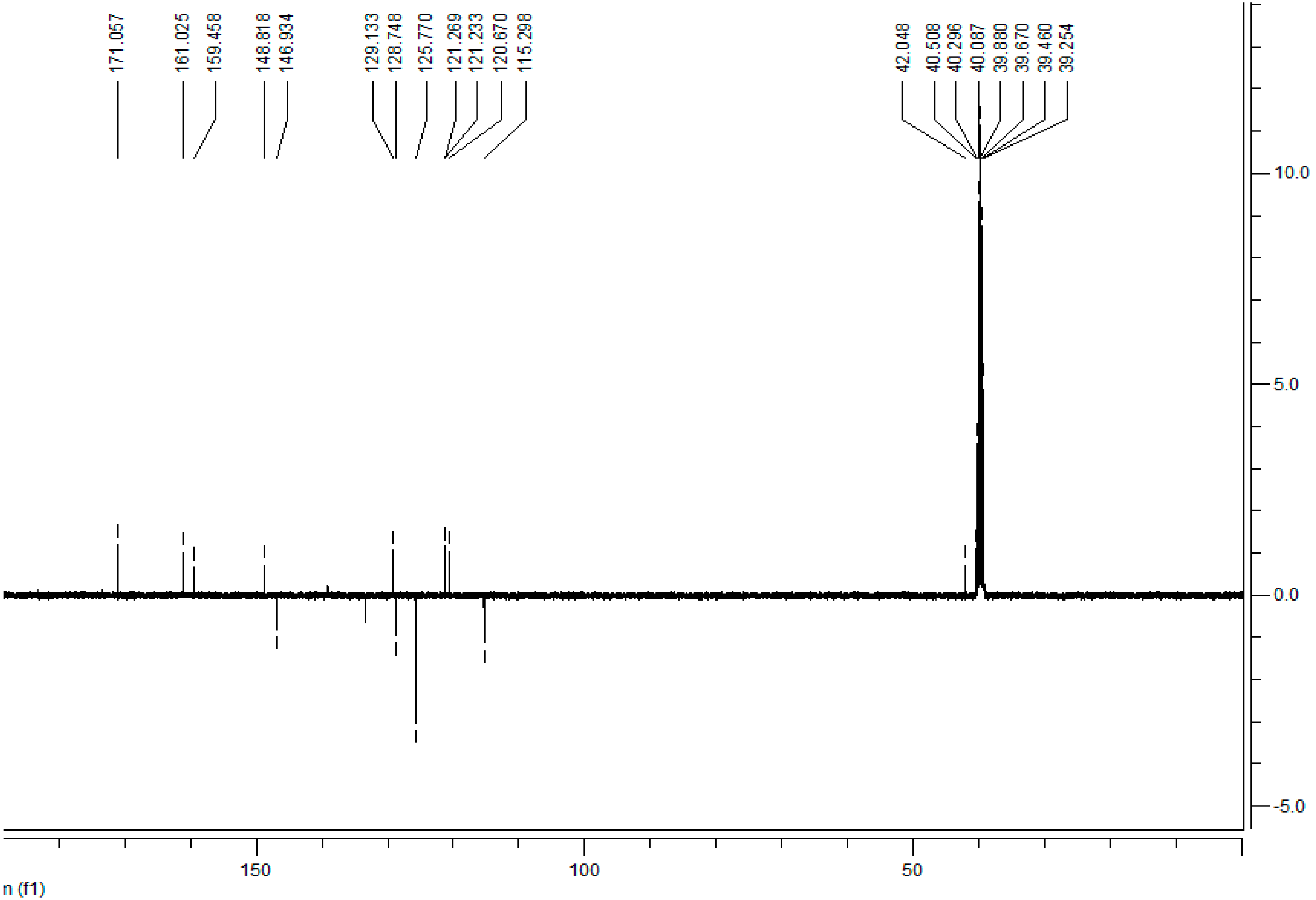

Figure S36: ^{13}C NMR (APT) spectra of compound **8e** (DMSO-d_6)

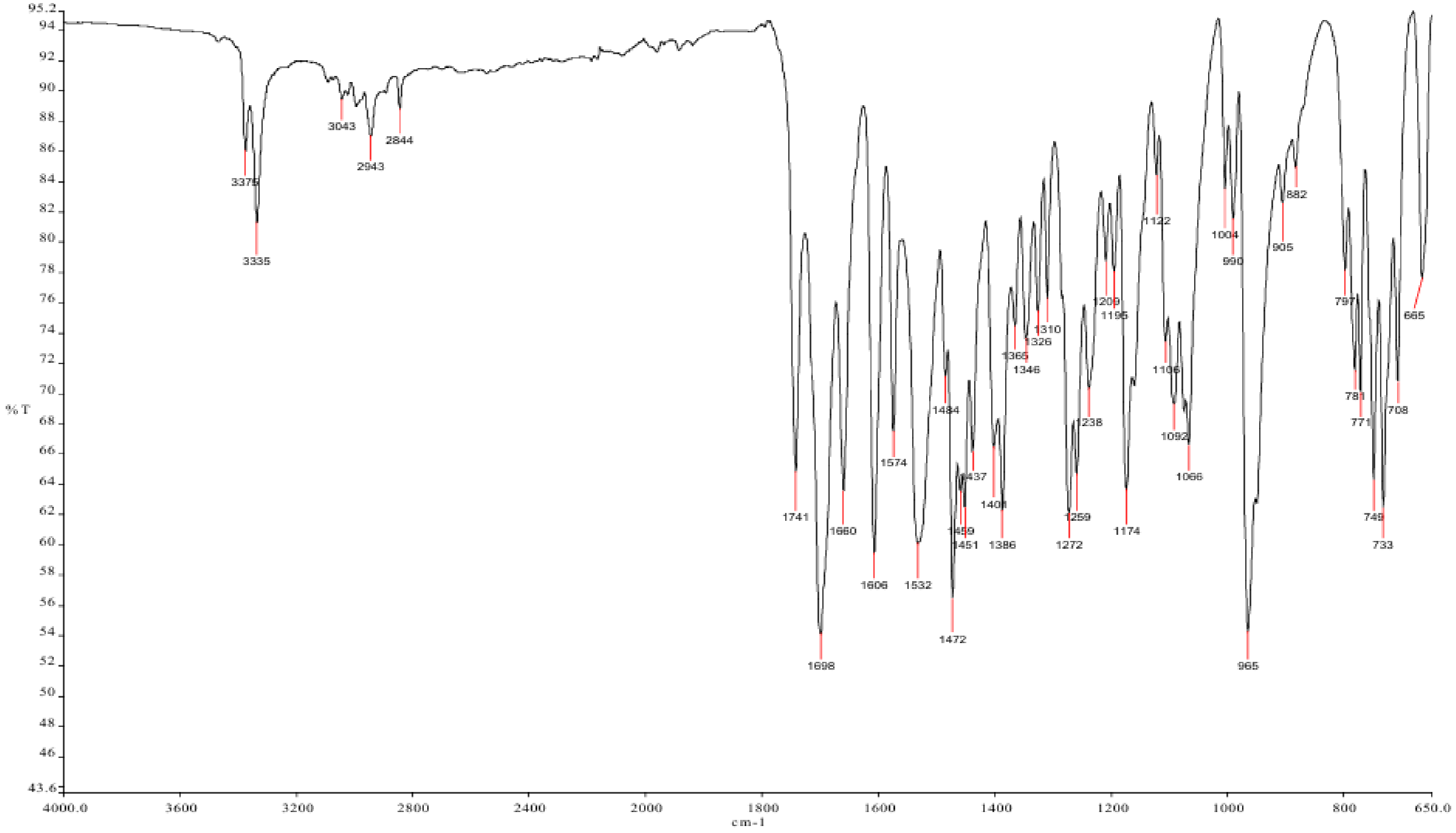

Figure S37: IR spectra of compound **8f**

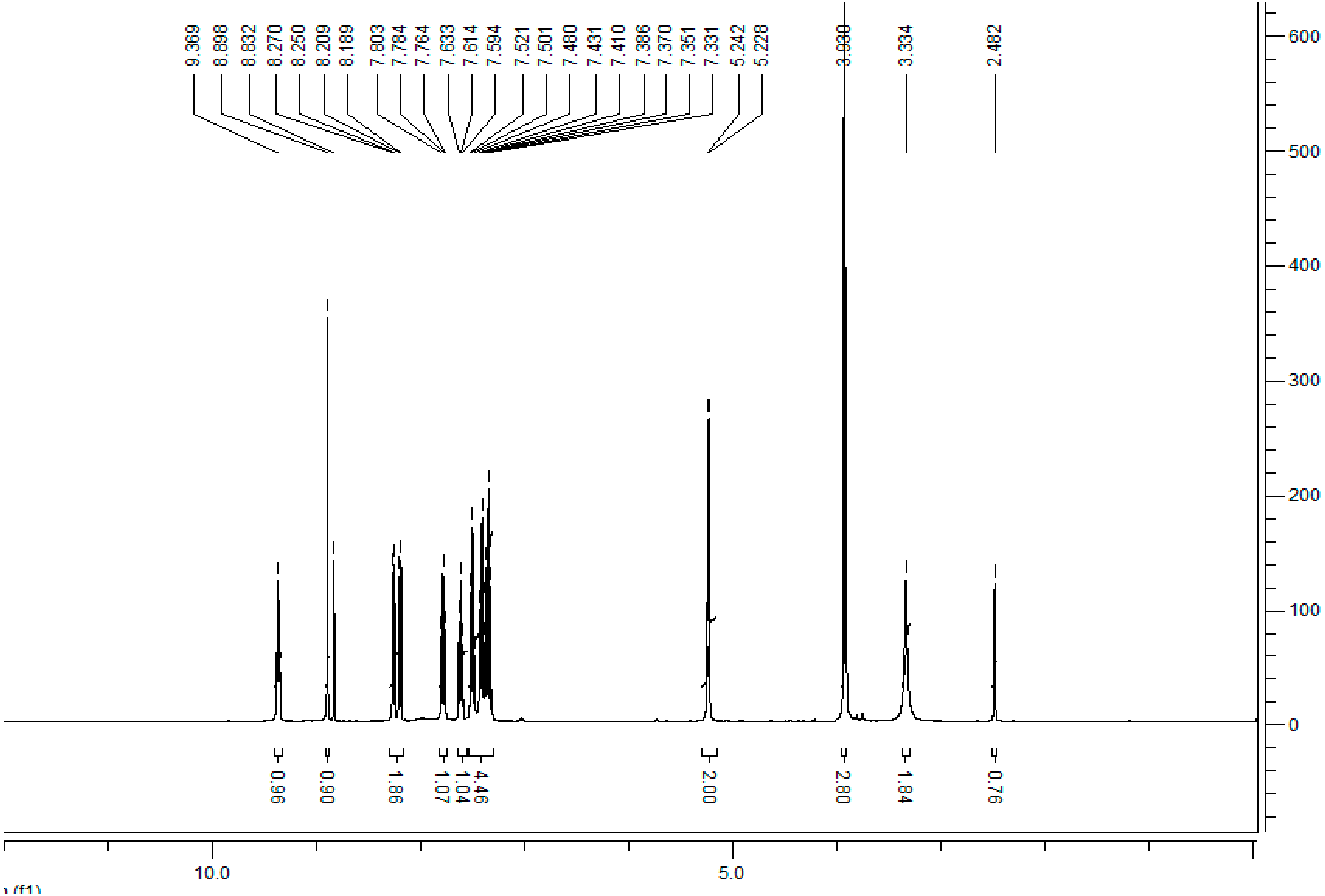

Figure S38: ^{1}H NMR spectra of compound **8f** (DMSO-d_6)

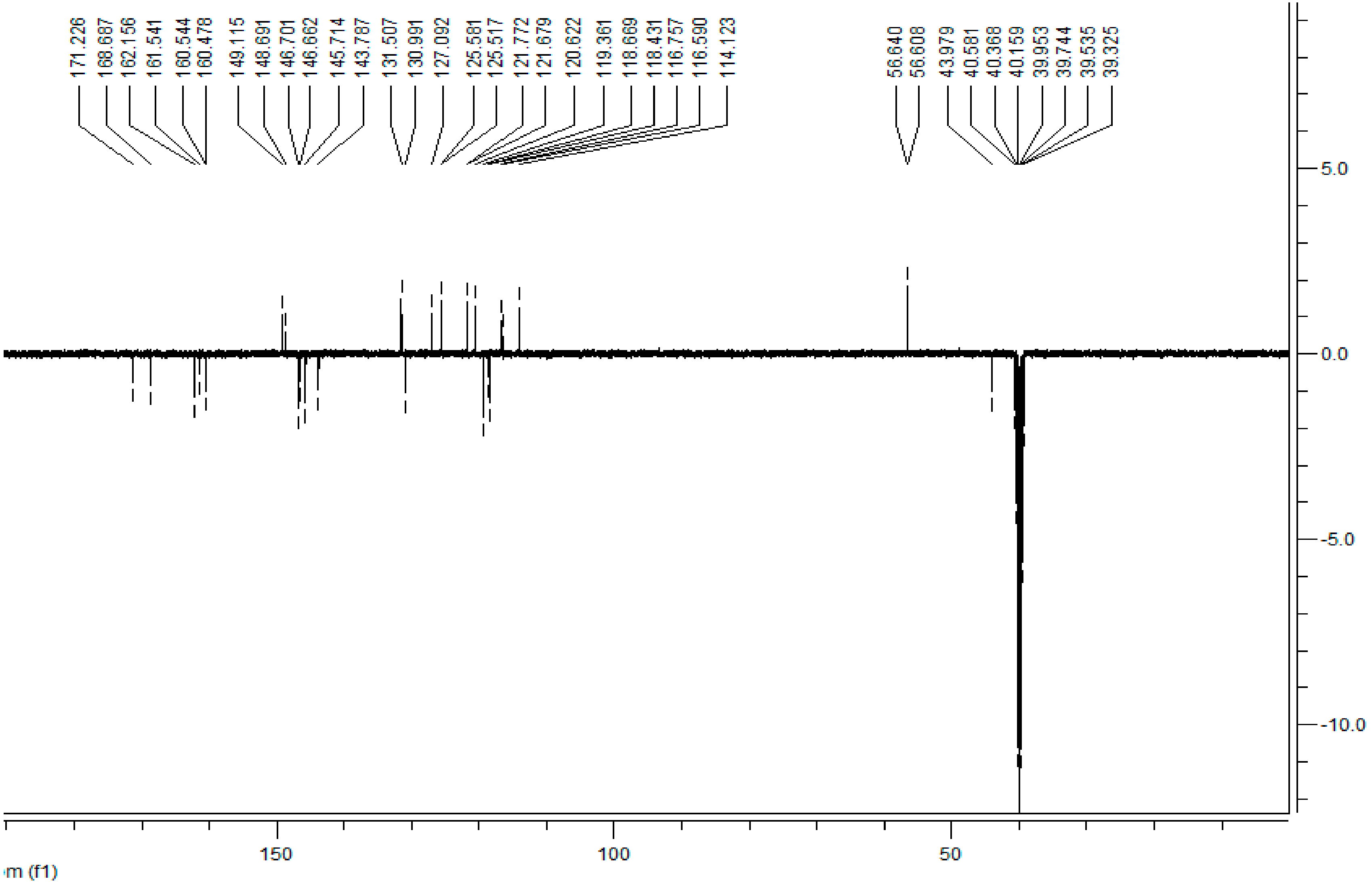

Figure S39: ^{13}C NMR (APT) spectra of compound **8f** (DMSO-d_6)

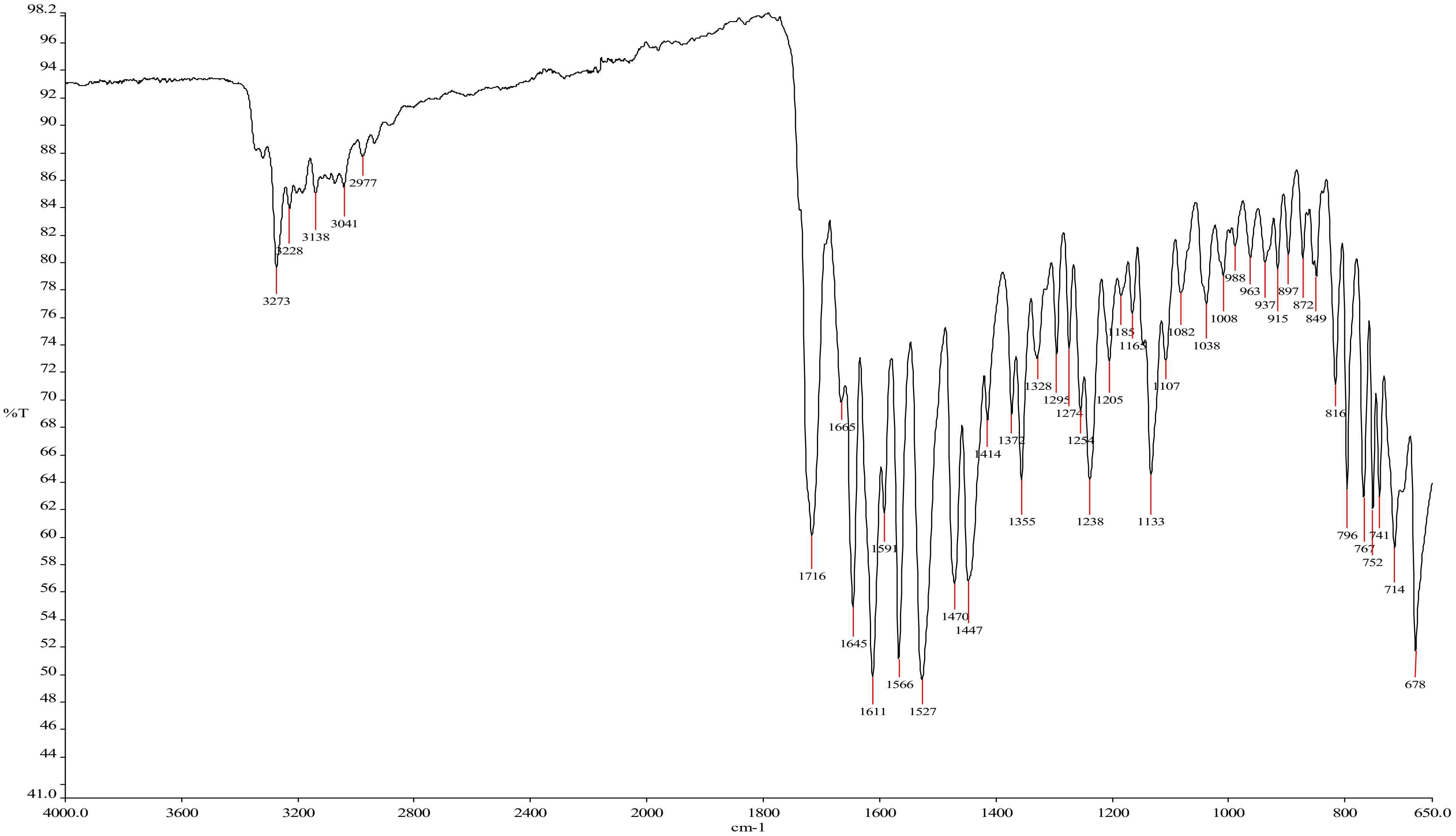

Figure S40: IR spectra of compound **9a**

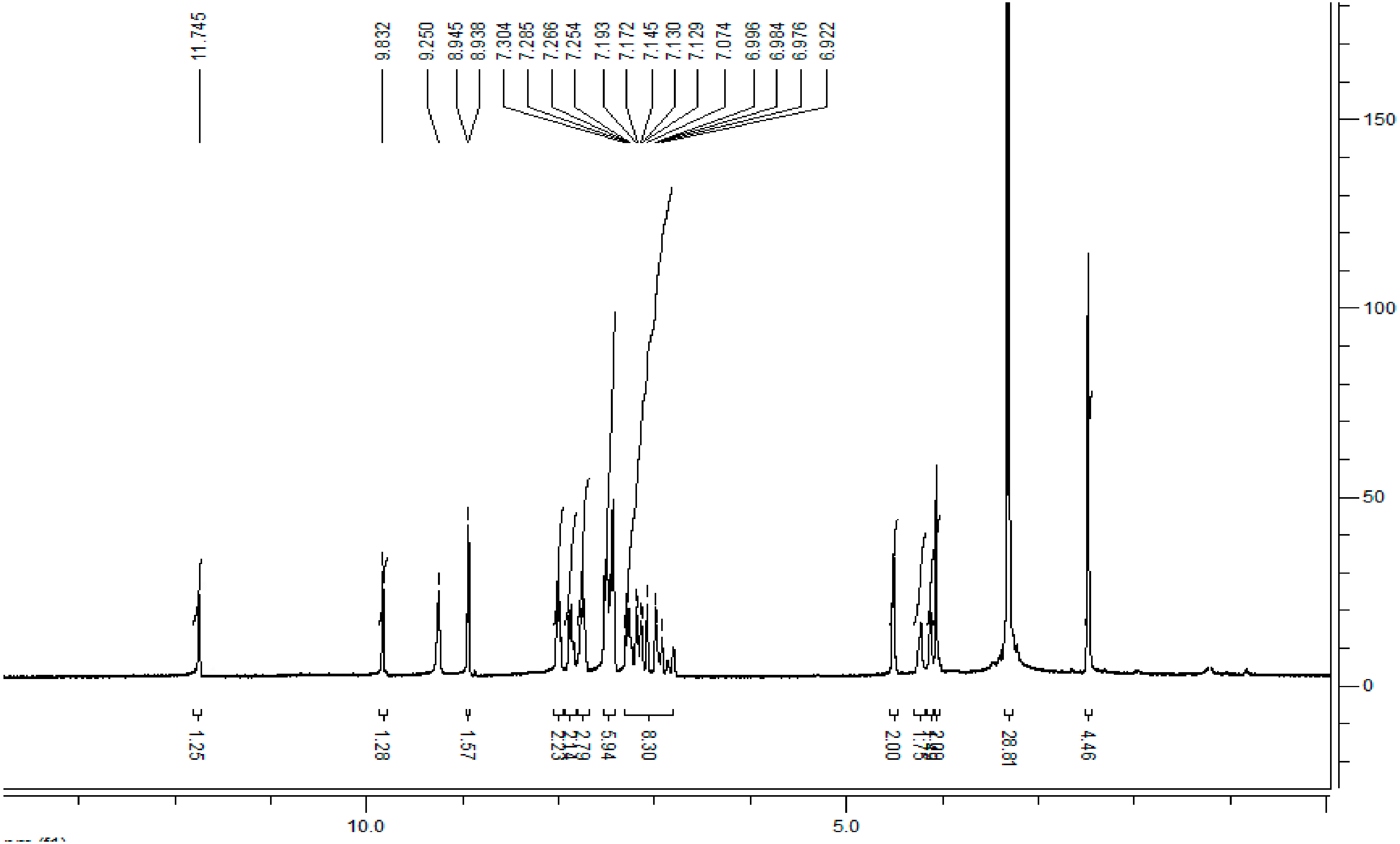

Figure S41: ^{1}H NMR spectra of compound **9a** (DMSO-d_6)

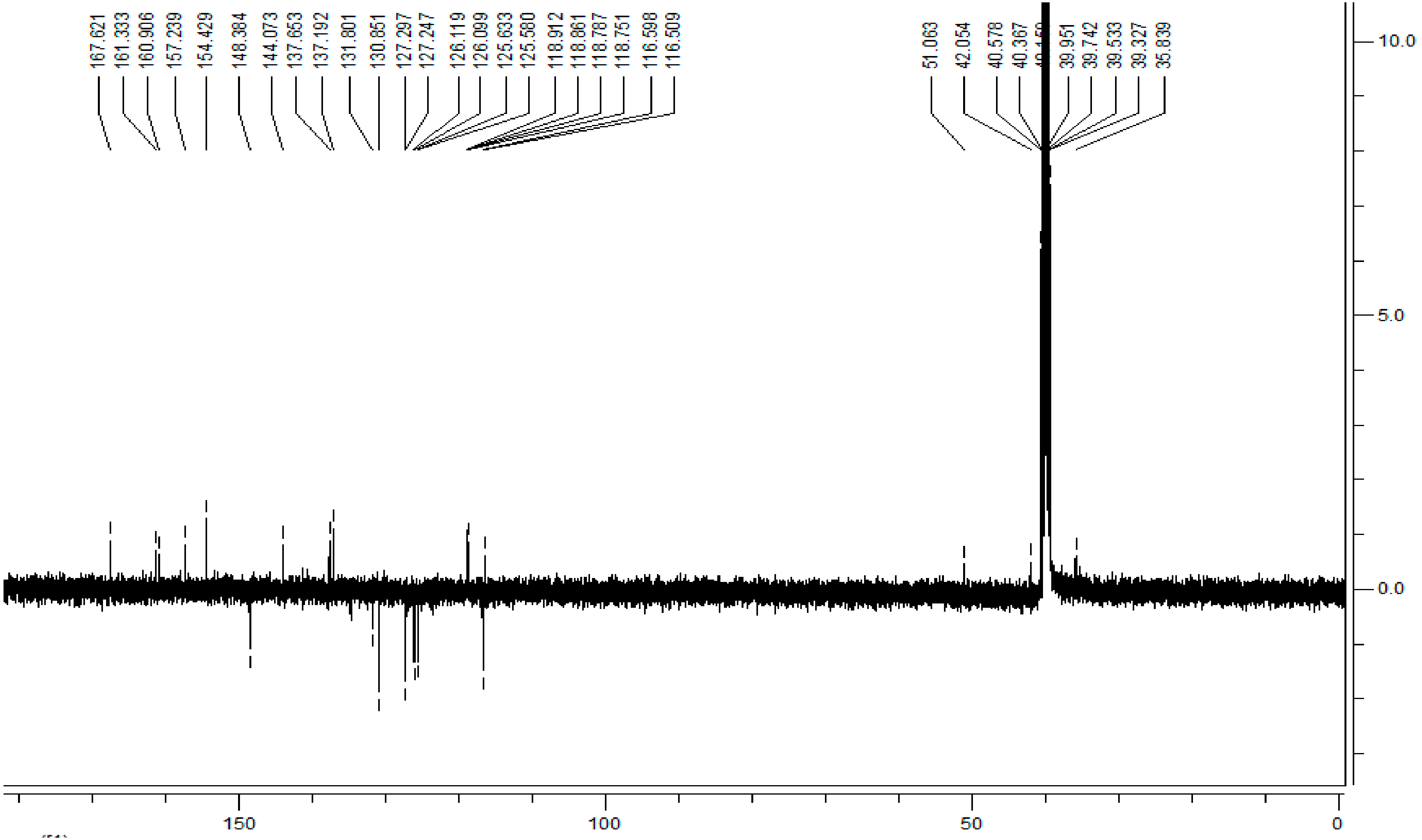

Figure S42: ^{13}C NMR (APT) spectra of compound **9a** (DMSO-d_6)

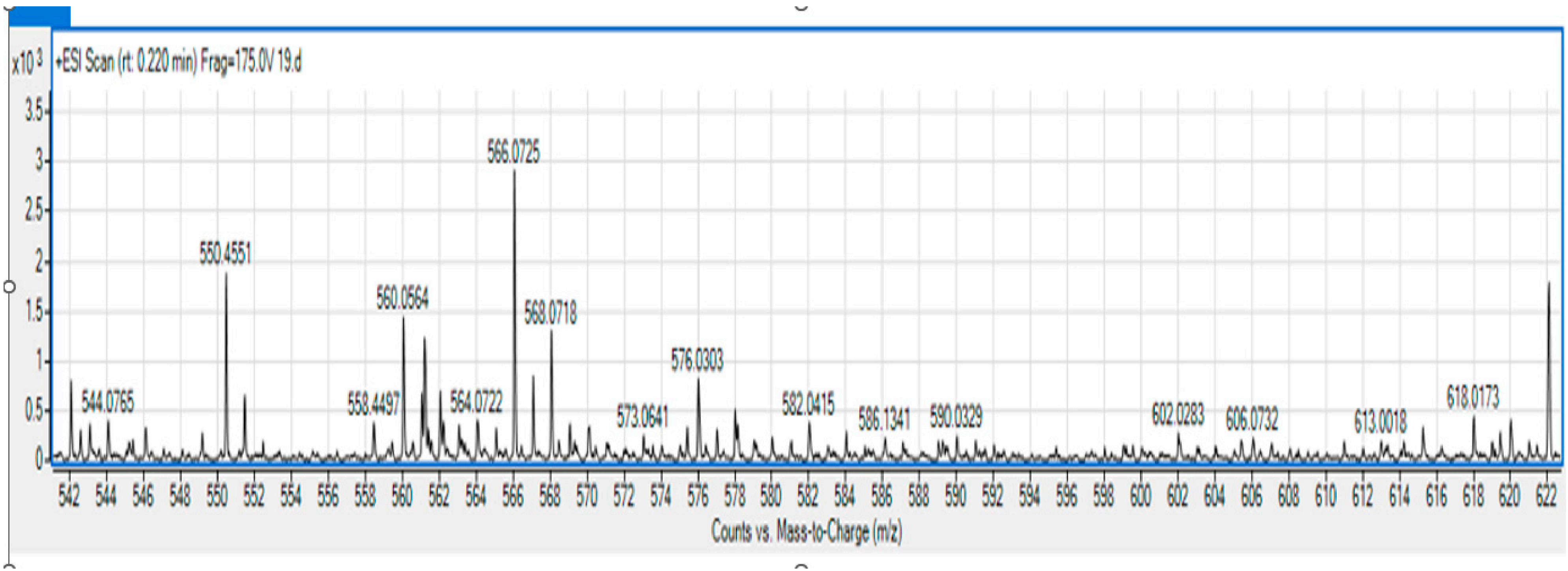

Figure S43: LC MS spectra of compound **9a**

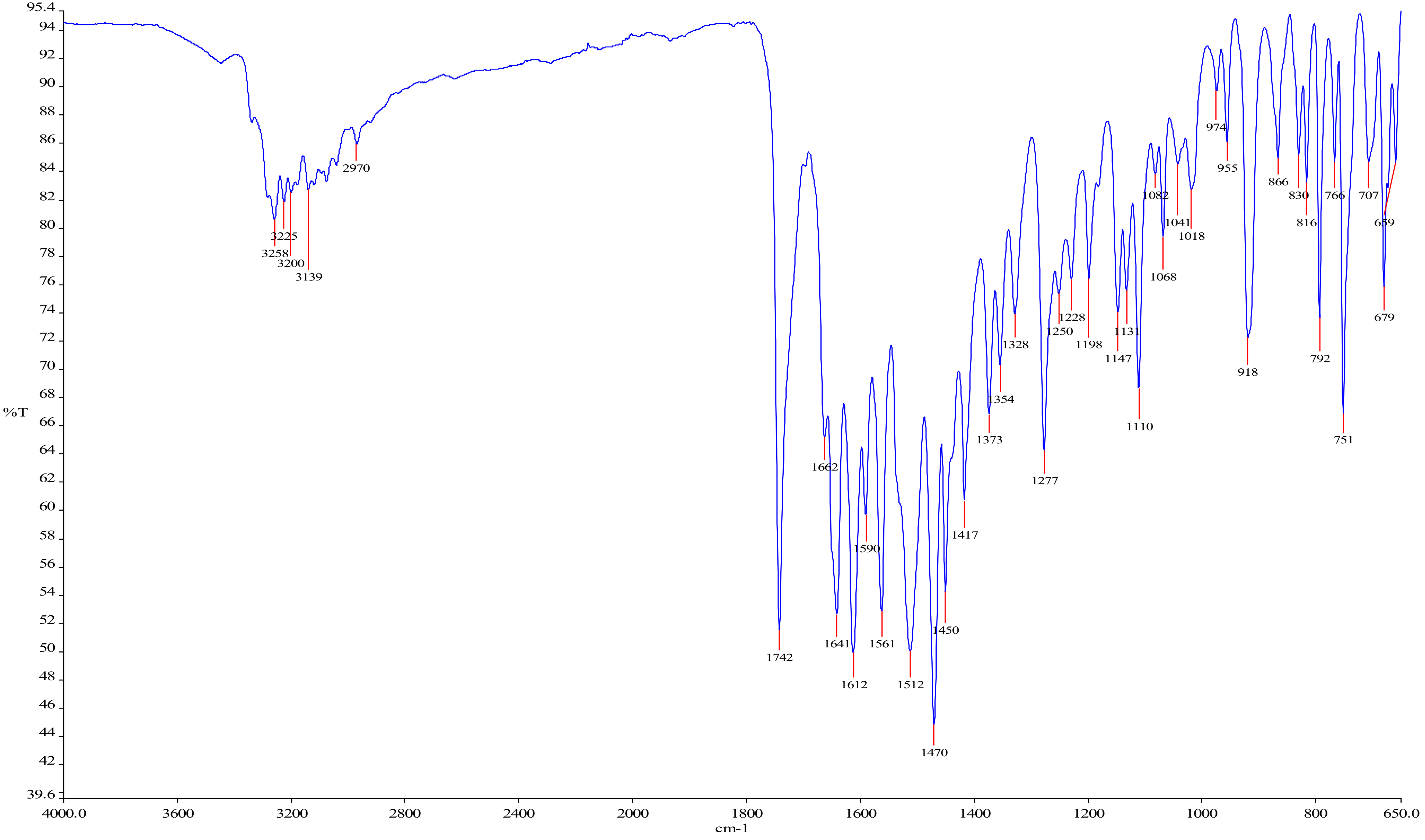

Figure S44: IR spectra of compound **9b**

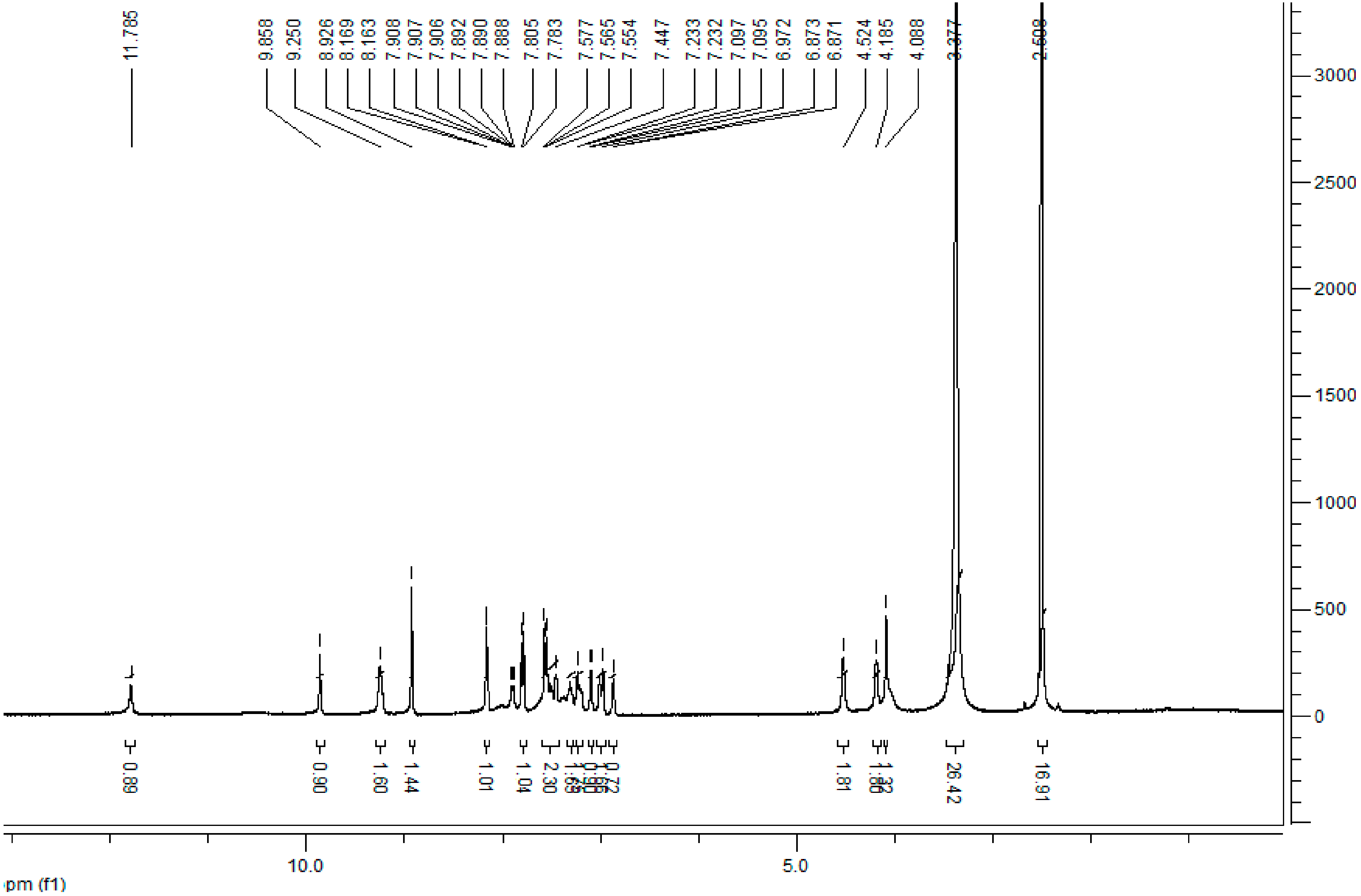

Figure S45: ^{1}H NMR spectra of compound **9b** (DMSO-d_6)

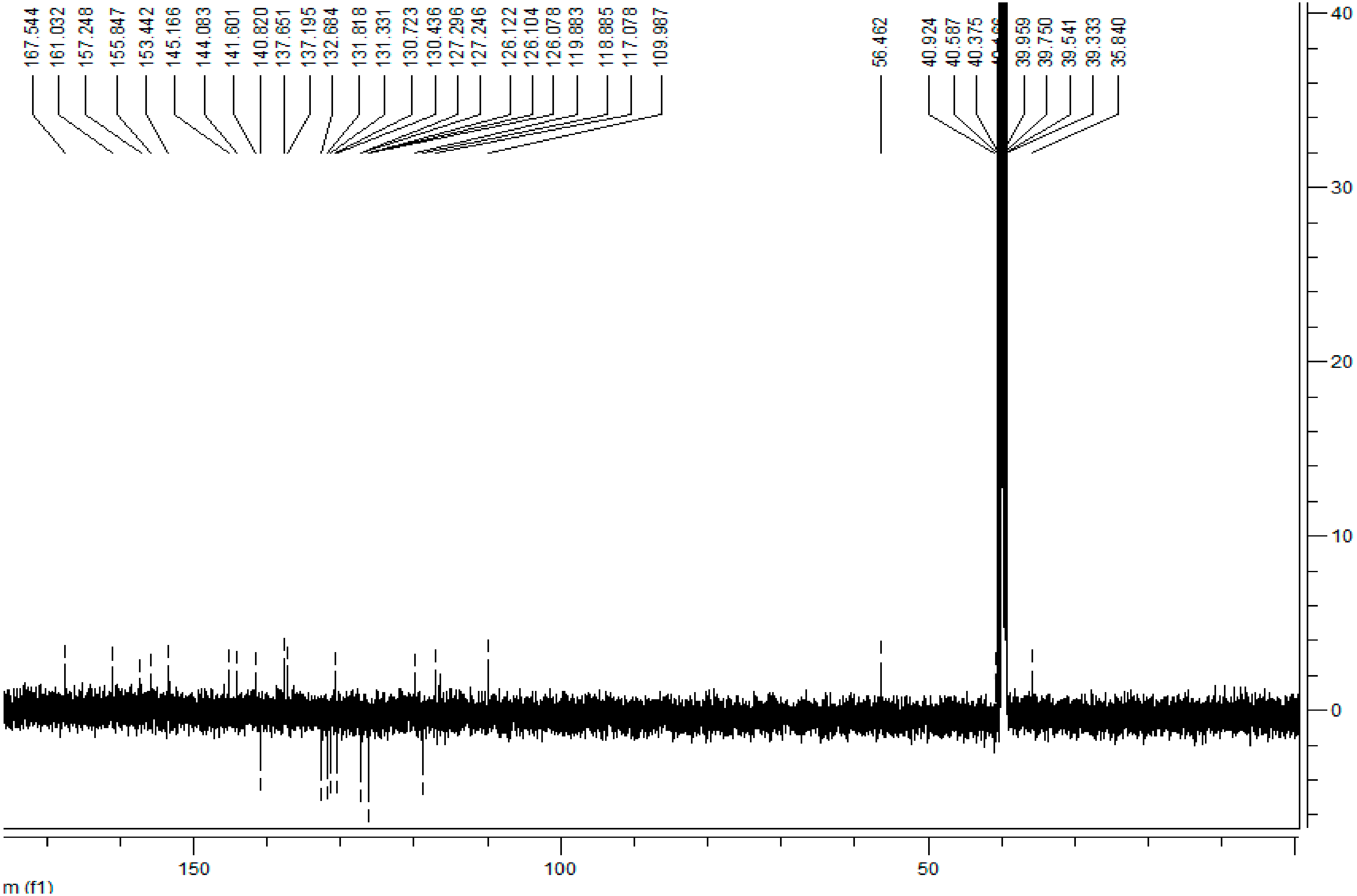

Figure S46: ^{13}C NMR (APT) spectra of compound **9b** (DMSO-d_6)

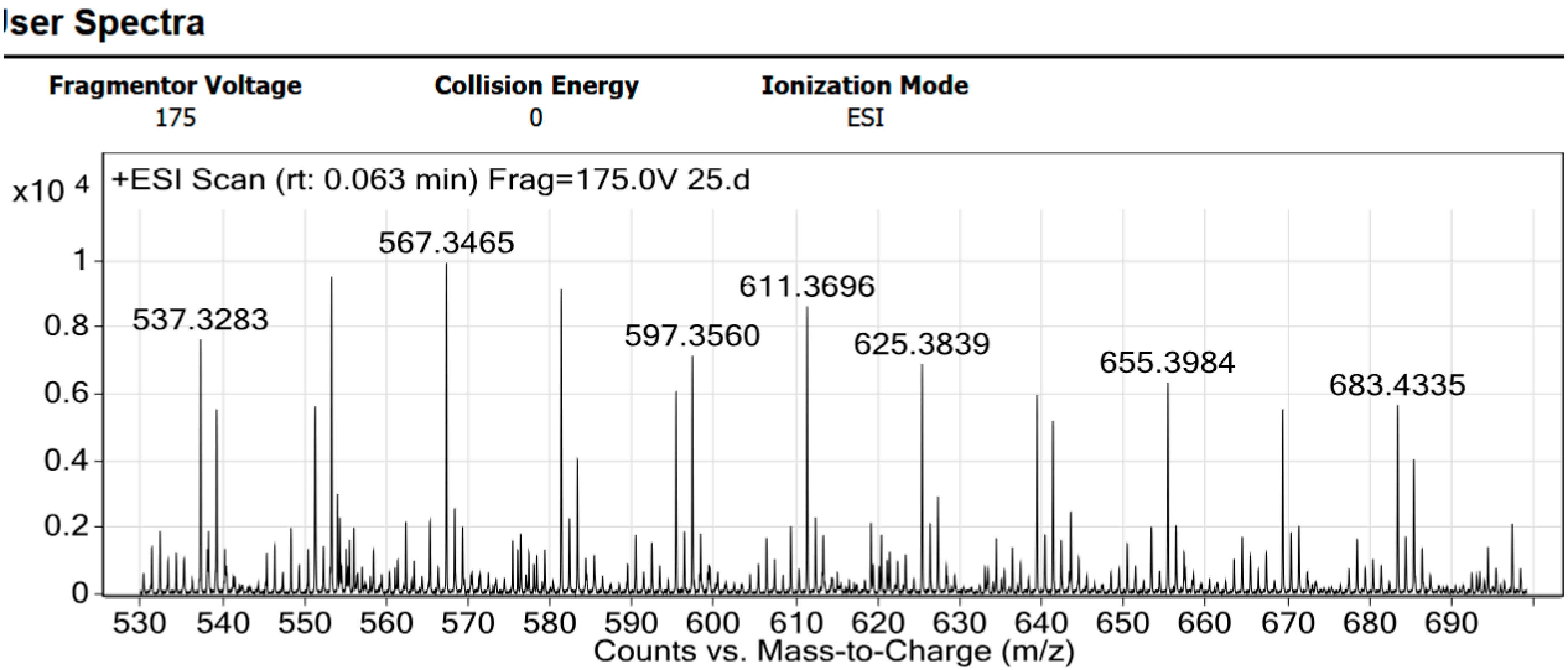

Figure S47: LC MS spectra of compound **9b**

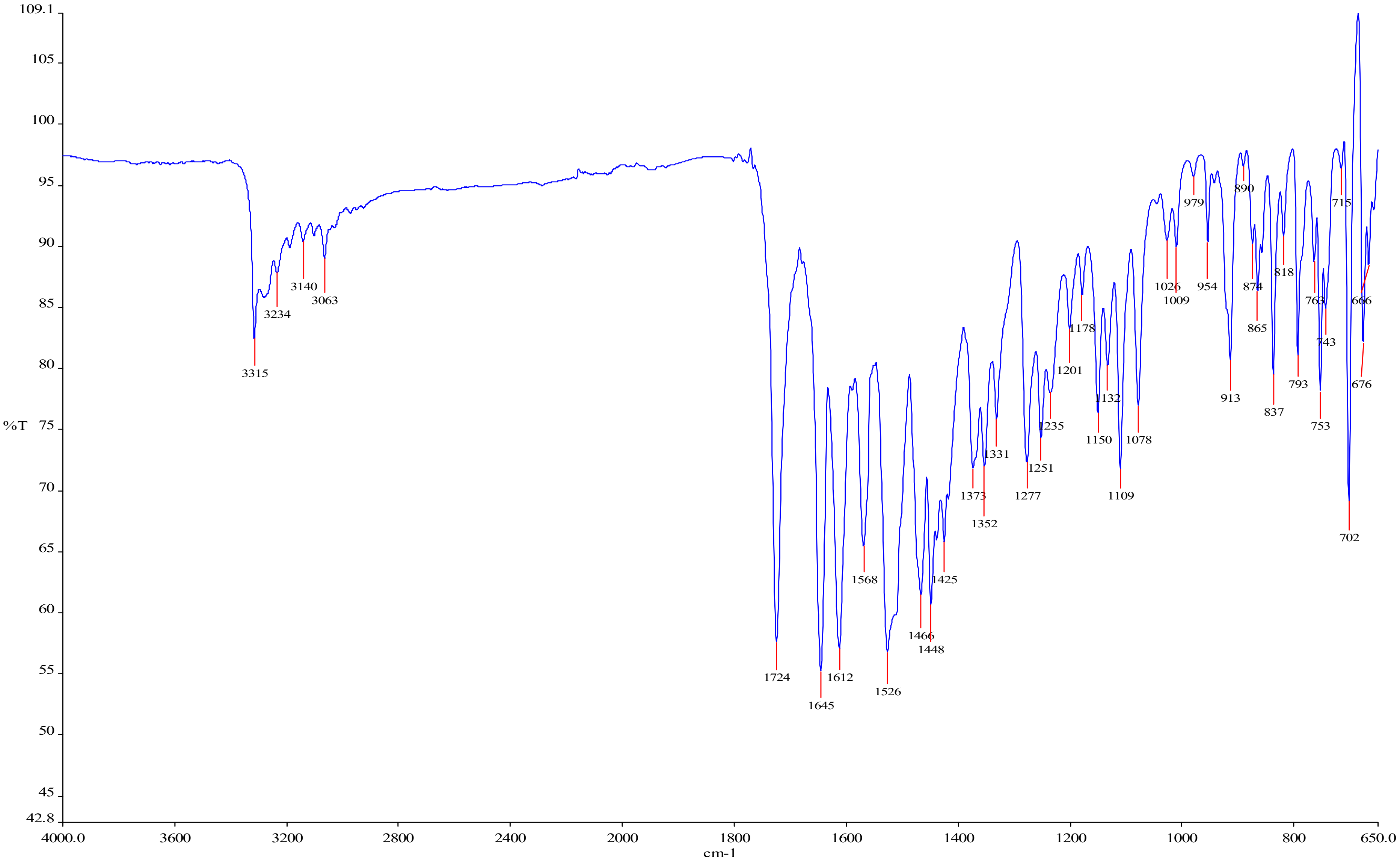

Figure S48: IR spectra of compound **9c**

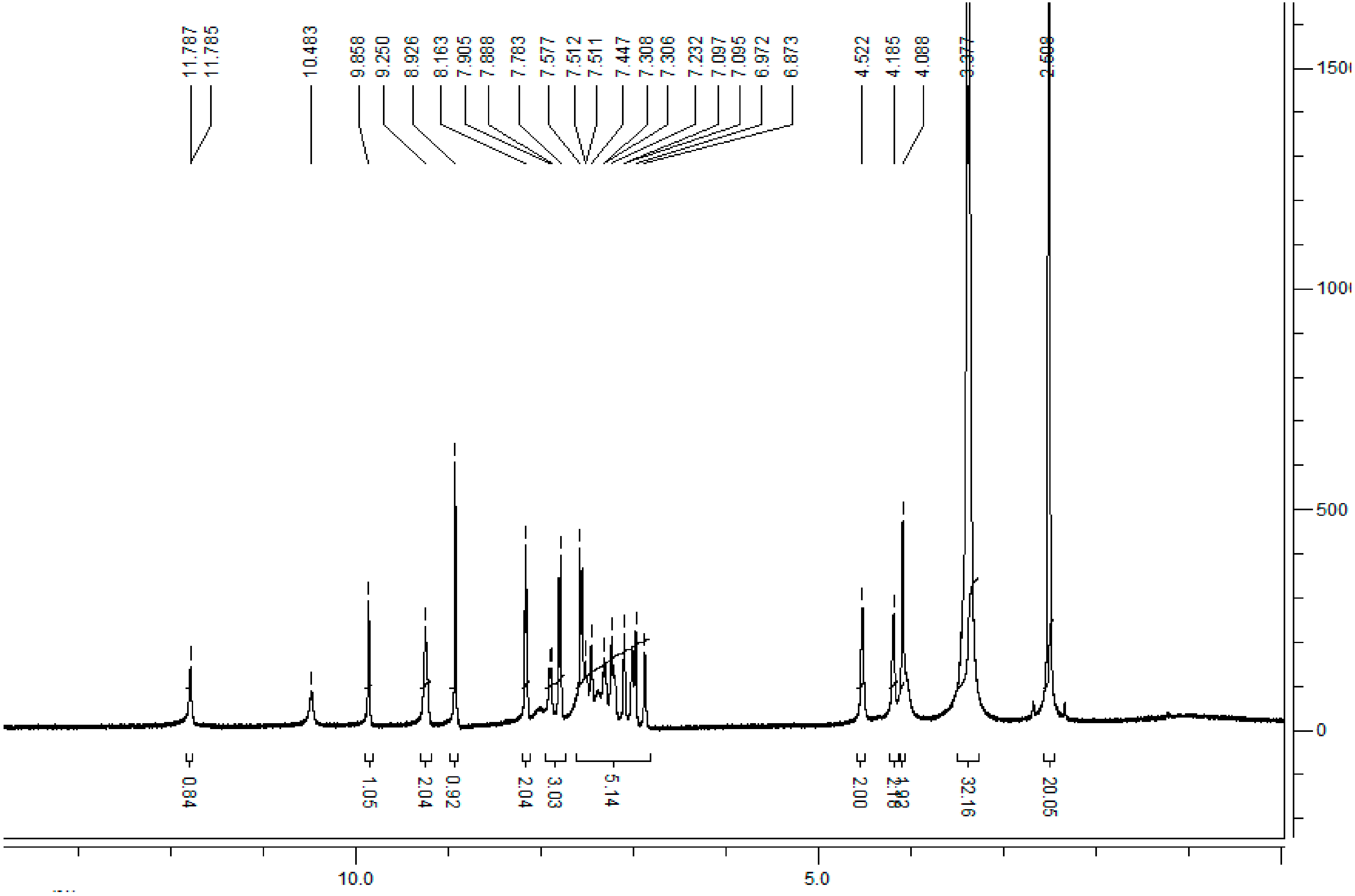

Figure S49: ^{1}H NMR spectra of compound **9c** (DMSO-d_6)

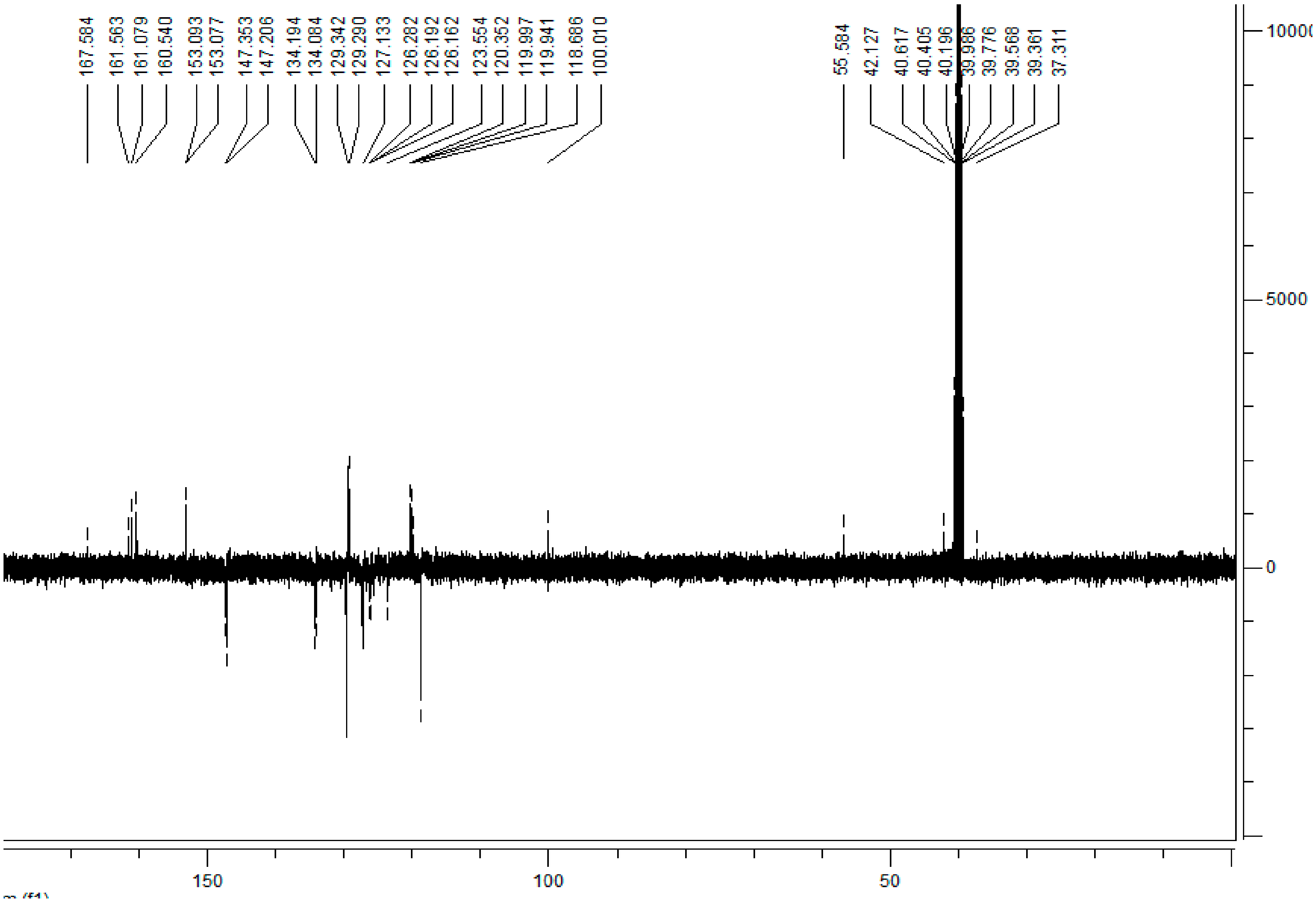

Figure S50: ^{13}C NMR (APT) spectra of compound **9c** (DMSO-d_6)

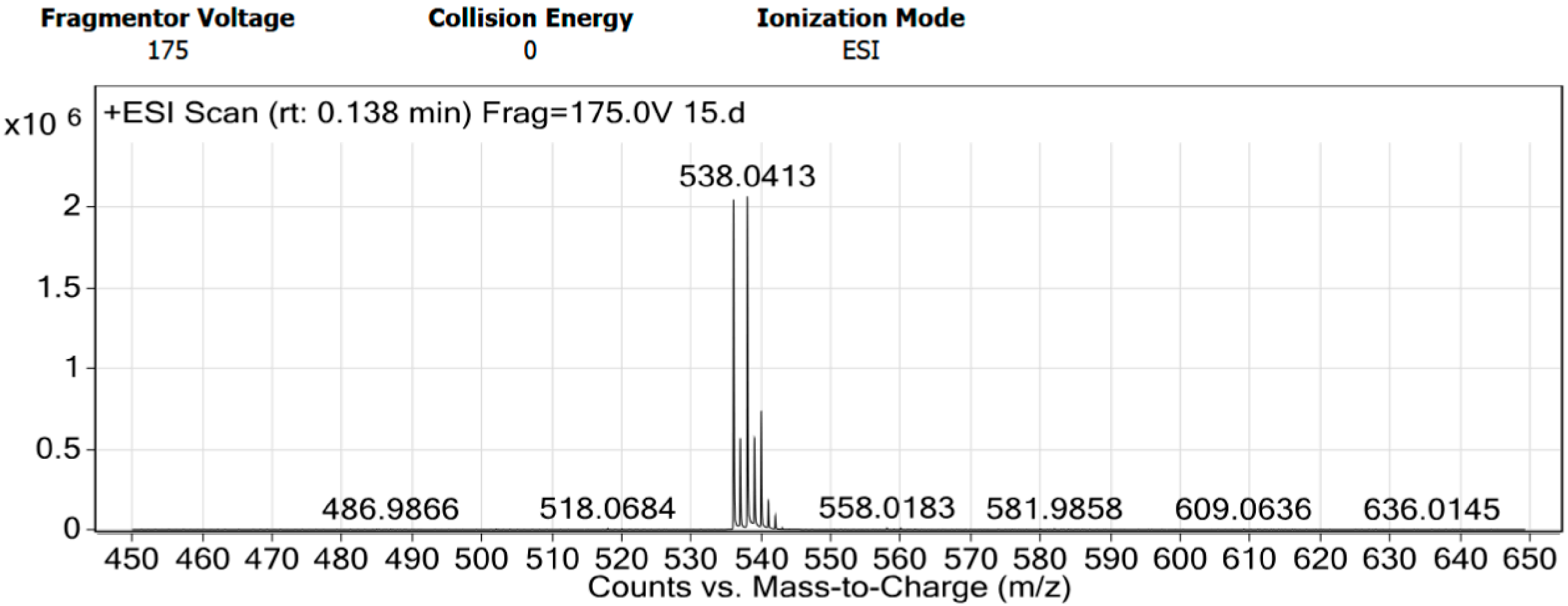

Figure S51: LC MS spectra of compound **9c**

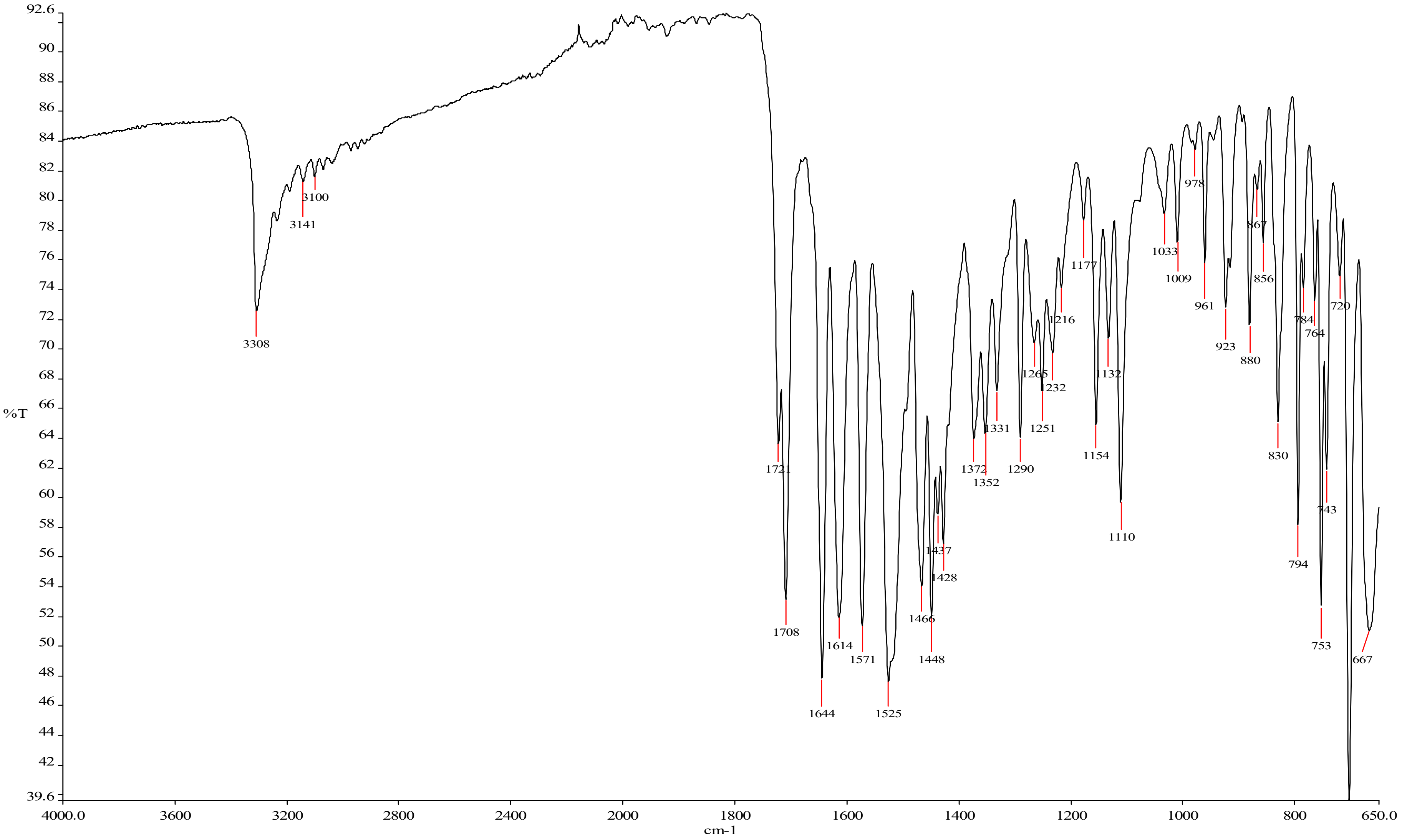

Figure S52: IR spectra of compound **9d**

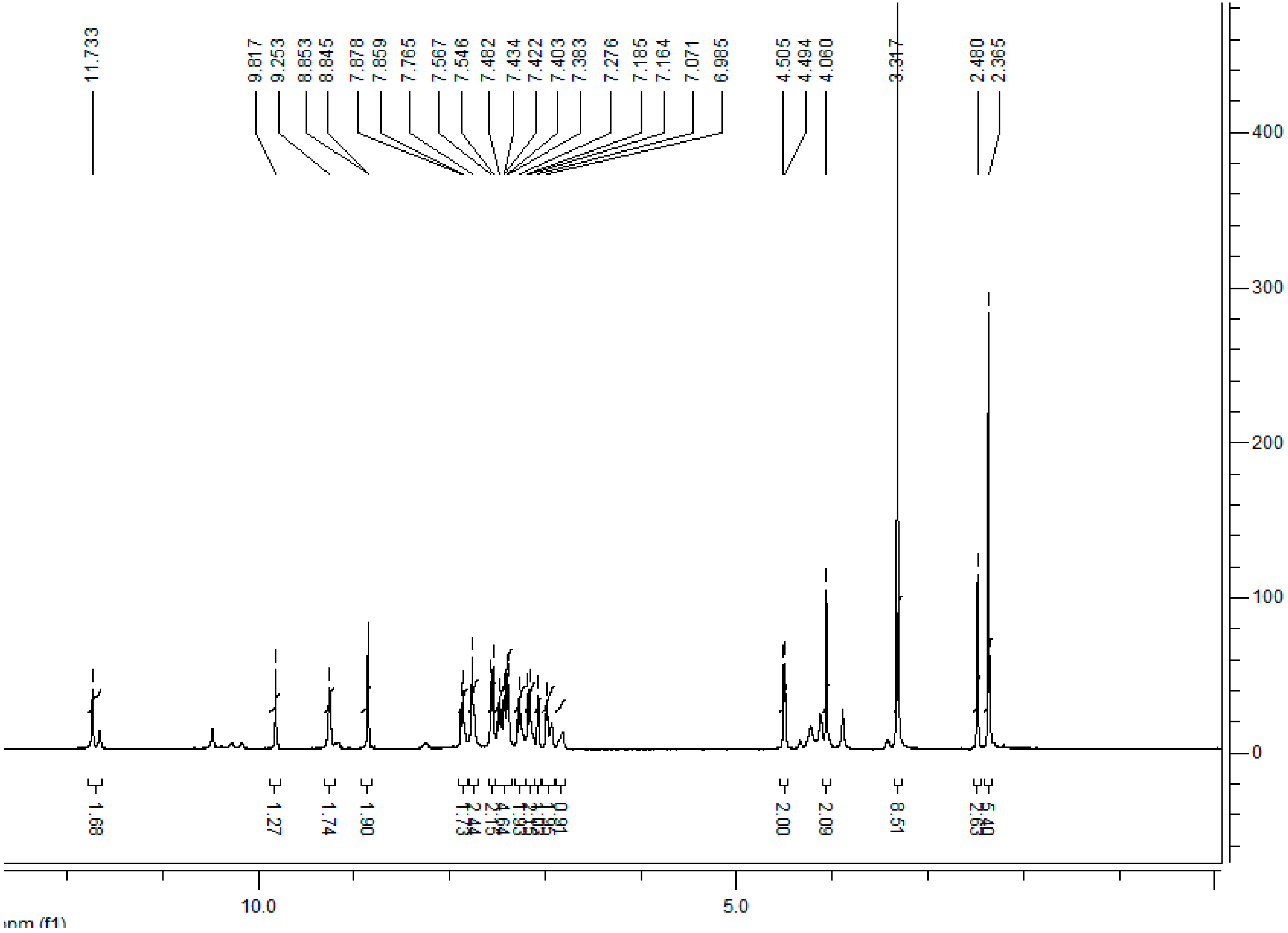

Figure S53: ^{1}H NMR spectra of compound **9d** (DMSO-d_6)

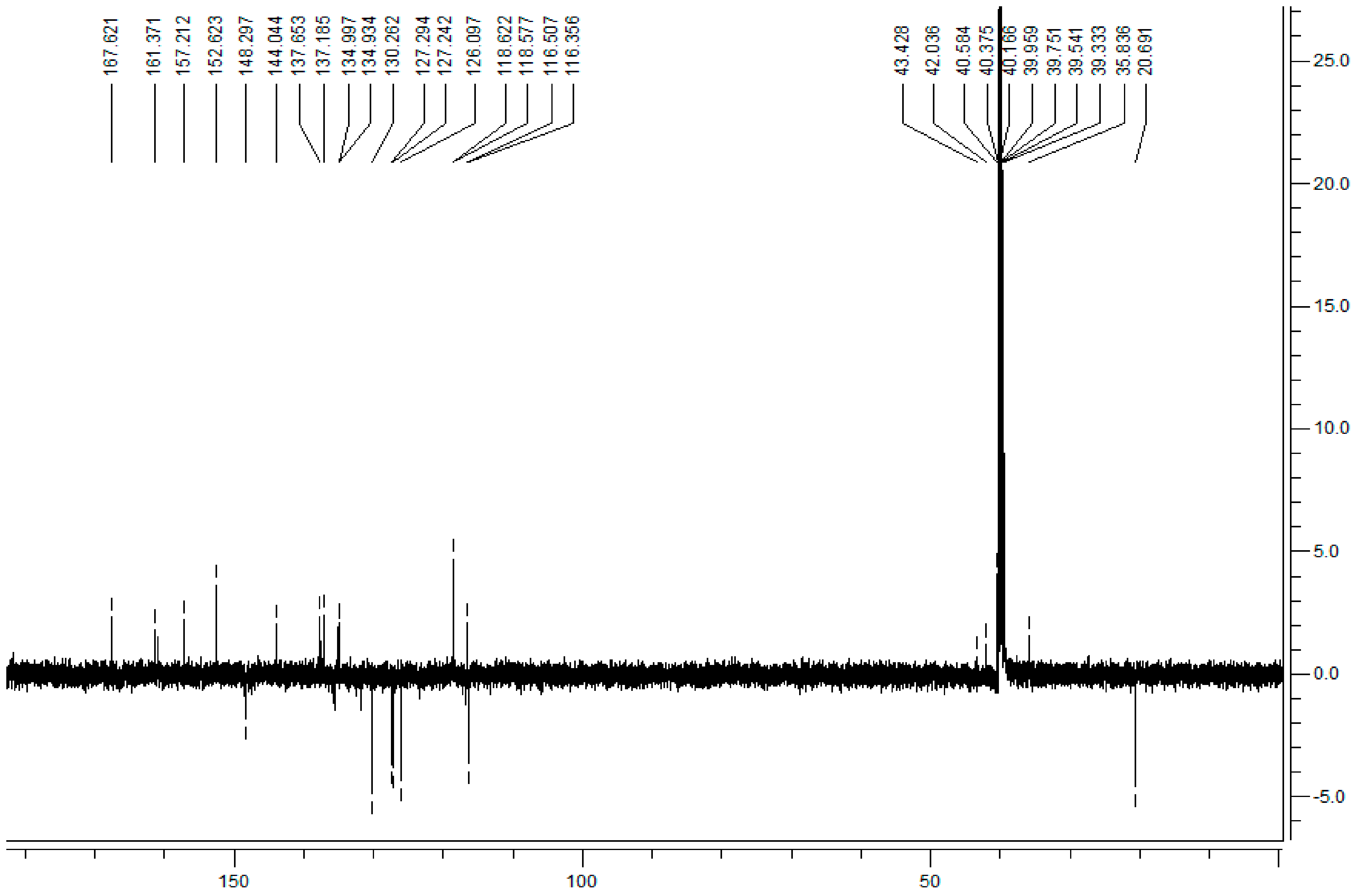

Figure S54: ^{13}C NMR (APT) spectra of compound **9d** (DMSO-d_6)

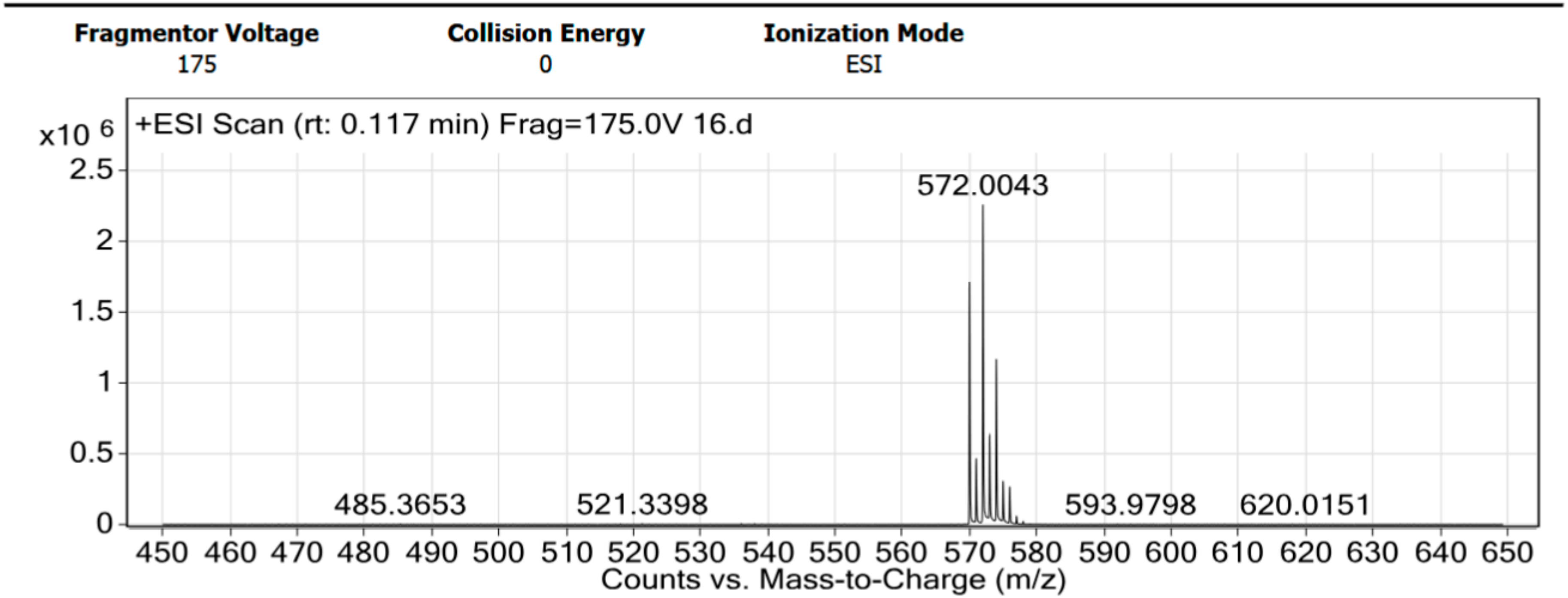

Figure S55: LC MS spectra of compound **9d**

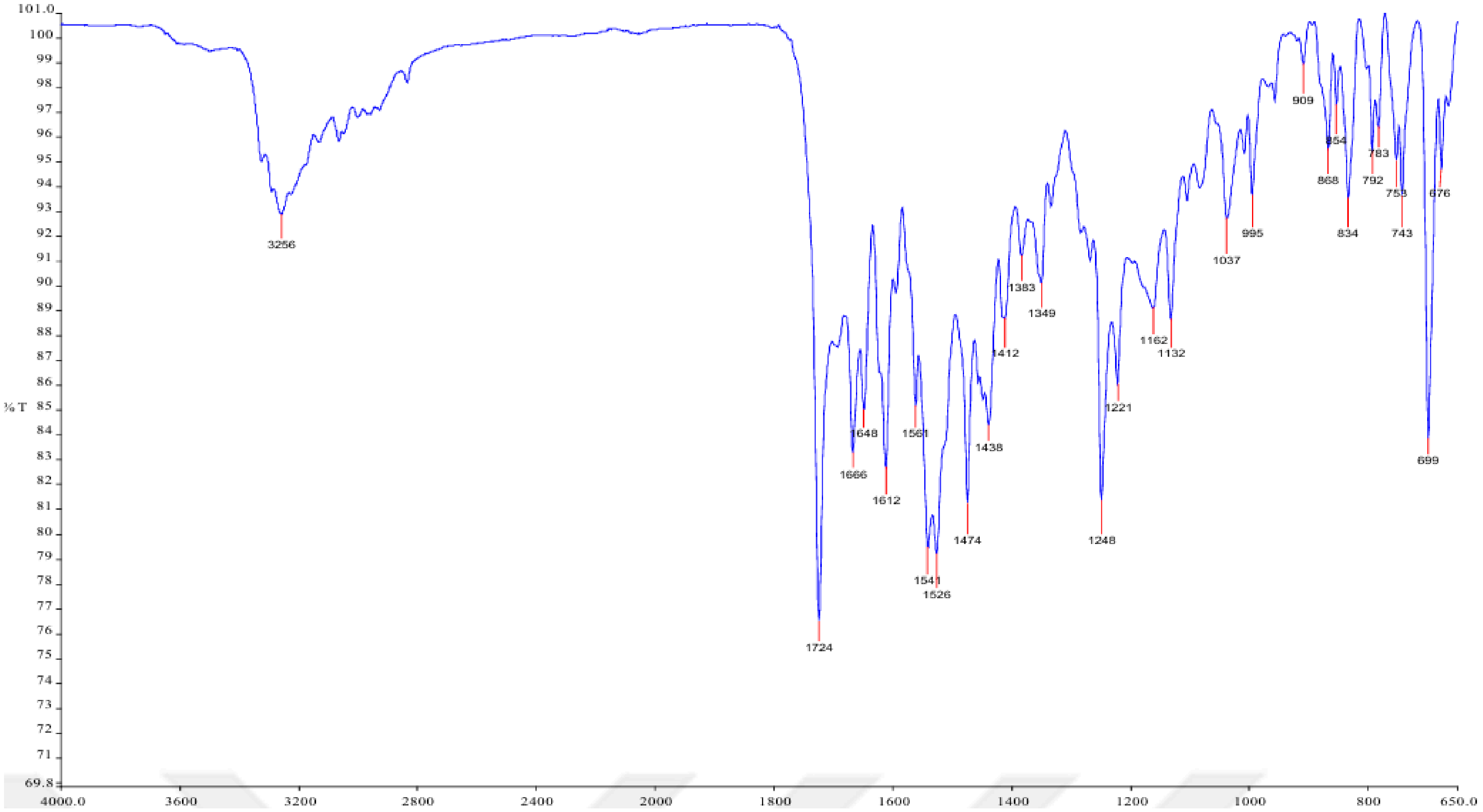

Figure S56: IR spectra of compound **9e**

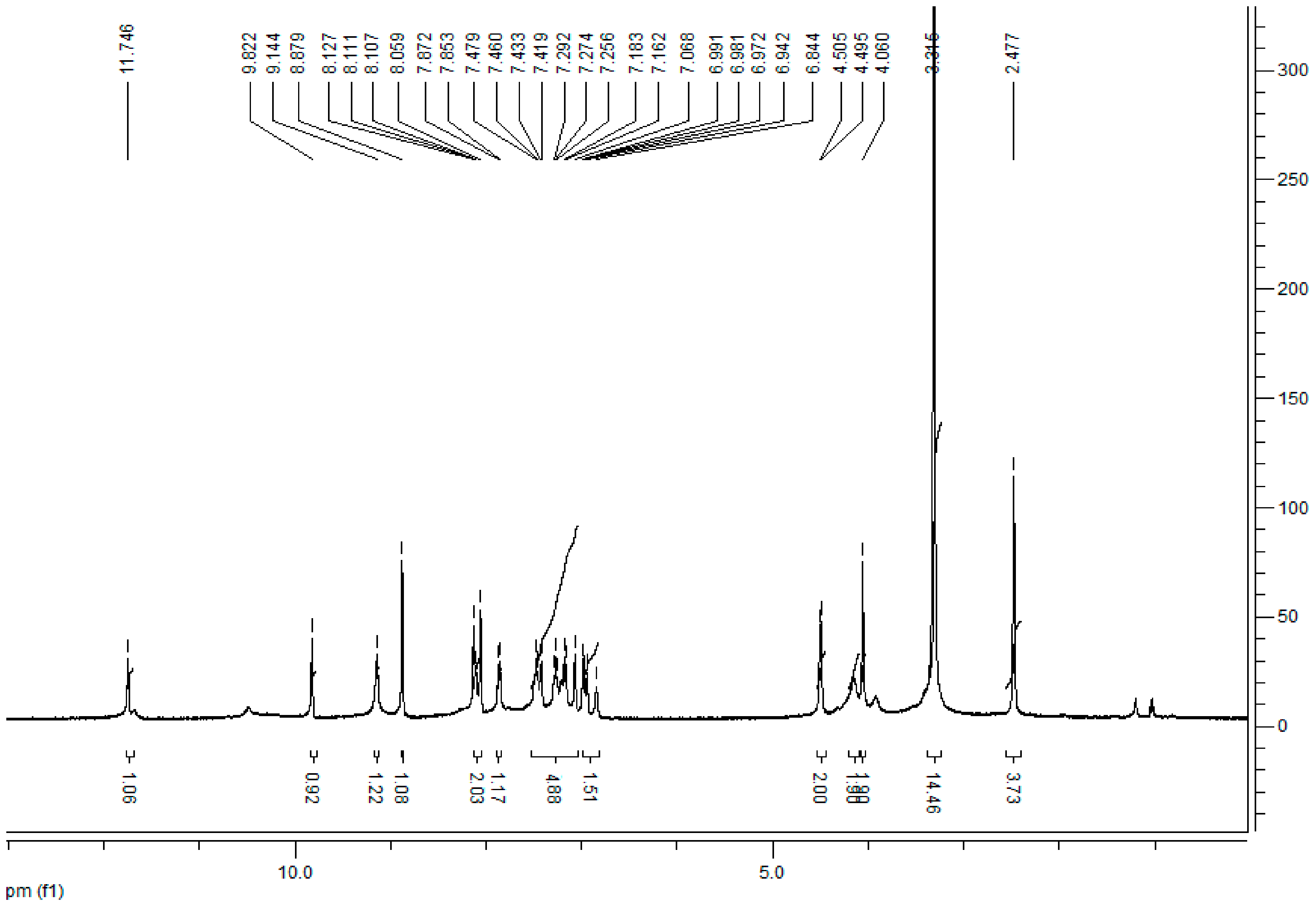

Figure S57: ^{1}H NMR spectra of compound **9e** (DMSO-d_6)

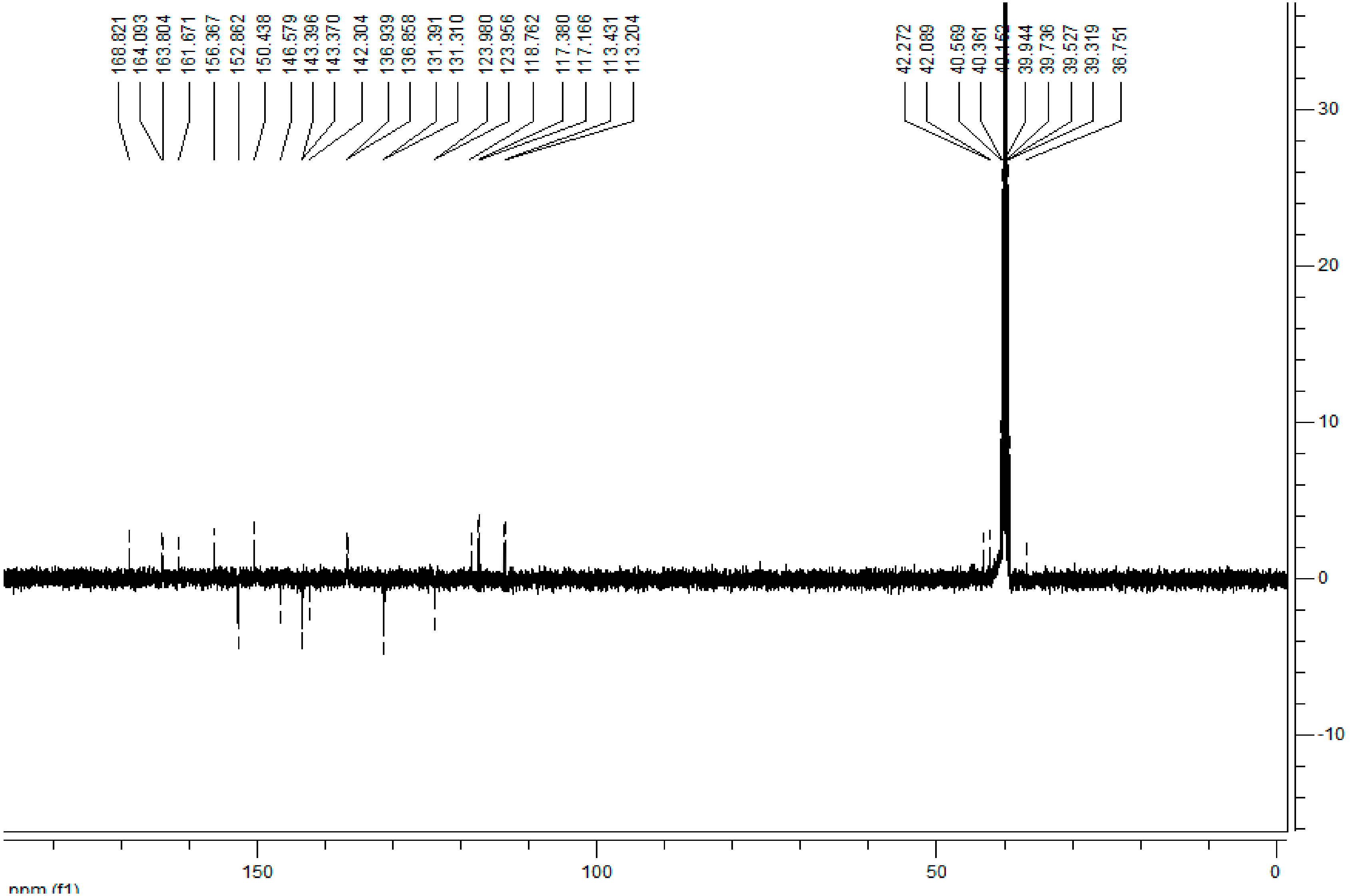

Figure S58: ^{13}C NMR (APT) spectra of compound **9e** (DMSO-d_6)

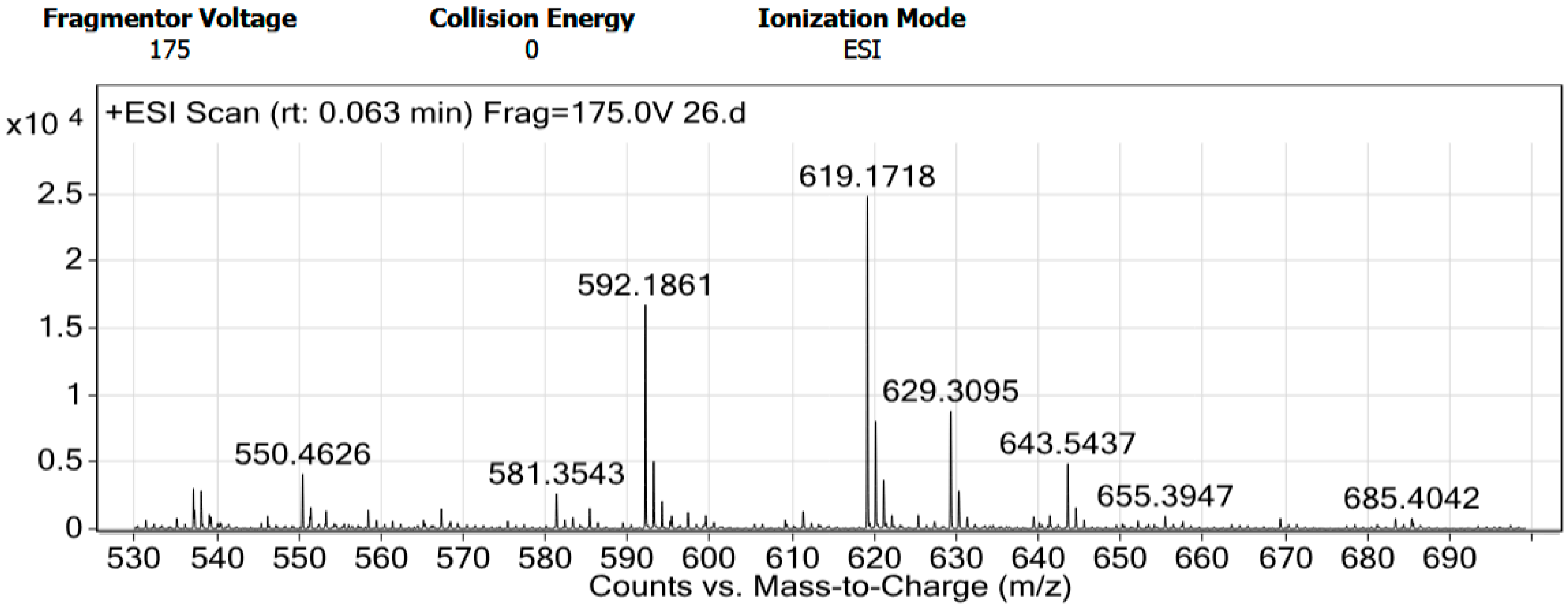

Figure S59: LC MS spectra of compound **9e**

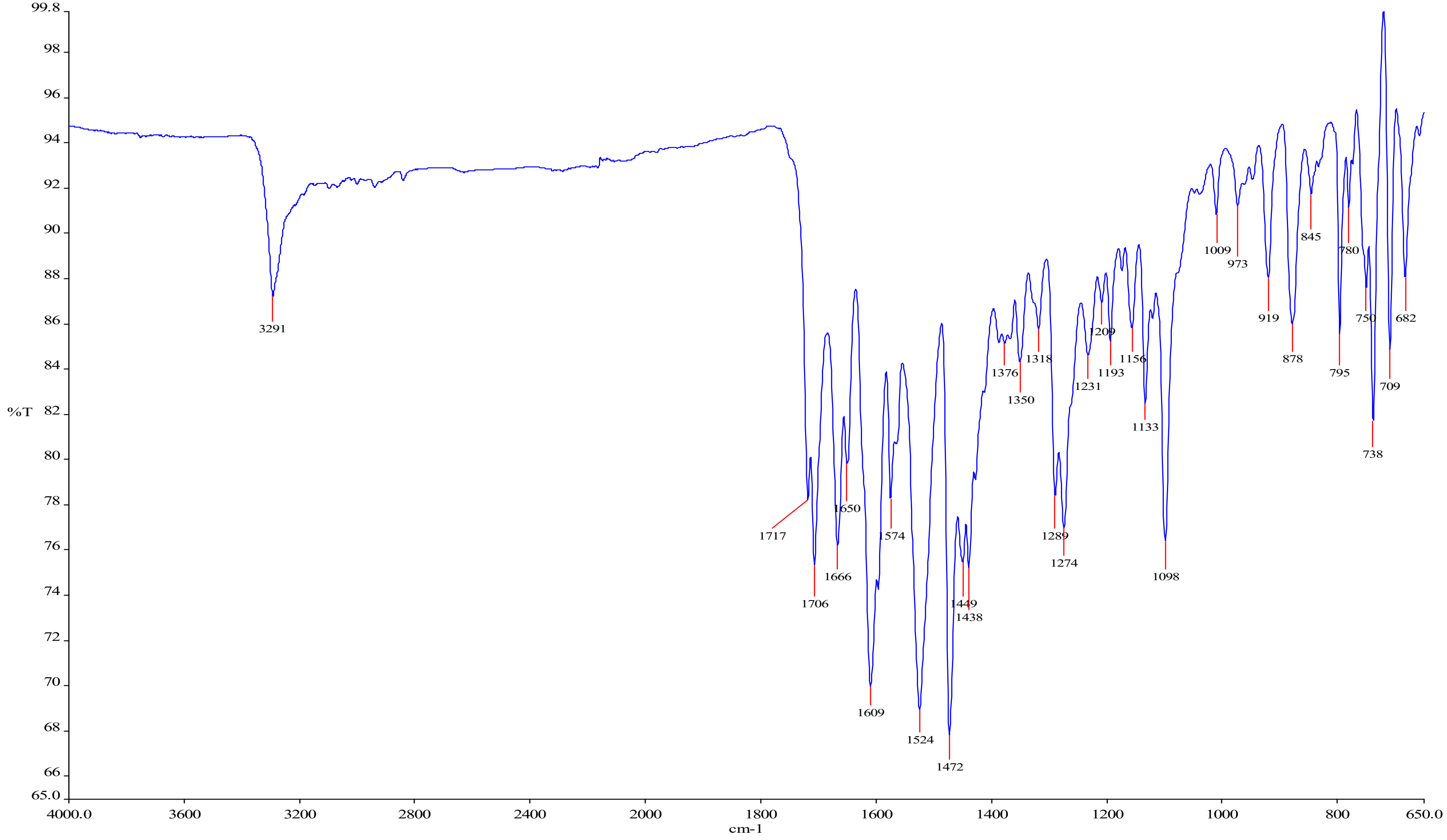

Figure S60: IR spectra of compound **9f**

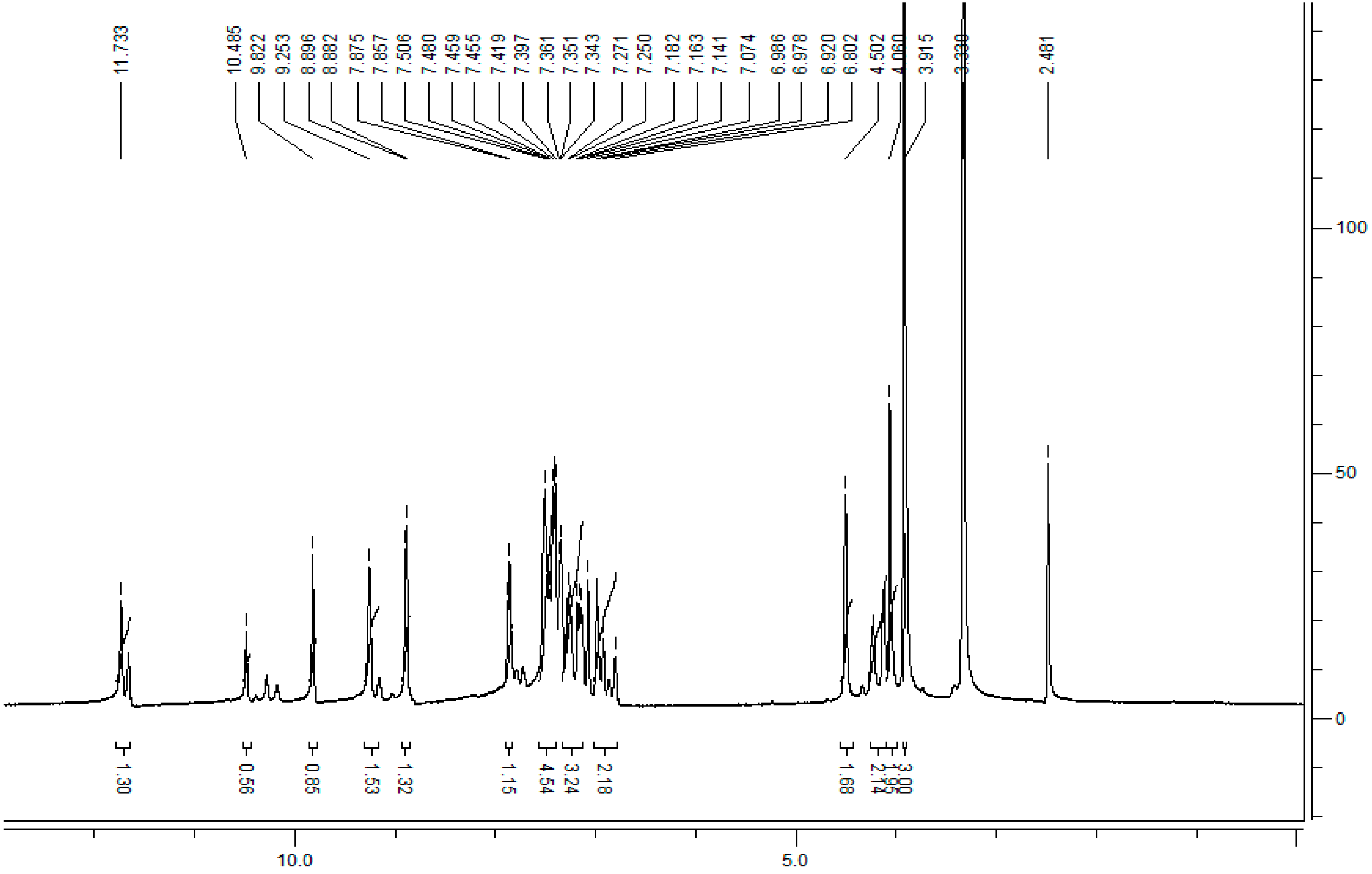

Figure S61: ^{1}H NMR spectra of compound **9f** (DMSO-d6)

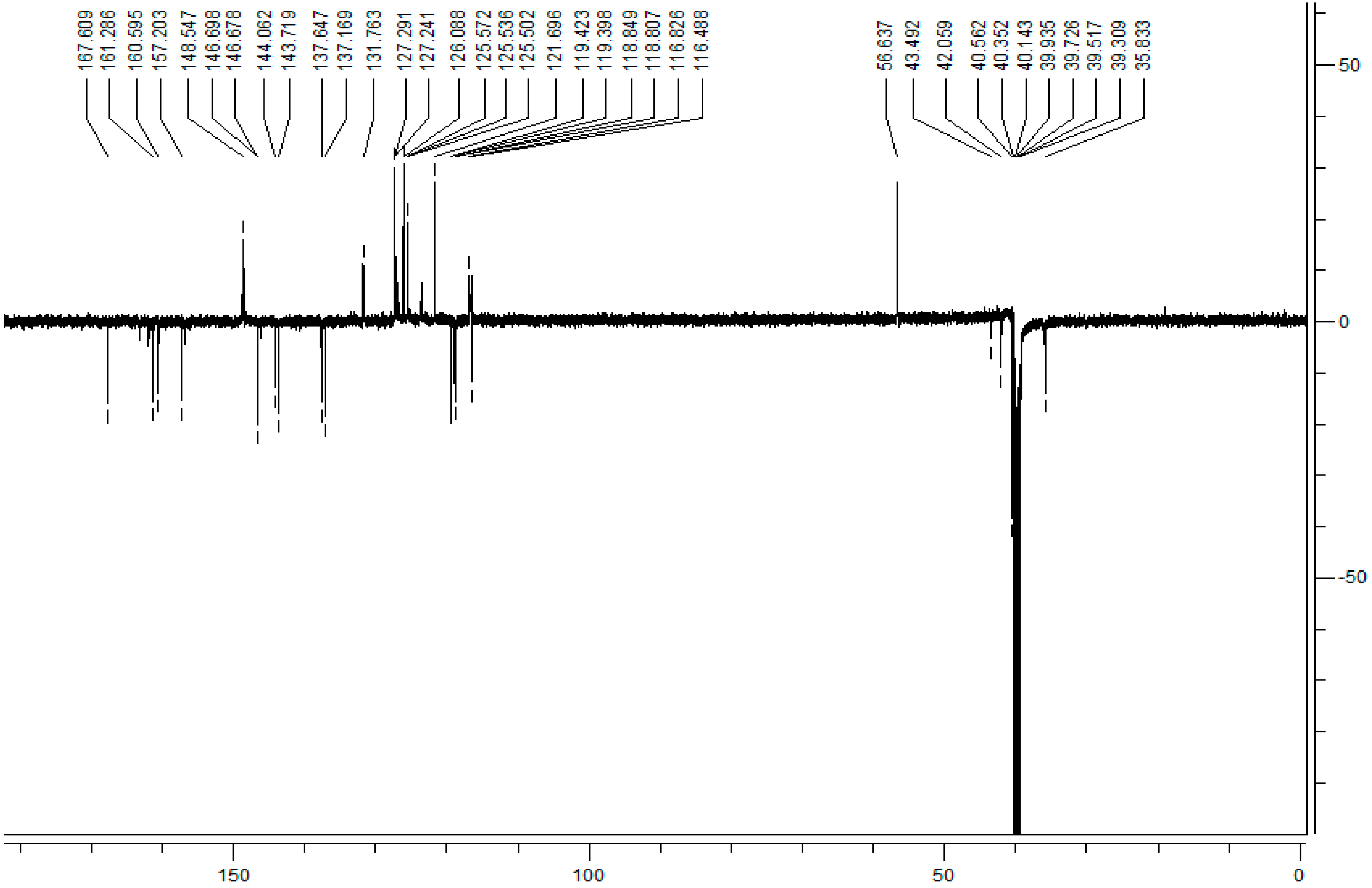

Figure S62: ^{13}C NMR (APT) spectra of compound **9f** (DMSO-d_6)
