# Supplementary material for: Synthesis and Anticancer Evaluation of Some Glycine Conjugated Hybrid Compounds Containing Coumarin, Thiophene and Quinazoline Moieties
Source: Pharmaceuticals (Basel). 2025 Oct 28;18(11):1627. doi: 10.3390/ph18111627 (PMC12654990; doi:10.3390/ph18111627)
Supplement: Supplementary file 1 [file pharmaceuticals-18-01627-s001.zip › Supplementary_TableS1.pdf]

Supplementary Table S1. Statistical analysis of IC_{50} values

IC_{50} values (µg/L) are presented as mean ± SD with 95% confidence intervals (CI).

Statistical significance was assessed by Student's t-test, comparing each compound with cisplatin. n=3 independent experiments.

Compound	PC-3 (Mean ± SD, 95% CI)	p vs Cis (PC-3)	MCF-7 (Mean ± SD, 95% CI)	p vs Cis (MCF-7)	HEK-293 (Mean ± SD, 95% CI)	p vs Cis (HEK-293)
9a	61.10 ± 1.01 [58.58, 63.62]	3.92e-06	80.23 ± 2.04 [75.16, 85.30]	3.85e-06	197.20 ± 2.03 [192.16, 202.24]	1.8e-08
9b	85.83 ± 2.02 [80.81, 90.85]	1.86e-06	104.30 ± 4.03 [94.28, 114.32]	5.94e-06	134.20 ± 4.01 [124.23, 144.17]	1.17e-06
9c	47.23 ± 2.04 [42.16, 52.30]	9.04e-05	71.07 ± 3.00 [63.61, 78.52]	1.93e-05	119.30 ± 1.13 [116.50, 122.10]	3.62e-08
9d	43.00 ± 3.00 [35.55, 50.45]	0.000581	74.10 ± 4.00 [64.15, 84.05]	3.58e-05	91.30 ± 1.13 [88.50, 94.10]	1.37e-07
9e	34.20 ± 2.03 [29.16, 39.24]	0.00204	46.03 ± 1.00 [43.55, 48.52]	4.94e-05	68.27 ± 4.03 [58.26, 78.27]	3.76e-05
9f	14.70 ± 1.00 [12.22, 17.18]	0.00105	16.17 ± 1.04 [13.58, 18.75]	0.0108	8.70 ± 0.50 [7.46, 9.94]	5.09e-05
